# A Systematic Review of Mental Health Nurses' Perceptions of Their Professional Identity

**DOI:** 10.1111/inm.70137

**Published:** 2025-09-24

**Authors:** Donagh O'Brien, John Richard Kelly, Peter Kelly

**Affiliations:** ^1^ Tallaght Mental Health Services, HSE Mary Mercer Health Centre Dublin Ireland; ^2^ Department of Psychiatry Trinity College Dublin and Tallaght Hospital, Trinity Centre for Health Sciences Dublin Ireland; ^3^ Department of Nursing and Midwifery Trinity College Dublin Dublin Ireland

**Keywords:** nurse's role, perception, psychiatric nursing, qualitative research, social identification

## Abstract

Evidence suggests that moving from specialist to generic nurse education, as is proposed in the United Kingdom and as has already happened in countries such as Australia, leads to a reduction in the quality of mental health nurse education and a weakening of mental health nurse professional identity. Meanwhile, research has found that mental health nurse professional identity is already complex because of the discipline's multifaceted but objectively ambiguous role. The present qualitative systematic review aimed to explore mental health nurses' perceptions of their professional identity. It was conducted in the form of a thematic analysis of qualitative data and was compliant with PRISMA reporting guidelines. Six databases (CINAHL Ultimate, EBSCO Medline, APA PsycInfo, APA PsycArticles, PubMed and SCOPUS) were searched in April and May 2024. The literature search yielded a total of *n* = 3710 studies. Following a process of screening using Covidence and quality appraisal using the CASP tool, a total of *n* = 23 peer‐reviewed qualitative studies were selected for inclusion. Five themes emerged: (1) Professional identity formation, (2) The attributes of mental health nurses, (3) The mental health nurse role, (4) The unique skills and knowledge of mental health nurses and (5) Professional identity and the future of mental health nursing. This systematic review concluded that mental health nurses recognise their profession as being complex and difficult to describe; however, they remain positive and committed to their roles, recognising that the uniqueness of mental health nursing may become its defining strength.

## 1 Introduction

In the UK, the shift from specialist nurse training in areas such as mental health, intellectual disability and children's nursing, towards generic training has become increasingly inevitable. Similar changes are under consideration in Ireland, while much of Europe already has established generic nurse training programmes (Warrender et al. 2024; Connell et al. 2022; NMC 2018; Hurley et al. 2024). This change in education standards for nursing in the UK is designed to make a depleted nursing workforce more flexible and better equipped to manage a population with a wide variety of co‐morbidities (Connell et al. 2022). However, evidence suggests that a generic nursing curriculum is likely to lead to a dilution of specialist nursing content such as mental health and a workforce insufficiently prepared to provide a high standard of specialist care (Haslam 2023; Reynolds et al. 2024; Warrender et al. 2024). Concern over the potential demise of mental health nursing in the UK because of nurse education changing from specialist to generic curricula is long standing and highlighted in past studies (Hurley and Ramsey 2008; Stickley et al. 2009).

It has been argued that the generic approach to nursing education is driven by neoliberal policies which prioritise financial targets and which are largely beyond the control of mental health nurses (Haslam 2023; Stickley et al. 2009). Universities have increasingly adopted a business‐oriented approach, whereby one generic nursing module costs less to run than multiple specialty nursing modules (Evans 2023). A move to generic nursing education in the UK arguably has good intentions, such as its aim to ensure that all nurses can provide more holistic and patient‐centred care to help people across their lifespan with a variety of illnesses, physical and mental (NMC 2018; Warrender et al. 2023). Early evidence, however, from combined or core nursing modules in the UK has shown a significant percentage reduction in discipline‐specific content being taught in these modules (Buescher and McGugan 2022; Reynolds et al. 2024).

From a historical perspective, the deinstitutionalisation of mental health care created a need to change the nature of mental health nursing education from a custodial model. This in turn saw mental health nursing as a specialty rendered outdated and led to the adoption of a generic nursing education model in territories such as Australia (Grant 2006). Australian studies have highlighted the negative impact that the introduction of generic nurse education has had on mental health nursing there since it replaced specialist nursing education in the 1980s.

Lakeman and Molloy (2018) have described a gradual corporatisation of nursing education in Australia, leading to a reduction in mental health nurse numbers and a rise in allied health professional numbers. Happell (2014) has described a gradual reduction in education standards for mental health nursing in Australia, with untrained staff teaching mental health nursing modules, thus degrading the profession and the Professional Identity (PI) of the remaining mental health nurse workforce. Therefore, the generic education model in Australia has reduced the status of mental health nursing as a nursing specialty and diminished its PI. Consequently, the apparent lack of a distinct identity has led to mental health nursing in Australia becoming unpopular as a career choice with nursing students and graduates, leading to recruitment and retention problems (Happell 2014; Harrison et al. 2017; Santangelo et al. 2018).

Generic nurse education prioritises the physical needs of patients and the teaching of psychomotor skills which are more easily quantifiable than vague and misunderstood specialist mental health nursing skills (Warrender et al. 2023). This is likely to have an impact on mental health service users who expect mental health nurses to be accessible, knowledgeable, to have time to spend with them, and to be able to relate to them as both professionals and friends (Jackson and Stevenson 2000). The unique skills of a mental health nurse which are located within a therapeutic nurse–patient relationship might also include conveying empathy, providing support, promoting equality and having self‐awareness (Browne et al. 2012). However, such skills risk being marginalised in a generic model of nursing, much to the detriment of service users (Haslam 2023).

Mental health nursing is perceived by some as unsophisticated and of low value, or ambiguous and invisible (Lakeman and Hurley 2021). The ambiguity of the mental health nurse role, due to its multifaceted nature, has had consequences for the credibility of the profession (Hurley and Lakeman 2021). Therefore, even before a move to generic nursing education in the UK seemed inevitable, mental health nursing arguably already risked damaging its specialist status due to an inability to justify itself through a clearly defined identity.

Mental health nursing should be able to distinguish itself from other branches of nursing and grow its own evidence base to justify its interventions (Warrender et al. 2023). If mental health nurses themselves cannot articulate and develop a clear‐cut identity, then governments, policy writers, other healthcare disciplines and the public will create that identity for them, and the future of the discipline will be in the hands of others (Lakeman and Hurley 2021). Already, in Sweden and some other countries, the government has deemed mental health nursing as no longer warranting regulation as a specialist nursing discipline (Gabrielsson et al. 2020). As of 2024, mental health care in Australia is characterised by chronic workforce shortages and underqualified staff, years after the adoption of generic nurse training (Hurley et al. 2024).

Given the complex and evolving landscape of PI in mental health nursing and its implications for the status of the profession and quality of mental health care, this systematic review builds on previous reviews (Da Cruz Piedade Oliveira et al. 2023; Hurley et al. 2022) to provide an up‐to‐date synthesis of the existing research base to potentially inform contemporary practice. The collected experience of mental health nurses adds a new dimension to the existing research and arguably gives the opportunity to advance knowledge on the subject.

### 1.1 Aim

The aim of this systematic review was to explore the perceptions or views or beliefs of mental health nurses about their PI. A secondary aim was to explore if mental health nurses felt happy and secure in their roles or saw a link between their PI and the future of mental health nursing. The systematic review was guided by the question: ‘What are the perceptions of mental health nurses of their professional identity?’

## 2 Methods

### 2.1 Study Design

This review consisted of a reflexive thematic analysis of qualitative data using the model designed by (Braun and Clarke 2022). The study was compliant with the Preferred Reporting Items for Systematic Reviews and Meta‐Analyses (PRISMA) statement (Page et al. 2021).

### 2.2 Search Strategy

Search terms were designed using a combination of free‐text words and the unique controlled language terms of each of the databases searched, linked with the Boolean Operators AND/OR. The search terms were adapted across different databases depending on each database's unique controlled language terms. The first reviewer independently conducted systematic literature searches in April and May 2024 of six databases, CINAHL Ultimate, EBSCO Medline, APA PsycINFO, APA PsycArticles, PubMed and SCOPUS.

### 2.3 Screening

Returned articles were screened using Covidence, based on predetermined inclusion and exclusion criteria. Studies were screened initially by title and abstract and following the elimination process, the remaining studies were screened by full text. Screening was carried out by all three reviewers.

### 2.4 Inclusion/Exclusion Criteria

Studies were exclusively included which contained qualitative data obtained through interview, direct observation or focus group. Mental health nurses from a wide variety of settings and contexts were included, such as specialist mental health or psychiatric nurses, generic trained nurses who work in mental health settings, community mental health nurses or community psychiatric nurses, and nurses working in child psychiatry. Studies which focused on PI in mental health nursing or mental health nursing roles were also included. Nurses who work in settings outside of mental health were excluded (Table 1).

**TABLE 1:** Inclusion/Exclusion criteria.

Inclusion criteria	
Sample	Mental health nurses or psychiatric nurses, of all ages, gender, ethnicity and stages of career, from all countries including undergraduate and postgraduate students. Generic trained nurses who work or identify as mental health nurses. Mental health nurses who work in specialist areas such as community or children's mental health nursing.
Phenomenon of interest	Professional identity in mental health or psychiatric nurses. Professional identity formation or development. Mental health or psychiatric nurse roles and duties. Definition of mental health nurse role or issues pertaining to mental health nurse role. Based on evidence that mental health nurse role is closely related to professional identity.
Design	All qualitative studies. Data obtained through interview or focus group.
Evaluation	Perceptions, views, beliefs, attitudes, experiences of sample.
Research type	Phenomenological studies most relevant but other types of qualitative research such as ethnographic studies or grounded theory studies for consideration if relevant to the research question.

Exclusion criteria	
Sample	All disciplines of nursing other than mental health nurses, General Nurses, Intellectual Disability Nurses, Midwives and Public Health Nurses. All other professional healthcare disciplines, medicine, social work etc. Healthcare assistants or nurse's aides. All other persons.
Phenomenon of interest	Phenomena unrelated to Professional Identity or role of mental health nurses.
Design	Quantitative research, review papers, Discussion papers, opinion papers, position papers.
Evaluation	Data obtained through questionnaire. Subjective opinion of researchers. Literature review.
Research type	Types of quantitative studies such as Correlational or Experimental research.

### 2.5 Quality Appraisal

Quality assessment of the final studies was carried out independently by the first reviewer using the Critical Appraisal Skills Programme (CASP) tool (CASP 2024; Long et al. 2021). The CASP tool consists of 10 questions, each of which focuses on a particular aspect of qualitative research methods and asks for a Yes, No or Cannot Tell answer to each question, except for question 10, which asks for an open‐ended answer (CASP 2024). The CASP checklist does not have a scoring system due to its proposed intention as an educational tool, but any study which gives a ‘no’ answer to any of the first three questions is probably of low quality (CASP 2024). While none of the appraised studies were judged to be of low quality, there were different levels of detail in the reporting of research methods, and some studies did not include verbatim quotations, but all appraised studies were judged to be valid and were therefore included (Table 2).

**TABLE 2:** Results of critical appraisal.

CASP questions										
Paper	(1) Was there a clear statement of the aims of the research?	(2) Is the qualitative methodology appropriate?	(3) Was the research design appropriate to address the aims of the research?	(4) Was the recruitment strategy appropriate to the aims of the research?	(5) Was the data collected in a way that addressed the research issue?	(6) Has the relationship between researcher and participants been adequately considered?	(7) Have ethical issues been taken into consideration?	(8) Was the data analysis sufficiently rigorous?	(9) Is there a clear statement of findings?	(10) How valuable is the research?
(1) Terry (2020)	Yes	Yes	Yes	Yes	Yes	Yes	Yes	Yes	Yes	Valuable
(2) Hurley (2009)	Yes	Yes	Yes	Yes	Yes	Yes	Yes	Yes	Yes	Valuable
(3) Hurley and Lakeman (2011)	Yes	Yes	Yes	Yes	Yes	Cannot tell	Yes	Yes	Yes	Valuable
(4) McCrae et al. (2014)	Yes	Yes	Yes	Yes	Yes	Yes	Yes	Yes	Yes	Valuable
(5) Crawford et al. (2008)	Yes	Yes	Yes	Yes	Yes	Yes	Yes	Yes	Yes	Valuable
(6) Hercelinskyj et al. (2014)	Yes	Yes	Yes	Yes	Yes	Yes	Yes	Yes	Yes	Valuable
(7) Moir and Abraham (1996)	Yes	Yes	Yes	Yes	Yes	No	No	Cannot tell	Yes	Valuable
(8) Santangelo et al. (2018)	Yes	Yes	Yes	Yes	Yes	Cannot tell	Yes	Yes	Yes	Valuable
(9) Harrison et al. (2017)	Yes	Yes	Yes	Yes	Yes	Yes	Yes	Yes	Yes	Valuable
(10) Wand et al. (2022)	Yes	Yes	Yes	Yes	Yes	Yes	Yes	Yes	Yes	Valuable
(11) Rasmussen et al. (2017)	Yes	Yes	Yes	Yes	Yes	Yes	Yes	Yes	Yes	Valuable
(12) White and Kudless (2008)	Yes	Yes	Yes	Yes	Yes	Yes	Yes	Yes	Yes	Valuable
(13) Humble and Cross (2010).	Yes	Yes	Yes	Yes	Yes	Yes	Yes	Yes	Yes	Valuable
(14) Holyoake (2002).	Yes	Yes	Yes	Yes	Yes	Yes	Yes	Yes	Yes	Valuable
(15) Reis et al. (2023).	Yes	Yes	Yes	Yes	Yes	Yes	Yes	Yes	Yes	Valuable
(16) Buescher and McGugan (2022).	Yes	Yes	Yes	Yes	Yes	Yes	Yes	Yes	Yes	Valuable
(17) Karanikola et al. (2018).	Yes	Yes	Yes	Yes	Yes	Cannot tell	Yes	Yes	Yes	Valuable
(18) Bray (1999)	Yes	Yes	Yes	Yes	Yes	Yes	Yes	Yes	Yes	Valuable
(19) Sercu et al. (2015).	Yes	Yes	Yes	Yes	Yes	Cannot tell	Yes	Yes	Yes	Valuable
(20) Deady (2005).	Yes	Yes	Yes	Yes	Yes	Yes	Yes	Yes	Yes	Valuable
(21) Rungapadiachy et al. (2004).	Yes	Yes	Yes	Yes	Yes	Yes	Yes	Yes	Yes	Valuable
(22) Savio (1991).	Yes	Yes	Yes	Yes	Yes	Cannot tell	No	Cannot tell	Yes	Valuable
(23) Barlow (2006).	Yes	Yes	Yes	Yes	Yes	Yes	Yes	Yes	Yes	Valuable

### 2.6 Data Extraction and Analysis

Data was extracted in the form of verbatim quotations or narrative accounts of key ideas from the text. Data was extracted initially by the first reviewer and cross‐checked for accuracy by the second reviewer, following which the data was recorded manually on data extraction forms. Data analysis was carried out independently by the first reviewer, using Reflexive Thematic Analysis as a guideline (Braun and Clarke 2022). Reflexive thematic analysis is a process in 6 phases. Phase 1: Familiarising oneself with the data, Phase 2: Coding, Phase 3: Generating the initial themes, Phase 4: Developing and reviewing the themes, Phase 5: Refining, defining and naming the themes, and Phase 6: writing up the findings (Braun and Clarke 2022).

## 3 Results

**FIGURE 1:**
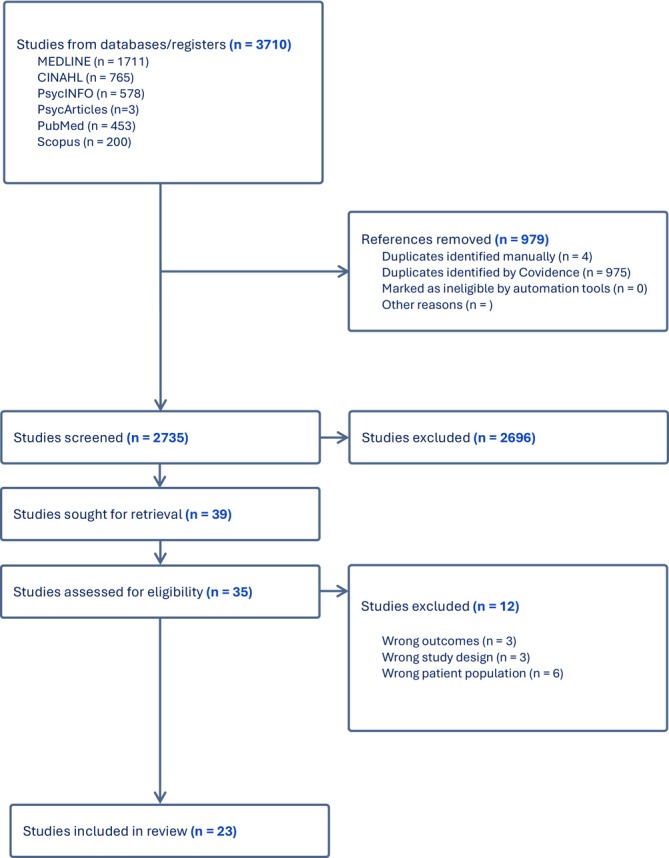
PRISMA flow chart.

Database searches yielded a total of 3710 studies. Following the screening by title and abstract by the primary and secondary reviewers, the remaining 39 studies were screened by full text by the first and second reviewers, after which consensus was reached on the inclusion of a total of 23 peer‐reviewed studies. Most of the studies included qualitative data which was transcribed verbatim and presented in the form of quotes from research participants. However, some of the included studies consisted of narrative reports of their findings without using quotations (Savio 1991; Deady 2005; Santangelo et al. 2018) (Figure 1; Table 3).

**TABLE 3:** Study characteristics.

References	Aim	Design	Population	Findings
(1) Sercu et al. (2015) Belgium.	To explore how stigma might give meaning to mental health nurse identities.	Qualitative. Case Study Design.	33 mental health nurses, working in dual diagnosis (drugs/mental health) in Ghent, Belgium.	Participants chose mental health nursing because of favourable contrast to general nursing. Some participants are against labelling as it increases stigma. Others think labelling helps guide treatment.
(2) Barlow (2006) Scotland.	To explore self‐perceived differences between CPN and other team members contribution in an MDT.	Qualitative.	4 CPNs (and 5 other staff members) working in PLL community service.	CPNs feel they have personal attributes and skills which are unique to them and render them suited to job. Some feel they are undervalued. CPN role is hard to define. Other staff value CPN highly on the team. Other staff think that they do the same job as CPN other than giving injections.
(3) Rungapadiachy et al. (2004) England.	To examine role of mental health nurse from point of view of student nurses.	Qualitative. Grounded Theory.	14 mental health nursing students.	Mental health nurses observed by student nurses and perceived as fulfilling many roles such as ward administration, medication administration, physical duties etc. Some mental health nurse activities important but invisible. Some nurses engage in unprofessional or non‐therapeutic activities.
(4) Deady (2005). Ireland	To ascertain if there is a collective set of values among Irish psychiatric nurses.	Qualitative. Phenomenological approach.	8 Irish mental health nurses, 4 male, 4 women.	Psychiatric nurses see themselves as different to other disciplines, working within unsupportive system but with superior interpersonal skills. Psychiatric nurses have progressive attitudes and embrace working in community setting.
(5) Karanikola et al. (2018) Cyprus.	To investigate the lived experience of Greek‐Cypriot CMHNs of their professional role.	Qualitative. Phenomenological.	5 CMHNs working in Cyprus.	Challenges of conducting home visits. Value as well as challenges of working autonomously. Value and importance of interventions like therapeutic relationship.
(6) Rasmussen et al. (2017) Denmark/Australia.	To examine the practicality of using a conceptual framework for inpatient CAMHS in other areas.	Qualitative. Exploratory within a social constructivist context.	9 CAMHS nurses working in 2 locations, 1 in Denmark and 1 in Tasmania. All women.	Differences between training pathways for CAMH nurses in Denmark and Tasmania. Mental health qualification useful in both territories. CAMH nurses find support and identity in shared knowledge pool. CAMH nurse feel undervalued.
(7) Terry (2020) Wales.	To examine ‘talk’ about mental health nurse identities from multiple perspectives.	Qualitative.	17 mental health nurses. 16 women and 1 man.	Mental health nurses take on role of care‐coordinator, to the detriment of their nursing work. Mental health nurses have diminished status, ambiguous role, jack‐of‐all‐trades, limited respect from colleagues and families.
(8) Savio (1991) Italy	To investigate if professional identity of psychiatric nurses has changed since moving from hospital to community setting.	Qualitative.	9 psychiatric nurses, recently moved from a hospital to community setting in Piedmont, Italy.	Differences in nursing activities between hospital and community settings. Participants identify progressive nature of community work but felt more autonomous in the community due to familiarity with the environment.
(9) Holyoake (2002) England.	To examine if gender representations affect mental health nurses' identity.	Qualitative. Ethnography.	Male mental health nurses working in 3 English mental health units.	Male identity still an issue in mental health nursing. More men in mental health nursing due to traditional view of custodial practice. Stereotypes of male nurses being in touch with feminine side or gay. Male nurses would rather be recognised for their work.
(10) Hurley (2009) England/Scotland.	To improve understanding of mental health nurse identities.	Qualitative. Phenomenological.	24 mental health nurses. 13 female, 11 male.	Aspects of mental health nurse role. Varied role, patient focused, therapeutic use of self, common sense approach, borrowing and repackaging skills from other disciplines.
(11) Hurley and Lakeman (2011) England/Scotland.	To understand the formation of mental health nurse's personal and professional identity.	Qualitative. Direct Phenomenology.	24 mental health nurses working in NHS settings in England and Scotland.	Mental health nurse identity journey, starts with training, role models, then learning new skills and changing identity to new specialist role such as CBT therapist or CMHN.
(12) Crawford et al. (2008) England.	To examine how community mental health nurses (CMHNs) perceive their working lives.	Qualitative. Thematic Analysis.	34 CMHN from various locations in Midlands, England.	CMHN identity hard to define but centred on patient wellbeing. CMHNs uncertain about their professional status. Upskilling as s possible exit strategy. Some gender stereotyping. The need for recognition.
(13) Buescher and McGugan (2022) England.	To examine PI formation in mental health nursing students undertaking core modules.	Qualitative. Dialogical Narrative Analysis.	6 mental health nursing students, different levels of training. All women.	Mental health nursing students marginalised or made to feel different when they have core nursing modules with general nursing students. Core module lecturers have poor level of knowledge of mental health.
(14) Reis et al. (2023) Brazil.	To understand the PI of mental health nurses.	Qualitative. Merleau‐Ponty phenomenology of experience.	16 mental health nurses working in 4 psychosocial care settings.	Mental health nurses trying to establish a new identity in psychosocial care setting. Role in hospital setting was more familiar and identity based on technical duties. Patient centred approach in psychosocial setting more appealing.
(15) Wand et al. (2022) Australia.	To explore perspectives of MHNs and others on the present and future status of MHN.	Qualitative. Thematic Analysis.	5 MHNs (3 consumer representatives and 3 allied health professionals) in a local health district in Sydney.	MHN role incorporates health promotion. MHN highlighted collaborative, therapeutic relationship with patients. MHN have attributes like flexibility and versatility. MHN undervalued. MHN role should include better professional development and less emphasis on bureaucracy.
(16) Moir and Abraham (1996) Scotland	To examine how psychiatric nursing students construct occupational identity	Qualitative. Discourse Analysis.	20 total. 10 1st year and 10 final year nursing students.	Choice of mental health nursing as a career based on differences with general nursing. General nursing too technical, too restrictive. Mental health nursing more patient focused, students feel more valued and respected.
(17) McCrae et al. (2014) England.	To explore facilitators and barriers to professional identity in newly qualified postgrad mental health nurses.	Qualitative. Grounded Theory.	10 newly qualified mental health nurses just finished postgraduate course. 9 female and 1 male.	Reason for choosing mental health nursing. Mental health nurse role hard to define. Tolerance, flexibility etc., useful traits for MHN. Role models during training. Academic status of MHN appealing for some students and helped enhance PI. Pride in becoming MHN. Public sector v private sector.
(18) Santangelo et al. (2018) Australia.	To explore mental health nursing in the context of its vague PI.	Qualitative. Constructivist Grounded Theory.	36 mental health nurses, with 5 clients and 1 healthcare provider.	Mental health nursing is a rich, distinctive and unique role with a special place in health systems. Mental health nurses engage in a wide variety of activities all aimed at helping each client. A new model of mental health nursing proposed aimed at giving a distinct identity.
(19) Harrison et al. (2017) Australia.	To explore how mental health nursing could be promoted as a sustainable career option.	Qualitative. Exploratory, cross‐sectional study.	150 mental health nurses from 1 public mental health service.	Stigma has a negative influence on perception of MHN as a career in Australia. Suggestions made to make it more attractive. Profile of MHN should be improved, MHN should take ownership of promoting MHN to students in university and reduce negative image. Better opportunities for MHN and specialist status should be awarded.
(20) Bray (1999) England.	To examine psychiatric nurse–patient relationships and aspects of professional closeness.	Qualitative. Ethnographic.	15 trained psychiatric nurses.	Psychiatric nurse experiences and attitudes of working closely with patients in a ward setting. Patient focus maintained despite interpersonal difficulties and difficult behaviour. Therapeutic relationship, helping patient feel accepted.
(21) Humble and Cross (2010) Australia.	To explore the experiences of veteran MHN and reasons for continuation in profession.	Qualitative. Heideggerian, Phenomenological Hermeneutic.	7 psychiatric nurses, 4 women and 3 men.	Psychiatric nurses express positive outlooks about their roles and identities.PN see themselves as different to other nurses, not intimidated, not easily shocked, common sense, curious, patient focused.
(22) White and Kudless (2008) United States.	To engage CMHNs in a dialogue about their role and any associated concerns.	Qualitative. Participatory Action Research.	36 CMHNs working in mental health services in southeast USA. 35 female, 1 male.	Struggle for an identity or voice, value of autonomy and the search for recognition. CMHNs not given credit for their work. Autonomy more important than salary to some. Other disciplines do not understand CMHN role. Systemic issues impede CMHN work.
(23) Hercelinskyj et al. (2014) Australia.	To identify ways to promote MHN in order to recruit and retain staff and promote MHN in universities.	Qualitative. Explorative Descriptive design.	11 MHN. 10 women and 1 man, working in mental health services in Victoria, Australia.	Challenges facing MHN role, challenges attracting next generation. Systemic difficulties make job hard. MHN must be promoted in the university. MHN status is eroded in the community due to being part of multi‐disciplinary team. Role ambiguity, MHN job not clear to people outside of MHN.

## 4 Findings

Following data analysis, five main themes emerged from the data: (1) Professional identity formation, (2) The attributes of mental health nurses, (3) The mental health nurse role, (4) The unique skills and knowledge of mental health nurses and (5) Professional identity and the future of mental health nursing.

### 4.1 Theme 1: Professional Identity Formation

The first theme focused on aspects of PI formation for mental health nurses, their reasons for choosing mental health nursing as a career and what factors may have positively or negatively influenced PI formation. Many participants made the choice to pursue a career in mental health or psychiatric nursing by contrasting it with general nursing and identifying key differences between both disciplines. Mental health nursing was viewed as attractive by some because it was seen as less technical and less restrictive than general nursing (Moir and Abraham 1996), or because mental health nursing was more patient‐focused than general nursing and uniforms were not worn (Sercu et al. 2015).

I enjoyed it (my mental health nursing placement) so much more than my general when I've been going through my training, and I really feel like I'm the kind of person who likes to sit and talk to patients. You have time in psyche. It's not so technical orientated. (Moir and Abraham 1996)

A formative step towards PI is the act of choosing mental health nursing as a career. The data gave a broad indication of the reasoning behind that choice for mental health nurses. Once a mental health nurse has chosen their career path by whatever means, the process of PI formation begins in earnest. Participants identified factors, such as their work with patients, and the learned roles and knowledge they gained from experienced staff during the socialisation process as key to their nursing identity formation. The role of education and training was also emphasised. (Hurley and Lakeman 2011).

The educational experience was not the same for everyone. Some mental health student nurses felt marginalised and made to feel different by university general nursing academic staff when they took a core nursing module with general nurses. General nursing academic staff reportedly lacked knowledge about mental health, and mental health nursing students were chastised for speaking up or having critical thinking ability. Thus, potentially leading to feelings of isolation among the participants and potentially having a negative impact on their PI.

We're seen as a bit quirky by general nurses. No‐one from mental health ever speaks up in lectures. The lecturer thought that a woman with schizophrenia should have their children taken of her, just because she has schizophrenia. That shows that there's an inadequate understanding of safeguarding. (Buescher and McGugan 2022)

The data on PI formation for mental health nurses suggested that people chose to be mental health nurses for many reasons, and they were socialised into their profession by exposure to clinical practice, role models and experience. Mental health nurses made a clear distinction between themselves and general nurses, perhaps the first step towards a PI.

### 4.2 Theme 2: The Personal Attributes of Mental Health Nurses

Many of the included studies suggested that mental health nurses perceived themselves as having attributes or characteristics that both predisposed them to a career in mental health nursing and were necessary for them to do their jobs effectively. These personal attributes were perceived by some as almost unique to mental health nursing and a key part of the PI. Mental health nurses in some studies viewed themselves as having attributes and attitudes, which they perceived to be different from other nursing disciplines. Pragmatism, flexibility, common sense, being down‐to‐earth, and being able to approach patients on an equal footing were commonly reported characteristics.

Community Psychiatric Nurses (CPNs) are patient, practical, flexible and with a good level of common sense. CPN is a translator on the patient's level. (Barlow 2006)

Participants in the studies conducted by Deady (2005) and Humble and Cross (2010) viewed themselves as different to other nursing disciplines, with mental health nurses in the latter study relishing the difference, seeing themselves as more liberal, less judgemental and with less susceptibility to being shocked than other branches of nursing.

I think you have to be able to accept a bit of a shock, or you wouldn't stay in the job, so that makes us a bit different because I've found a lot of nurses are very conservative. I think we are a different breed because we aren't shocked or horrified by what's at work every day (Elizabeth: Humble and Cross 2010)

Gender identity and societal views about the characteristics or attributes associated with gender were seen in some studies as influential on mental health nurse identity, arguably bordering on stereotype.

I think that society actually views women as being more sort of nurturing and caring and that it's a special type of male that actually comes into nursing in the first place. There are more males in psychiatry, and I think this is historical, taken from the old days of Victorian asylums. (Anthony: Holyoake 2002)

In reporting on the attitudes of Irish psychiatric nurses, Deady (2005) stated that participants believed that general nurses as well as society viewed psychiatric nursing as inferior because there are more men doing the job. Holyoake (2002) stated that at the time of writing of his study, 10% of the nursing workforce was male but 35% of management positions in nursing were taken by male nurses. Similarly, participants from the study by Crawford et al. (2008) seemed to dispute the idea that there are more male psychiatric nurses but still adhered to stereotypical views on gender in nursing, notably that women are less assertive than men and willing to tolerate more hardship, whereas men seek out nursing management roles because nurses' pay is too low (Crawford et al. 2008).

Based on this evidence, mental health nurses believed they possessed attributes which made them more suited to the role than other nurses. Such attributes and attitudes are not necessarily unique but contribute to their social identification. Conversely, gender‐based attributes are more likely to be imposed on nurses by society, but the nurses in the studies seemed to comply with them (Holyoake 2002).

### 4.3 Theme 3: The Mental Health Nurse Role

The role of the mental health nurse was mentioned in all 23 included studies in a variety of contexts. Role, in this case, refers to the practicalities of mental health nursing, notably, what it is that a mental health nurse actually does in the workplace. In many of the studies, the concept of the role of the mental health nurse and the concept of their PI was closely aligned.

Student mental health nurses in one study spoke about what they perceived as the role of qualified mental health nurses, based on their observations during their clinical placements. Among their perceptions of the mental health nurse role were that qualified mental health nurses engage in activities such as ward management, doing paperwork, advocating for patients and liaising on their behalf with other patients, giving handovers, attending ward rounds and administering medication (Rungapadiachy et al. 2004). Practical nursing duties such as medication administration, conducting domiciliary visits and giving injections were also identified as constituents of the mental health nurse role in other studies (Savio 1991; Barlow 2006; Karanikola et al. 2018; Hurley 2009). However, the mental health nurse role was described in much broader terms by a participant in the study by Hurley (2009).

The nurse, who is perhaps doing some sort of loose term psychological supportive therapy one moment, might the next moment be taking their pulse, or dressing a wound, and the next moment, might actually be helping them to sort their housing and the next moment possibly having a game of scrabble with that same person. Now I've never seen a psychotherapist, or, you know a clinical psychologist playing scrabble or fill in a housing form. (RP 24: Hurley 2009)

The everyday work of a mental health nurse is therefore quite varied, although the variation inherent in the mental health nurse role makes it appear ambiguous and not easy to define or describe. This ambiguity leads to lack of clarity about the mental health nurse's PI. Rasmussen et al. (2017) stated that PI is important as it gives mental health nurses a better understanding of their role, helps them identify a theoretical framework for their work and helps them determine which aspects of their role are specific to nursing. However, in a portion of the data, PI was difficult for mental health nurses to articulate.

Professional Identity? I find that quite difficult to answer. What my exact role is. I tend to say that I'm kind for a living. I help people to help themselves. I try to enable people, empower people. (RP 019: Crawford et al. 2008)

A key consequence of the multi‐faceted nature of the mental health nurse role was its invisibility, the fact that nobody sees the mental health nurse carrying out their most important duties. Or that important mental health nursing interventions, which might simply consist of talking to a patient, may or may not be perceived as legitimate work.

Sitting and talking with patients are not always activities that are clearly visible to others, on the face of it, it looks like they are just watching TV, but they are actually sitting and talking to somebody. (Participant 5: Rungapadiachy et al. 2004)

Working within a team was a common thread throughout the studies, participants accepted the value of a team environment but felt underestimated by the team.

Our contribution to the multidisciplinary team is not valued, we are not invited to the multidisciplinary perspective. Our voices are not included in the assessment and decision‐making process. (Rasmussen et al. 2017)

Some participants were more overtly resentful about their lack of power within their organisation, especially given their perceived importance to its running. Similarly, community mental health nurses in two studies felt undervalued and unrecognised despite their perception that they provide an invaluable service (White and Kudless 2008; Crawford et al. 2008). Similarly, mental health nurses encountered negative societal attitudes on mental health nursing but were nevertheless undeterred in their goal to progress in their careers.

I feel very passionate about nursing as a career. Nursing was never considered a respectable qualification but having done it, it's literally the best thing. (McCrae et al. 2014)

It is evident from the data that some mental health nurses perceived themselves as having low status or being undervalued. Barlow (2006) sought the opinion of non‐nursing members of a mental health team about the role of the Community Psychiatric Nurse (CPN), most of whom agreed that the CPN is highly valued, with excellent problem‐solving skills, a valuable knowledge base and an aptitude for critical thinking, despite the CPNs appearing to think the exact opposite. Barlow (2006) stated that the non‐nursing team members recognised the CPNs' input to the team more than the CPNs did. Crawford et al. (2008) described how participants in their study were self‐effacing in their attitude towards their achievements with patients leading to their work being invisible and them not receiving due credit. The concurrent desire for approval or recognition was described by participants in the same study.

So much of feeling good about your work and yourself when it comes down to it depends on whether other people appreciate it, like when the patients say thank you or if your manager does, if that was ever going to happen which seems pretty unlikely, but at the end of the day you're just so dependent on the approval of other people. (RP 31): Crawford et al. 2008)

### 4.4 Theme 4: The Unique Skills and Knowledge of Mental Health Nurses

Contrary to the idea of the day‐to‐day activity of mental health nursing being of low status or invisible, Santangelo et al. (2018) argued that mental health nursing consists of distinctive skills, knowledge and expertise and consequently, is specialist, with a broad scope and a high level of influence. Some of the data suggested some key factors which make a case for mental health nursing as unique compared to other nursing disciplines or professions.

Hurley (2009) stated that 79% of participants in his study said that mental health nurses spend more time with patients than other disciplines. Santangelo et al. (2018) stated that mental health nurses bring a new construct to the caring role, characterised by collaborative relationships which bring patient focus to a new level. The patient focus of mental health nurses was reflected very clearly in the data.

Everything we do here is to socialise the user, for that goal. Whether it is medication, a chat, a visit, manual activities, therapeutic follow up, any intervention, it is with the intention of improving their quality of life. (Joao: Reis et al. 2023)

Mental health nurses in the study by Bray (1999) where the research participants were engaged in close observation of difficult patients, remained highly motivated and patient focused, despite their work taking an emotional toll. Other participants in the same study highlighted the role they have in helping patients feel validated or accepted.

We're able to offer people a place where they feel safe. There's a chance to talk to people who care about what they're going through. Being there and being able to listen, they don't feel valued and that's what they need. (Bray 1999)

The therapeutic relationship was described as both a unique and definitive aspect of mental health nursing practice. Community mental health nurses viewed the formation of a therapeutic relationship with a patient and their family as one of the most important parts of their role.

The rapport you build with a patient is your main therapeutic tool (Nina). (Karanikola et al. 2018).

In contrast to the idea of mental health nurses having clear and specific specialist skills, the idea of them operating with a varied and unspecific set of skills was a common theme in the data. The term ‘Jack of All Trades’ appeared in some of the studies and reflected the diversity of the mental health nurse role but also its low professional status (Hurley 2009). However, it was seen by some mental health nurses as positive in some cases and a source of professional pride and as a negative my others

We are separate to other disciplines. We're not particularly intimidated or don't feel threatened constantly by people who are potentially aggressive. With medical nursing, where there's a physical problem, that's what they're there to do, they don't look at other issues. We've a greater range of skills (Robert). (Humble and Cross 2010)

I think that you are a ‘Jack of All Trades’, master of none in some respects, or you become very exceptional in the area in which you work. (Emma: Terry 2020)

Some of the data suggested that mental health nurses have constructed their PI by taking skills and knowledge from other healthcare disciplines. Terry (2020) suggested that mental health nursing might steal aspects of other healthcare roles and subsume them into their own identity. A participant felt that borrowing skills from other disciplines and repackaging them as mental health nursing is a special skill and worthy of recognition.

We have garnered many things from other professions to make up what we are. It's that bit about bringing many things together and it is how we put them together. There's no other profession that combines all these different things in the way mental health nursing does and then delivers it back. (RP8: Hurley 2009)

Similarly, mental health nurse participants did not think that they were in competition with other disciplines or could claim ownership of certain skills, rather they hold their own unique place within healthcare systems. Therefore, the idea that mental health nursing is made up of practices which other disciplines also practice, was not always viewed as negative (Santangelo et al. 2018).

### 4.5 Theme 5: Perceptions of Professional Identity and the Future of Mental Health Nursing

Considering the complexity of mental health nursing, as described by the nurses themselves, some of the data indicate how mental health nurses view their PI, and what they see as the status of their profession and how to improve their status. A portion of the data show how mental health nurses, particularly in Australia, feel about their identity after many years of generic nurse training.

I actually think that the nursing role has been eroded quite dramatically in the community to this generic title. Nurses are starting to lose what it is that makes them separate in their professional identity. (MHN no 4: Hercelinskyj et al. 2014)

Buescher and McGugan (2022) perhaps showed how mental health nursing education within a generic nursing curriculum might look, with minimal mental health content within core modules and mental health nursing students effectively excluded.

Mental health nursing is unpopular as a career choice for student nurses due to its weak PI, its poorly understood role, its invisibility, and the stigma associated with it (Harrison et al. 2017). The culmination of the unpopularity of mental health nursing is worsening recruitment and retention problems and the potential extinction of the profession. A participant in one study acknowledged that mental health nurses could do more to improve their standing.

I don't think we're very good at getting out there and promoting ourselves. I think we have to do that, otherwise we're just going to fizz away like the intellectual disability nurses. We're just going to fizzle out. I have almost a sense of urgency about it. (MHN 6: Hercelinskyj et al. 2014)

Another study found that unmotivated and negative mental health nursing profession was contributing to the recruitment of mental health nurses and that self‐promotion was imperative. The lack of specialist status was also seen as problematic for the future of mental health nursing in this study.

Mental health nursing should be made a proper specialty. It certainly isn't taught well on the wards. When the mental health nurses all retire there will be no specialist nurses left. (Harrison et al. 2017)

Wand et al. (2022) stated that the culture in which mental health nurses work must change with more emphasis on patient care, rewards for nurses who actively engage in professional development and less emphasis on excessive paperwork. Hercelinskyj et al. (2014) stated that mental health nurses experience role conflict when they are caught between keeping up with organisational demands, such as documentation, and their patient care role. This role conflict leads to an unclear PI. The whole picture of mental health nursing must be promoted if the profession is to continue.

We need to show students the potential areas psych nurses can work in, the opportunities that can be available, and define what we do with clients. It's not all about documentation, it's not all about medicating, it's the relational aspect of mental health nursing. (MHN 2: Hercelinskyj et al. 2014)

The future of nursing is therefore dependant on active promotion of the discipline by the current cohort of mental health nurses. Santangelo et al. (2018) suggested a reframing of what we already know about mental health nursing and proposed a substantive model of care. The aim of this new proposed model was to promote a broader appreciation of the distinctiveness and importance of mental health nursing, simply by creating an identity which was more understandable and easier to articulate to others. (Santangelo et al. 2018).

## 5 Discussion

This systematic review of mental health nurses' perceptions of their PI included qualitative data from 23 studies published between 1991 and 2022. The findings demonstrate that mental health nurses across different contexts, including specialist trained mental health nurses and generic trained nurses who specialise in mental health and work in mental health settings, possess a strong sense of identity with a clear understanding of the characteristics that make their role unique and distinct within the broader healthcare system.

However, their PI also appears to be shaped by negative societal attitudes and the inherently difficult‐to‐define nature of mental health nursing—factors which may, in turn, hinder their ability to feel fully confident in asserting the value and legitimacy of their role.

A prominent theme identified by participants across the studies included in this systematic review was the perceived distinctiveness of mental health nursing in contrast to general nursing, which in turn, served as a tangible aspect of PI. Participants in the studies by Moir and Abraham (1996) and Sercu et al. (2015) distinguished between the two disciplines based on factors such as the increased technical emphasis in general nursing leading to a higher level of psychomotor tasks, or a perceived reduced patient focus. Superficially, both disciplines are related by means of universal values such as compassion or professionalism (Lakeman 2012; Ohlen and Segesten 1998). However, Happell (1999) found that undergraduate nursing students in Australia held negative views about psychiatry, albeit without any prior experience, perceiving it to be dangerous and depressing.

Kerins et al. (2022) described how in medical education, social or group identity development, which occurs during professional socialisation, leads to the separation of medical specialist groups into factions or silos who are always in conflict and maintain an ‘us against them’ mentality. Therefore, perhaps in nursing, professional silos exist among the disciplines and characterise a part of group identity for each nursing discipline. Connell et al. (2022) argued that both mental health and general nursing have come from similar paternalistic, medical and empirical origins but mental health nursing has evolved into a unique entity with values, recovery and empowerment at its core, and therefore both specialities are clearly different. If this is the case, mental health nursing does not need to define itself by its contrasts with other disciplines and can justifiably stand alone, but the differences seem likely to remain a source of identity for some individuals.

As regards the personal characteristics which predispose some individuals to mental health nursing, Holyoake (2002) stated that gender‐based rules and norms remain a part of mental health nurse identity. Similarly, Torkelson and Seed (2011) found that male and female mental health nurses clearly operated within western structures of masculinity and femininity. In this study, women were observed to spend more time in the caring side of the mental health nurse role, building therapeutic relationships, whereas male nurses were more likely to engage in physical tasks. This was based on the idea that male nurses did not favour the relational side of mental health nursing (Torkelson and Seed 2011). There are likely to be mental health nurses who argue against gender stereotyping, but it remains a female‐dominated profession with more men taking up management roles (Gauci et al. 2023). In contrast to the idea of familiar ideas of gender roles contributing to mental health nurse identity, Shmilovitz et al. (2021) found that individuals with ‘androgynous’ sex types were more caring and had a higher ability for key aspects of mental health nursing than more traditional male or female archetypes. It can therefore be argued that gender in mental health nursing is irrelevant.

The varied and unclear mental health nurse role was a common theme among participants in the studies included in the present review. Role confusion or role overlap can lead to mental health nurses having trouble articulating their work and a strong PI (Terry 2020). Cowman et al. (2001) explored the role of Irish mental health nurses from which he identified nine categories of a mental health nurse's role, including patient assessment, care planning and pharmaceutical interventions. These categories could very easily be attributed to any other healthcare specialty and are not unique.

Barker and Buchanan‐Barker (2011) have stated that the main responsibility of mental health nurses remains keeping their patients and others safe, implementing medical interventions and managing their physical and social environment. Restrictive practices, such as chemical or physical restraint, remain a feature of mental health care (Bifarin et al. 2022). However, for the mental health nurse samples included in this systematic review, their job consisted of more than serving a basic function, with some identifying specialisms which they perceived to be distinctive to mental health nursing, such as the therapeutic relationship.

Critics of the therapeutic relationship have said that it is difficult to quantify and lacking a theoretical basis (Browne et al. 2012). Nevertheless, while a clear definition of the therapeutic relationship is elusive, it encapsulates many of the person‐centred aspects of mental health nursing such as empathy and genuineness (Browne et al. 2012). Perhaps what is also implied within the therapeutic relationship is the availability of time. Jackson and Stevenson (2000) stated that time is an essential component of mental health nursing given that most activities undertaken by mental health nurses require time. In other branches of nursing where psychomotor tasks are the dominant intervention, time might be viewed as a luxury. Connell et al. (2022) argued that the relationship between a mental health nurse and a patient, the connectedness, the advocacy, the collaboration, while not solely the remit of mental health nursing, is performed in a unique fashion by mental health nurses within the therapeutic relationship and is grossly undervalued by nursing authorities.

Some of the participants in the various studies featured in this systematic review acknowledged that the work of a mental health nurse includes duties which are also the remit of other professions. Barlow (2006) described how non‐nursing staff commented that the only thing that community psychiatric nurses did that they did not was to give injections. Mental health nursing occupies a liminal space characterised by vagueness and obscurity, taking on the roles of other healthcare professionals (Bifarin et al. 2022). Connell et al. (2022) stated that mental health nurses have adapted to meet the individual needs of patients, as well as helping to bolster a depleted allied healthcare workforce by bridging the gap between social work, psychology and psychiatry. While this in‐between position might cause role conflict for the nurses, Terry (2020) pointed out the importance of mental health nurses in the overall functioning of an organisation. Therefore, while the multifaceted nature of the mental health nursing role, often drawing from other disciplines, may risk diluting professional identity, this flexibility remains essential in ensuring the continuity and functionality of mental health services, particularly in situations where other disciplines are unavailable or absent.

Invisibility or a lack of professional recognition, despite a perception of their importance to the organisation, was mentioned by mental health nurses in some of the included studies (Crawford et al. 2008; White and Kudless 2008). Cleary et al. (2012) found that one‐third of a sample of mental health nurses working in an acute hospital setting stated that they rarely, or in some cases never, received any acknowledgement from senior management of their professional achievements. Elsewhere, Terry and Coffey (2019) described how mental health nurses on a psychiatric ward were perceived by patients to do little more than walk around the ward or sit in their office, whereas the nurses themselves insisted they were very busy with administrative duties. In this case, the casual and unqualified observation of a patient whether—accurate or not—constituted their individual interpretation of a mental health nurse's work.

The concept of invisibility and a lack of appreciation was also associated with mental health nurses' propensity to be self‐effacing (Rungapadiachy et al. 2004; Barlow 2006; Crawford et al. 2008). Therefore, because mental health nurses do not openly talk about their work or look for recognition, they do not receive it. There was some evidence from the study by Barlow (2006) that within their teams, mental health nurses are valued but perhaps their value to others is not explicitly shared with them. However, due to the resentful tone of some of the qualitative data, it could be interpreted as a dichotomy between nurses who wanted approval that never materialised and nurses who perhaps relished not receiving approval as it strengthened their feeling of being badly treated, which in turn could be adopted as part of their identity.

It is perhaps unfair to speculate that mental health nurses derive their identity from feelings of injustice at being undervalued or poorly treated. Many of the nurses who contributed to the studies included in this systematic review carried out their work with enthusiasm (Humble and Cross 2010). Some had negative feelings but only due to institutional constraints which made it difficult for them to do their job effectively (Terry 2020). Current research indicates that mental health nurses are proud and happy with their chosen career (Hercelinskyj 2022). Cleary et al. (2005) found that 69% of mental health nurse respondents in a quantitative study would choose to do mental health nursing if they could go back to when they started nursing. In the same study, 68% of the respondents felt proud to be a mental health nurse and 71% said that they would recommend mental health nursing to others (Cleary et al. 2005). The evidence therefore suggests that the current mental health nurse workforce is satisfied and enthusiastic about their work.

The position of mental health nurses in the future is perhaps less certain, especially considering that the lack of a PI is a key reason why the recruitment and retention of the next generation of mental health nurses may be challenging (Karlowicz and Ternus 2009; Hercelinskyj 2022). A portion of the literature on the future of mental health nursing, against the backdrop of the adoption of a generic qualification, presents a concerning scenario with mental health nurses effectively eradicated and speculatively replaced with care staff (Stickley et al. 2009; Lakeman and Molloy 2018).

It is hard to find any evidence in the literature, particularly from the extensively documented Australian experience, to suggest that the adoption of a generic nursing model has led to improvements in mental health care or yielded positive outcomes for service users or the professional standing of mental health nursing (Hemingway et al. 2016; Lakeman and Molloy 2018; Haslam 2023). McIntosh (2017) stated that in New Zealand, which has had a similar transition to generic nursing, UK‐trained mental health nurses have been highly sought after due to their specialist knowledge. It is unclear whether specialist mental health nurses from overseas are equally sought after in Australia, but the example from New Zealand offers some evidence that specialist nurses are still important to mental health services, or that mental health services cannot function without them.

Many of the mental health nurses included in this review, who expressed opinions about the future of mental health nursing, were working within a system in which generic nurse education was well established. They had a clear idea about what needed to be done to prevent the demise of the profession, beginning with promotion of mental health nursing as a viable career option at educational institutions (Harrison et al. 2017; Hercelinskyj et al. 2014). The UK should adopt higher standards for mental health nursing education with less emphasis on the biomedical approaches and more focus on the interpersonal patient‐centred skills that are synonymous with the discipline (Haslam 2023; Hurley et al. 2024; Wand 2024).

Likewise, some researchers such as Warrender et al. (2024) hold firm that mental health nursing is unique, with a distinct identity and it is in fact psychiatry that is vague and subjective. Connell et al. (2022) argued that mental health nursing is more than the empirical world of psychiatry; it is a philosophy, unique and even if it is hard to describe and undervalued, it is important. In moving away from a strictly biomedical model and embracing the aspects of mental health nursing that are held dear by practitioners, as highlighted in this review, the essence of mental health nurse professional identity may be found.

## 6 Conclusion

Based on the subjective views of mental health nurses over a 33‐year timeframe, the results of this systematic review provide some confirmation of what critics and researchers have previously identified. Mental health nurses' professional identity is complex and hard to clearly define but not beyond comprehension. It is perhaps more easily understood through its interpersonal focus and not through comparison with other disciplines who is PI may be more clearly defined. However, given the positive attitudes of the mental health nurses featured in this SR about their work, it is hard to envisage mental health nurses standing by and allowing their profession to diminish.

### 6.1 Implications for Practice

PI in mental health nursing has been well researched, as has the status of mental health nursing in the context of future generic nurse education in the UK. However, the collective views of mental health nurses themselves have been less documented and arguably present a more hopeful picture of the future of mental health nursing. Future research on the subject should consider the in‐depth opinion of practising mental health nurses to obtain a more accurate picture.

## Author Contributions

The first and third reviewers collaborated on the study concept and protocol. The first reviewer independently conducted systematic searches, critical appraisal, data extraction and analysis. All three reviewers contributed to screening and study selection. The manuscript was written by the first reviewer, with supervision by the third reviewer and review by the second and third reviewers.

## Ethics Statement

The authors have nothing to report.

## Conflicts of Interest

The authors declare no conflicts of interest.

## Supporting information


**Data S1:** inm70137‐sup‐0001‐DataS1.docx.

**Data S2:** inm70137‐sup‐0002‐DataS2.docx.

**Data S3:** inm70137‐sup‐0003‐DataS3.docx.

**Data S4:** inm70137‐sup‐0004‐DataS4.docx.

**Data S5:** inm70137‐sup‐0005‐DataS5.docx.

## Data Availability

The data that support the findings of this study are available from the corresponding author upon reasonable request.
