# Supplementary material for: A Systematic Review of Mental Health Nurses' Perceptions of Their Professional Identity
Source: Int J Ment Health Nurs. 2025 Sep 24;34(5):e70137. doi: 10.1111/inm.70137 (PMC12459084; doi:10.1111/inm.70137)
Supplement: Supplementary file 1 — Data S1: inm70137‐sup‐0001‐DataS1.docx. [file INM-34-0-s005.docx]

**Appendix 1: Tables of Search Words as Used in Each Database.**

**1: Cinahl Ultimate Search Terms.**

| Mental Health Nurse. | AND | Professional Identity. | AND | Mental Health Nurse Role | AND | Perceptions. |
| --- | --- | --- | --- | --- | --- | --- |
| MH “Psychiatric Nursing+” OR  MH “community mental health nursing” OR  MH “community mental health nurses” OR  “Mental Health Nursing” OR  “Psychiatric Nursing” OR  “Psych Nursing” OR  “Mental Health Nurses” OR  “Psychiatric Nurses” OR  “Psych Nurses” |  | MH ”Professional Identity” OR  MH “Social Identity+” OR  MH “Identity Crisis” OR  MH Professionalism OR  “Social Identification” OR  “Nursing Identity” OR“Self-Concept”. |  | MH Role+ OR  MH “Role Conflict” OR  MH “Professional Role+” OR  MH “Gender Role+” OR  “Nurse’s Role” OR  “Role Ambiguity” OR  “Role Stress” OR  “Role Confusion”. |  | MH Perception+ OR  Views OR  Opinions OR  Thoughts OR  Experiences Or  Attitudes OR  Perceptions OR Beliefs. |

**2: EBSCO Medline Search Terms.**

| Mental Health Nurse. | AND | Professional Identity. | AND | Mental Health Nurse Role | AND | Perceptions. |
| --- | --- | --- | --- | --- | --- | --- |
| MH “Psychiatric Nursing” OR  “Mental Health Nursing” OR “Psych Nursing” OR  “Community Mental Health Nursing” OR  “Psychiatric Nursing” OR  “Mental Health Nurses” OR  “Psychiatric Nurses” OR  “Psych Nurses” OR “community mental health nurses”. |  | MH “Social Identification” OR MH Professionalism OR MH “Identity Crisis” OR ”Professional Identity” OR  “Nursing Identity” OR  “Self-Concept”. |  | MH “Professional Role OR MH “Role+” OR  MH “Role Conflict” OR  ” OR  MH “Gender Role+” OR MH “Nurse’s Role” OR  “Role Ambiguity” OR  “Role Stress” OR  “Role Confusion”. |  | MH “Perception+” OR  Views OR  Opinions OR  Thoughts OR  Experiences Or  Attitudes OR  Perceptions OR Beliefs. |

**3: EBSCO APA PsycINFO and APA PsycARTICLES Search Terms.**

| Mental Health Nurse. | AND | Professional Identity. | AND | Mental Health Nurse Role | AND | Perceptions. |
| --- | --- | --- | --- | --- | --- | --- |
| “Mental Health Nursing” OR “Psychiatric Nursing” OR “Psych Nursing” OR “Mental Health Nurses” OR “Psychiatric Nurses” OR “Psych Nurses” OR “Mental Health Nurse” OR “Psychiatric Nurse” OR “Psych Nurse” OR “Community Mental Health Nursing” OR “Community Mental Health Nurses.” |  | DE “Professional Identity” OR DE “Professionalism” OR “Social Identification” OR “Social Identity” OR “Nursing Identity” OR “Identity Crisis” OR “Self-Concept2. |  | DE “Professional Role” OR DE “Role Conflicts” OR DE “Roles” OR “Role Ambiguity” OR “Role Stress” OR “Role Confusion” 0R “Nurse’s Role”. |  | DE “Attitudes” OR  Views OR  Opinions OR  Thoughts OR  Experiences Or  Perceptions OR Beliefs. |

**4: PubMed search terms.**

| Mental Health Nurse. | AND | Professional Identity. | AND | Mental Health Nurse Role | AND | Perceptions. |
| --- | --- | --- | --- | --- | --- | --- |
| “Psychiatric Nursing”[Mesh]  OR  “Mental Health Nursing” OR “Psych Nursing” OR  “Community Mental Health Nursing” OR  “Psychiatric Nursing” OR  “Mental Health Nurses” OR  “Psychiatric Nurses” OR  “Psych Nurses” OR “Community Mental Health Nurses”. |  | ”Social Identification”[Mesh]  OR Professionalism[Mesh]  OR “Identity Crisis”[Mesh]  OR “Professional Identity” OR  “Social Identity”  Or  “Nursing Identity”  Or  “Self-concept.” |  | “Nurse’s Role”[Mesh]  OR “Professional Role”[Mesh] OR  Role[Mesh]  OR  “Role Conflict”[Mesh]  Or  “Gender Role”  Or  “Role Ambiguity”  Or  “Role Stress”  Or “Role Confusion” |  | Perception[Mesh]  OR Views OR  Opinions OR  Thoughts OR  Experiences Or  Attitudes OR  Perceptions OR Beliefs. |

**5: Scopus Search Terms.**

| Mental Health Nurse. | AND | Professional Identity. | AND | Mental Health Nurse Role | AND | Perceptions. |
| --- | --- | --- | --- | --- | --- | --- |
| “mental health nursing” OR “psychiatric nursing” OR “psych nursing” OR “community mental health nursing” OR “mental health nurses” OR “psychiatric nurses” OR “psych nurses” or “community mental health nurses.” |  | “Professional identity” or “social identification” or “social identity” or “nursing identity” or “identity crisis” or “self-concept” or “professionalism.” |  | “role” or “nurse’s role” or “professional role” or “gender role” or “role ambiguity” or “role stress” or “role conflict” or “role confusion.” |  | “Perception” or “views” or “opinions” or “thoughts” or “experiences” or “attitudes” or “perceptions” or “beliefs.” |

**CINAHL Ultimate Search String 29/04/2024. Title or Abstract Search.**

| **Search.** | **Search Terms** | **Number of Hits.** |
| --- | --- | --- |
| #1 | MH “Psychiatric Nursing+” or MH “Community Mental Health Nursing” or MH “Community Mental Health Nurses” | 24,409. |
| #2 | MH “Professional Identity” or MH “Social Identity+” or MH “Identity Crisis” or MH “Professionalism” | 22,529. |
| #3 | MH “Role+” or MH “Role Conflict” or MH “Professional Role+” or MH “Role Stress” or MH “Gender Role+” | 166,088. |
| #4 | MH “Perception+” | 95,438. |
| #5 | MH “Qualitative Studies+” | 194,118. |
| #6 | TI “Mental Health Nursing” or “Psych Nursing” or “Mental Health Nurses” or “Psychiatric Nurses” or “Psych Nurses” or AB “Mental Health Nursing” or “Psych Nursing” or “Mental Health Nurses” or “Psychiatric Nurses” or “Psych Nurses” | 15,059. |
| #7 | #1 or #6. | 31,304 |
| #8 | TI “Social Identification” or “Nursing Identity” or “Self-concept” or “Nurse’s Role” or “Role Ambiguity” or “Role Confusion” or AB “Social Identification” or “Nursing Identity” or “Self-concept” or “Nurse’s Role” or “Role Ambiguity” or “Role Confusion” | 6957. |
| #9 | #2 or #3 or #8. | 188,529. |
| #10 | Views or Opinions or Thoughts or Experiences or Attitudes or Perceptions or Beliefs or AB Views or Opinions or Thoughts or Experiences or Attitudes or Perceptions or Beliefs. | 789908. |
| #11 | #4 or #10. | 849,455. |
| #12 | #5 and #7 and #9 and #11 | 327 |
|  |  |  |
|  |  |  |
|  | Total: | 327 |

**CINAHL Ultimate Search String 29/04/2024. All Fields.**

| **Search.** | **Search Terms** | **Number of Hits.** |
| --- | --- | --- |
| #1 | MH “Psychiatric Nursing+” or MH “Community Mental Health Nursing” or MH “Community Mental Health Nurses” | 24,408. |
| #2 | MH “Professional Identity” or MH “Social Identity+” or MH “Identity Crisis” or MH “Professionalism” | 22,528. |
| #3 | MH “Role+” or MH “Role Conflict” or MH “Professional Role+” or MH “Role Stress” or MH “Gender Role+” | 166,072. |
| #4 | MH “Perception+” | 95,432. |
| #5 | MH “Qualitative Studies+” | 194,109 |
| #6 | “Mental Health Nursing” or “Psych Nursing” or “Mental Health Nurses” or “Psychiatric Nurses” or “Psych Nurses” | 21,673 |
| #7 | #1 or #6. | 27,681 |
| #8 | “Social Identification” or “Nursing Identity” or “Self-concept” or “Nurse’s Role” or “Role Ambiguity” or “Role Confusion” | 76,020 |
| #9 | #2 or #3 or #8. | 220,881 |
| #10 | Views or Opinions or Thoughts or Experiences or Attitudes or perceptions or beliefs | 1071784 |
| #11 | #4 or #10. | 1,094,667 |
| #12 | #5 and #7 and #9 and #11 | 389 |
|  |  |  |
|  |  |  |
|  | Total: | 389 |

**EBSCO Medline Search String 21/4/24. All Fields.**

| Search. | Search terms. | No of Hits. |
| --- | --- | --- |
| #1 | MH “Psychiatric Nursing” | 18,324. |
| #2 | MH “Social Identification” or MH “Professionalism” or MH “Professional Role+” or MH “Identity Crisis”. | 102,008. |
| #3 | MH “Role +” or MH “Nurse’s Role” or MH “Role Conflict”. | 114,124. |
| #4 | #2 or #3. | 126,213. |
| #5 | MH “Perception+” | 486,830. |
| #6 | MH “Qualitative Research+” | 86,922. |
| #7 | Mental Health Nursing or Mental Health Nurse or Psychiatric Nurse or Psych Nurse or Mental Health Nurses or Psychiatric Nurses or Psych Nurses or Community Mental Health Nurses or Community Mental Health Nurse. | 33,605. |
| #8 | #1 or #7. | 36,402. |
| #9 | Professional Identity or Nursing Identity or Self-concept or Role Ambiguity or Role Stress or Role Confusion. | 127,177. |
| #10 | #4 or #9. | 262,838. |
| #11 | Views or Opinions or Thoughts or Experiences or Attitudes or Perceptions or Beliefs. | 2,863,244. |
| #12 | #5 or #11. | 3,042,421. |
| #13 | Qualitative. | 370,725. |
| #14 | #6 or #13. | 371,075 |
| #15 | #8 and #10 #and #12 and #14. | 412. |
|  | Total. | 412. |

**EBSCO Medline Search String 29/4/24. All Text.**

| Search. | Search terms. | No of Hits. |
| --- | --- | --- |
| #1 | MH “Psychiatric Nursing” | 18,336 |
| #2 | MH “Social Identification” or MH “Professionalism” or MH “Professional Role+” or MH “Identity Crisis”. | 102,045. |
| #3 | MH “Role +” or MH “Gender Role” or MH “Nurse’s Role” or MH “Role Conflict”. | 114,433. |
| #4 | MH “Perception+” | 487,138. |
| #5 | MH “Qualitative Research”. | 87,200 |
| #6 | TX “Mental Health Nursing” or “Psych Nursing” or Community Mental Health Nursing” or “Mental Health Nurses” or Psychiatric Nurses” or “Psych Nurses” or “Community Mental Health Nurses” | 14,234. |
| #7 | #1 OR #7 | 28,199. |
| #8 | TX “Professional Identity” or “Nursing Identity” or “Self-concept” or “Role Ambiguity” or “Role Stress” or “Role Confusion”. | 70,692. |
| #9 | #2 or #3 or #8 | 191,534. |
| #10 | TX Views or Opinions or Thoughts or Experiences or Attitudes or Perceptions or Beliefs. | 3,043,499 |
| #11 | #4 or #12. | 3,220,647. |
| #12 | TX | 251. |
|  |  |  |
|  |  |  |
|  |  |  |
|  | Total. | 251. |

**APA PsycINFO search string 27^th^ April 2024. All fields.**

| Search. | Search Terms. | No of Hits. |
| --- | --- | --- |
| #1 | mental health nursing or psychiatric nursing or psych nursing or mental health nurses or psychiatric nurses or psych nurses or mental health nurse or psychiatric nurse or psych nurse or community mental health nursing or community mental health nurses. | 20,615. |
| #2 | DE "Professional Identity" OR DE "Professional Role" OR DE "Professionalism" | 11,818. |
| #3 | DE "Role Conflicts" OR DE "Roles" | 12,069. |
| #4 | social identification or social identity or nursing identity or identity crisis or self-concept or role or nurse's role or role ambiguity or role stress or role confusion | 801,139. |
| #5 | #2 or #3 or #4 | 806,711. |
| #6 | DE "Attitudes" | 26,401. |
| #7 | views or opinions or thoughts or experiences or perceptions or beliefs | 1,422,109. |
| #8 | #6 or #7 | 1,431,147. |
| #9 | DE “Qualitative Methods” | 11,517. |
| #10 | Qualitative | 237,091. |
| #11 | #9 or #11. | 237,091. |
| #12 | #1 and #5 and #8 and #11 | 666. |
|  | Total studies | 666. |
|  |  |  |

**APA PsycINFO search string 29^th^ April 2024. Title and Abstract.**

| Search. | Search Terms. | No of Hits. |
| --- | --- | --- |
| #1 | TI “Mental Health Nursing” or “Psychiatric Nursing” or “Psych Nursing” or “Community Mental Health Nursing” or “Mental Health Nurses” or “Psychiatric Nurses” or “Psych Nurses” or “Community Mental Health Nurses” or AB “Mental Health Nursing” or “Psychiatric Nursing” or “Psych Nursing” or “Community Mental Health Nursing” or “Mental Health Nurses” or “Psychiatric Nurses” or “Psych Nurses” or “Community Mental Health Nurses” | 9,572. |
| #2 | DE "Professional Identity" OR DE "Professional Role" OR DE "Professionalism" | 11,818. |
| #3 | DE "Role Conflicts" OR DE "Roles" OR DE “Gender Roles” | 12,560 |
| #4 | TI “Social Identification” or “Social Identity” or “Nursing Identity” or “Identity Crisis” or “Self-concept” or “Role” or “Nurse's Role” or “Role Ambiguity” or “Role Stress” or “Role Confusion” or AB “Social Identification” or “Social Identity” or “Nursing Identity” or “Identity Crisis” or “Self-concept” or “Role” or “Nurse's Role” or “Role Ambiguity” or “Role Stress” or “Role Confusion” | 618,320 |
| #5 | #2 or #3 or #4 | 630,215 |
| #6 | DE "Attitudes" | 26,401. |
| #7 | TI ( Views or Opinions or Thoughts or Experiences or Perceptions or Beliefs ) OR AB ( Views or Opinions or Thoughts or Experiences or Perceptions or Beliefs ) | 1,180,969 |
| #8 | #6 or #7 | 1,191,622 |
| #9 | DE “Qualitative Methods” | 11,517. |
| #10 | TI Qualitative or AB Qualitative | 217,573 |
| #11 | #9 or #10 | 219,233 |
| #12 | #1 and #5 and #8 and #11 | 235 |
|  | Total studies | 235 |
|  |  |  |
|  |  |  |

**APA PsycINFO search string 29^th^ April 2024. All fields.**

| Search. | Search Terms. | No of Hits. |
| --- | --- | --- |
| #1 | “Mental Health Nursing” or “Psychiatric Nursing” or “Psych Nursing” or “Community Mental Health Nursing” or “Mental Health Nurses” or “Psychiatric Nurses” or “Psych Nurses” or “Community Mental Health Nurses” | 16,743. |
| #2 | DE "Professional Identity" OR DE "Professional Role" OR DE "Professionalism" | 11,818. |
| #3 | DE "Role Conflicts" OR DE "Roles" OR DE “Gender Roles” | 12,560. |
| #4 | “Social Identification” or “Social Identity” or “Nursing Identity” or “Identity Crisis” or “Self-concept” or “Role” or “Nurse's Role” or “Role Ambiguity” or “Role Stress” or “Role Confusion” | 710,601. |
| #5 | #2 or #3 or #4 | 718,211. |
| #6 | DE "Attitudes" | 26,401. |
| #7 | views or opinions or thoughts or experiences or perceptions or beliefs | 1,422,109 |
| #8 | #6 or #7 | 1,431,147. |
| #9 | DE “Qualitative Methods” | 11,517. |
| #10 | qualitative | 237,091 |
| #11 | #9 and #11 | 237,091 |
| #12 | #1 and #5 and #8 and #11 | 543. |
|  |  |  |

**CINAHL Ultimate Search String 30/04/2024. Title or Abstract Search.**

| **Search.** | **Search Terms** | **Number of Hits.** |
| --- | --- | --- |
| #1 | MH “Psychiatric Nursing+” or MH “Community Mental Health Nursing” or MH “Community Mental Health Nurses” | 24,412 |
| #2 | MH “Professional Identity” or MH “Social Identity+” or MH “Identity Crisis” or MH “Professionalism” | 22,541. |
| #3 | MH “Role+” or MH “Role Conflict” or MH “Professional Role+” or MH “Role Stress” or MH “Gender Role+” | 166,112. |
| #4 | MH “Perception+” | 95,455 |
| #5 | MH “Qualitative Studies+” | 194,202. |
| #6 | TI “Mental Health Nursing” or “Psych Nursing” or “Mental Health Nurses” or “Psychiatric Nurses” or “Psych Nurses” or AB “Mental Health Nursing” or “Psych Nursing” or “Mental Health Nurses” or “Psychiatric Nurses” or “Psych Nurses” | 8241. |
| #7 | #1 or #6. | 27,160 |
| #8 | TI “Social Identification” or “Nursing Identity” or “Self-concept” or “Nurse’s Role” or “Role Ambiguity” or “Role Confusion” or AB “Social Identification” or “Nursing Identity” or “Self-concept” or “Nurse’s Role” or “Role Ambiguity” or “Role Confusion” | 6959. |
| #9 | #2 or #3 or #8. | 188,559. |
| #10 | TI Views or Opinions or Thoughts or Experiences or Attitudes or Perceptions or Beliefs or AB Views or Opinions or Thoughts or Experiences or Attitudes or Perceptions or Beliefs. | 790219. |
| #11 | #4 or #10. | 849,780. |
| #12 | TI Qualitative OR AB Qualitative | 182,261 |
| #13 | #5 or #12 | 270,033 |
|  | #7 and #9 and #11 and #13 |  |
|  | Total: | 308 |

**EBSCO Medline Search String 29/4/24. All Text.**

| Search. | Search terms. | No of Hits. |
| --- | --- | --- |
| #1 | MH “Psychiatric Nursing” | 18,336 |
| #2 | MH “Social Identification” or MH “Professionalism” or MH “Professional Role+” or MH “Identity Crisis”. | 102,051. |
| #3 | MH “Role +” or MH “Gender Role” or MH “Nurse’s Role” or MH “Role Conflict”. | 114,435. |
| #4 | MH “Perception+” | 487,164. |
| #5 | MH “Qualitative Research”. | 87,232. |
| #6 | TX “Mental Health Nursing” or “Psych Nursing” or Community Mental Health Nursing” or “Mental Health Nurses” or Psychiatric Nurses” or “Psych Nurses” or “Community Mental Health Nurses” | 14,006. |
| #7 | #1 OR #7 | 28,041. |
| #8 | TX “Professional Identity” or “Nursing Identity” or “social identity” or “Self-concept” or “Role Ambiguity” or “Role Stress” or “Role Confusion”. | 73,898. |
| #9 | #2 or #3 or #8 | 193,818 |
| #10 | TX Views or Opinions or Thoughts or Experiences or Attitudes or Perceptions or Beliefs. | 3,043,682. |
| #11 | #4 or #12. | 3,220,842. |
| #12 | TX Qualitative | 420,483. |
| #13 | #5 or #12 | 420,750. |
| #14 | #7 and #9 and #11 and #13 | 561. |
|  |  |  |
|  | Total. | 561. |

**SCOPUS search string 27^th^ April 2024.**

“mental health nursing*” or “psychiatric nursing*” or “community mental health nursing*” and “professional identity” or “social identification” or “social identity” or “nursing identity” or “identity crisis” or “self-concept” or “professionalism” or “role” or “nurse’s role” or “professional role” or “role ambiguity” or “role stress” or “role conflict” or “role confusion” and “perception” or “views” or “opinions” or “thoughts” or “experiences” or “attitudes” or “perceptions” or “beliefs” and “qualitative studies”

135 results.

**SCOPUS search string 30^th^ April.**

“mental health nursing*” or “psychiatric nursing*” or “community mental health nursing*” and “professional identity” or “social identification” or “social identity” or “nursing identity” or “identity crisis” or “self-concept” or “professionalism” or “role” or “nurse’s role” or “professional role” or “gender role” or “role ambiguity” or “role stress” or “role conflict” or “role confusion” and “perception” or “views” or “opinions” or “thoughts” or “experiences” or “attitudes” or “perceptions” or “beliefs” and “qualitative studies” or “qualitative” 536.

**PubMed Search String 27/04/24.**

| Search. | Search terms. | No of Hits. |
| --- | --- | --- |
| #1 | “Psychiatric Nursing”[Mesh] | 18,334. |
| #2 | “Social Identification”[Mesh] | 10,263. |
| #3 | “Role”[Mesh] or “Nurse’s Role”[Mesh] or “Professional Role”[Mesh] or “Role Conflict”[Mesh] | 114,177. |
| #4 | “Perception”[Mesh] | 487,277. |
| #5 | “Qualitative Research”[Mesh] | 87,280. |
| #6 | “Mental Health Nursing” or “Psych Nursing” or “Mental Health Nurses” or Psychiatric Nurses” or “Psych Nurses” or Mental Health Nurse” or “Psychiatric Nurse” or “Psych Nurse” or “Community Mental Health Nursing” or “Community Mental Health Nurses” | 83,205. |
| #7 | #1 or #6 | 83,205. |
| #8 | “Professional Identity” or “Social Identity” or “Nursing Identity” or “Identity Crisis” or “Professionalism” or “Self-concept” or “Role Ambiguity” or “Role Stress” or “Role Confusion” | 82,316. |
| #9 | #2 or #3 or #8 | 198,962. |
| #10 | Views or Opinions or Thoughts or Experiences or Attitudes or Perceptions or Beliefs | 4,115,643. |
| #11 | #4 or #10 | 4,115,643. |
| #12 | #5 and #7 and #9 and #11 | 399. |
|  | Total studies. | 399. |

**PubMed Search String 30/04/24. All Fields.**

| Search. | Search terms. | No of Hits. |
| --- | --- | --- |
| #1 | “Psychiatric Nursing”[Mesh] | 18,336. |
| #2 | “Social Identification”[Mesh] | 10,269. |
| #3 | “Role”[Mesh] or “Nurse’s Role”[Mesh] or “Professional Role”[Mesh] or “Role Conflict”[Mesh] | 114,473. |
| #4 | “Professionalism”[Mesh} | 1898. |
| #5 | “Perception”[Mesh] | 487,430. |
| #6 | “Qualitative Research”[Mesh] | 87,433. |
| #7 | “Mental Health Nursing” or “Psych Nursing” or “Community Mental Health Nursing” or “Mental Health Nurses” or “Psychiatric Nurses” or “Psych Nurses” or Community Mental Health Nurses” | 8,040. |
| #8 | #1 or #6 | 22,216. |
| #9 | “Professional Identity” or “Social Identity” or “Nursing Identity” or “Identity Crisis” or “Self-concept” or “Role Ambiguity” or “Role Stress” or “Role Confusion” | 73,400 |
| #10 | #2 or #3 or #4 or #9 | 190,912 |
| #11 | Views or Opinions or Thoughts or Experiences or Attitudes or Perceptions or Beliefs | 4,117,798. |
| #12 | #5 or #11 | 4,117,798 |
| #13 | Qualitative. | 425,237. |
| #14 | #6 or #13 | 425,559. |
| #15 | #8 or #10 or #12 or #14 | 266. |

**PubMed Search String 01/05/24. Title/Abstract.**

| Search. | Search terms. | No of Hits. |
| --- | --- | --- |
| #1 | “Psychiatric Nursing”[Mesh] | 18,336. |
| #2 | “Social Identification”[Mesh] | 10,269. |
| #3 | “Role”[Mesh] or “Nurse’s Role”[Mesh] or “Professional Role”[Mesh] or “Role Conflict”[Mesh] or “Gender Role”[Mesh] | 114,477. |
| #4 | “Professionalism”[Mesh} | 1897. |
| #5 | “Perception”[Mesh] | 487,463. |
| #6 | “Qualitative Research”[Mesh] | 87,477. |
| #7 | **"Mental Health Nursing"[Title/Abstract] OR "Psych Nursing"[Title/Abstract] OR "Community Mental Health Nursing"[Title/Abstract] OR "Mental Health Nurses"[Title/Abstract] OR "Psychiatric Nurses"[Title/Abstract] OR "Psych Nurses"[Title/Abstract] OR "Community Mental Health Nurses"[Title/Abstract]** | 6,356. |
| #8 | #1 or #6 | 20,621. |
| #9 | "Professional Identity"[Title/Abstract] OR "Social Identity"[Title/Abstract] OR "Nursing Identity"[Title/Abstract] OR "Identity Crisis"[Title/Abstract] OR "Self-concept"[Title/Abstract] OR "Role Ambiguity"[Title/Abstract] OR "Role Stress"[Title/Abstract] OR "Role Confusion"[Title/Abstract] | 13,896. |
| #10 | #2 or #3 or #4 or #10 | 137,171. |
| #11 | "Views"[Title/Abstract] OR "Opinions"[Title/Abstract] OR "Thoughts"[Title/Abstract] OR "Experiences"[Title/Abstract] OR "Attitudes"[Title/Abstract] OR "Perceptions"[Title/Abstract] OR "Beliefs"[Title/Abstract] | 720,826. |
| #12 | #5 or #12 | 1,169,843 |
| #13 | Qualitative. | 351,920. |
| #14 | #6 or #13 | 370,479. |
| #15 | #8 or #10 or #12 or #14 | 137. |

**PubMed Search String 01/05/24. Title/Abstract.**

| Search. | Search terms. | No of Hits. |
| --- | --- | --- |
| #1 | “Psychiatric Nursing”[Mesh] | 18,336. |
| #2 | “Social Identification”[Mesh] | 10,269. |
| #3 | “Role”[Mesh] or “Nurse’s Role”[Mesh] or “Professional Role”[Mesh] or “Role Conflict”[Mesh] | 114,476. |
| #4 | “Professionalism”[Mesh} | 1897. |
| #5 | “Perception”[Mesh] | 487,463. |
| #6 | “Qualitative Research”[Mesh] | 87,465. |
| #7 | **"Mental Health Nursing"[Title/Abstract] OR "Psych Nursing"[Title/Abstract] OR "Community Mental Health Nursing"[Title/Abstract] OR "Mental Health Nurses"[Title/Abstract] OR "Psychiatric Nurses"[Title/Abstract] OR "Psych Nurses"[Title/Abstract] OR "Community Mental Health Nurses"[Title/Abstract]** | 6,356. |
| #8 | #1 or #6 | 20,621. |
| #9 | "Professional Identity"[Title/Abstract] OR "Social Identity"[Title/Abstract] OR "Nursing Identity"[Title/Abstract] OR "Identity Crisis"[Title/Abstract] OR "Self-concept"[Title/Abstract] OR "Role Ambiguity"[Title/Abstract] OR "Role Stress"[Title/Abstract] OR "Role Confusion"[Title/Abstract] | 13,896. |
| #10 | #2 or #3 or #4 or #10 | 137,172. |
| #11 | "Views"[Title/Abstract] OR "Opinions"[Title/Abstract] OR "Thoughts"[Title/Abstract] OR "Experiences"[Title/Abstract] OR "Attitudes"[Title/Abstract] OR "Perceptions"[Title/Abstract] OR "Beliefs"[Title/Abstract] | 720,838. |
| #12 | #5 or #12 | 1,169,867. |
| #13 | Qualitative. | 351,934. |
| #14 | #6 or #13 | 370,495. |
| #15 | #8 or #10 or #12 or #14 | 137. |

**APA PsycARTICLES 06/05/2024**

| Search. | Search Terms. | No of Hits. |
| --- | --- | --- |
| #1 | TI “Mental Health Nursing” or “Psychiatric Nursing” or “Psych Nursing” or “Community Mental Health Nursing” or “Mental Health Nurses” or “Psychiatric Nurses” or “Psych Nurses” or “Community Mental Health Nurses” or AB “Mental Health Nursing” or “Psychiatric Nursing” or “Psych Nursing” or “Community Mental Health Nursing” or “Mental Health Nurses” or “Psychiatric Nurses” or “Psych Nurses” or “Community Mental Health Nurses” | 43 |
| #2 | DE "Professional Identity" OR DE "Professional Role" OR DE "Professionalism" | 388 |
| #3 | DE "Role Conflicts" OR DE "Roles" OR DE “Gender Roles” | 1001 |
| #4 | TI “Social Identification” or “Social Identity” or “Nursing Identity” or “Identity Crisis” or “Self-concept” or “Role” or “Nurse's Role” or “Role Ambiguity” or “Role Stress” or “Role Confusion” or AB “Social Identification” or “Social Identity” or “Nursing Identity” or “Identity Crisis” or “Self-concept” or “Role” or “Nurse's Role” or “Role Ambiguity” or “Role Stress” or “Role Confusion” | 26,358. |
| #5 | #2 or #3 or #4 | 26.862. |
| #6 | DE "Attitudes" | 22218. |
| #7 | TI Views or Opinions or Thoughts or Experiences or Perceptions or Beliefs or AB Views or Opinions or Thoughts or Experiences or Perceptions or Beliefs | 60,247. |
| #8 | #6 or #7 | 61,230. |
| #9 | DE “Qualitative Methods” | 454 |
| #10 | TI Qualitative or AB Qualitative | 4729 |
| #11 | #9 or #10 | 4789 |
| #12 | #1 and #5 and #8 and #11 | 0 |
|  | Total studies | 0 |
|  |  |  |

**PubMed 6^th^ may 24.**

| Search. | Search terms. | No of Hits. |
| --- | --- | --- |
| #1 | “Psychiatric Nursing”[Mesh] | 18,337. |
| #2 | “Social Identification”[Mesh] | 10,275. |
| #3 | “Role”[Mesh] or “Nurse’s Role”[Mesh] or “Professional Role”[Mesh] or “Role Conflict”[Mesh] or “Gender Role”[Mesh] | 114,496. |
| #4 | “Professionalism”[Mesh} | 1897. |
| #5 | “Perception”[Mesh] | 487,672. |
| #6 | “Qualitative Research”[Mesh] | 87,600. |
| #7 | **"Mental Health Nursing"[Title/Abstract] OR "Psych Nursing"[Title/Abstract] OR "Community Mental Health Nursing"[Title/Abstract] OR "Mental Health Nurses"[Title/Abstract] OR "Psychiatric Nurses"[Title/Abstract] OR "Psych Nurses"[Title/Abstract] OR "Community Mental Health Nurses"[Title/Abstract]** | 6,360 |
| #8 | #1 or #7 | 20,626. |
| #9 | "Professional Identity"[Title/Abstract] OR "Social Identity"[Title/Abstract] OR "Nursing Identity"[Title/Abstract] OR "Identity Crisis"[Title/Abstract] OR "Self-concept"[Title/Abstract] OR "Role Ambiguity"[Title/Abstract] OR "Role Stress"[Title/Abstract] OR "Role Confusion"[Title/Abstract] | 13,901. |
| #10 | #2 or #3 or #4 or #9 | 137,199. |
| #11 | "Views"[Title/Abstract] OR "Opinions"[Title/Abstract] OR "Thoughts"[Title/Abstract] OR "Experiences"[Title/Abstract] OR "Attitudes"[Title/Abstract] OR "Perceptions"[Title/Abstract] OR "Beliefs"[Title/Abstract] | 721,442. |
| #12 | #5 or #11 | 1,170,652. |
| #13 | Qualitative[Title/Abstract] | 352,309 |
| #14 | #6 or #13 | 370,880. |
| #15 | #8 or #10 or #12 or #14 | 137. |

**CINAHL Ultimate Search String 11/05/2024. Title or Abstract Search.**

| **Search.** | **Search Terms** | **Number of Hits.** |
| --- | --- | --- |
| #1 | MH “Psychiatric Nursing+” or MH “Community Mental Health Nursing” or MH “Community Mental Health Nurses” | 24,551 |
| #2 | MH “Professional Identity” or MH “Social Identity+” or MH “Identity Crisis” or MH “Professionalism” | 22,628. |
| #3 | MH “Role+” or MH “Role Conflict” or MH “Professional Role+” or MH “Role Stress” or MH “Gender Role+” | 166,593. |
| #4 | MH “Perception+” | 95,682. |
| #5 | TI “Mental Health Nursing” or “Psych Nursing” or “Mental Health Nurses” or “Psychiatric Nurses” or “Psych Nurses” or AB “Mental Health Nursing” or “Psych Nursing” or “Mental Health Nurses” or “Psychiatric Nurses” or “Psych Nurses” | 8274. |
| #6 | #1 or #5 | 27,310 |
| #7 | TI “Social Identification” or “Nursing Identity” or “Self-concept” or “Nurse’s Role” or “Role Ambiguity” or “Role Confusion” or AB “Social Identification” or “Nursing Identity” or “Self-concept” or “Nurse’s Role” or “Role Ambiguity” or “Role Confusion” | 6978. |
| #8 | #2 or #3 or #7. | 189,114 |
| #9 | TI Views or Opinions or Thoughts or Experiences or Attitudes or Perceptions or Beliefs or AB Views or Opinions or Thoughts or Experiences or Attitudes or Perceptions or Beliefs. | 793,326. |
| #10 | #4 or #9. | 853,000 |
| #11 | #6 and #8 and #10 | 800 |
|  | Total: | 800 |
|  |  |  |

E**BSCO Medline Search String 11/5/24. All Text.**

| Search. | Search terms. | No of Hits. |
| --- | --- | --- |
| #1 | MH “Psychiatric Nursing” | 18,348 |
| #2 | MH “Social Identification” or MH “Professionalism” or MH “Professional Role+” or MH “Identity Crisis”. | 102,162 |
| #3 | MH “Role +” or MH “Gender Role” or MH “Nurse’s Role” or MH “Role Conflict”. | 114,525. |
| #4 | MH “Perception+” | 487,886. |
| #5 | TX “Mental Health Nursing” or “Psych Nursing” or Community Mental Health Nursing” or “Mental Health Nurses” or Psychiatric Nurses” or “Psych Nurses” or “Community Mental Health Nurses” | 14,081. |
| #6 | #1 OR #5 | 28,125 |
| #7 | TX “Professional Identity” or “Nursing Identity” or “social identity” or “Self-concept” or “Role Ambiguity” or “Role Stress” or “Role Confusion”. | 74,032. |
| #8 | #2 or #3 or #7 | 194,052. |
| #9 | TX Views or Opinions or Thoughts or Experiences or Attitudes or Perceptions or Beliefs. | 3,051,988. |
| #10 | #4 or #9 | 3,229,412. |
| #1 | #6 or #8 or #10 | 1711. |
|  |  |  |
|  | Total. | 1711. |

**APA PsycINFO search string 11^th^ may 2024. Title and Abstract.**

| Search. | Search Terms. | No of Hits. |
| --- | --- | --- |
| #1 | TI “Mental Health Nursing” or “Psychiatric Nursing” or “Psych Nursing” or “Community Mental Health Nursing” or “Mental Health Nurses” or “Psychiatric Nurses” or “Psych Nurses” or “Community Mental Health Nurses” or AB “Mental Health Nursing” or “Psychiatric Nursing” or “Psych Nursing” or “Community Mental Health Nursing” or “Mental Health Nurses” or “Psychiatric Nurses” or “Psych Nurses” or “Community Mental Health Nurses” | 6,462. |
| #2 | DE "Professional Identity" OR DE "Professional Role" OR DE "Professionalism" | 12,308. |
| #3 | DE "Role Conflicts" OR DE "Roles" OR DE “Gender Roles” | 15,889. |
| #4 | TI “Social Identification” or “Social Identity” or “Nursing Identity” or “Identity Crisis” or “Self-concept” or “Role” or “Nurse's Role” or “Role Ambiguity” or “Role Stress” or “Role Confusion” or AB “Social Identification” or “Social Identity” or “Nursing Identity” or “Identity Crisis” or “Self-concept” or “Role” or “Nurse's Role” or “Role Ambiguity” or “Role Stress” or “Role Confusion” | 700,645. |
| #5 | #2 or #3 or #4 | 713,753. |
| #6 | DE "Attitudes" | 32,326. |
| #7 | TI ( Views or Opinions or Thoughts or Experiences or Perceptions or Beliefs ) OR AB ( Views or Opinions or Thoughts or Experiences or Perceptions or Beliefs ) | 1,370,046. |
| #8 | #6 or #7 | 1,383,935. |
| #9 | #1 and #5 and #8 | 578 |
|  | Total studies | 578. |
|  |  |  |
|  |  |  |

**APA PsycARTICLES 11/05/2024 Title and Abstract Search.**

| Search. | Search Terms. | No of Hits. |
| --- | --- | --- |
| #1 | TI “Mental Health Nursing” or “Psychiatric Nursing” or “Psych Nursing” or “Community Mental Health Nursing” or “Mental Health Nurses” or “Psychiatric Nurses” or “Psych Nurses” or “Community Mental Health Nurses” or AB “Mental Health Nursing” or “Psychiatric Nursing” or “Psych Nursing” or “Community Mental Health Nursing” or “Mental Health Nurses” or “Psychiatric Nurses” or “Psych Nurses” or “Community Mental Health Nurses” | 43 |
| #2 | DE "Professional Identity" OR DE "Professional Role" OR DE "Professionalism" | 388 |
| #3 | DE "Role Conflicts" OR DE "Roles" OR DE “Gender Roles” | 1001 |
| #4 | TI “Social Identification” or “Social Identity” or “Nursing Identity” or “Identity Crisis” or “Self-concept” or “Role” or “Nurse's Role” or “Role Ambiguity” or “Role Stress” or “Role Confusion” or AB “Social Identification” or “Social Identity” or “Nursing Identity” or “Identity Crisis” or “Self-concept” or “Role” or “Nurse's Role” or “Role Ambiguity” or “Role Stress” or “Role Confusion” | 26,373. |
| #5 | #2 or #3 or #4 | 26.887. |
| #6 | DE "Attitudes" | 2,220 |
| #7 | TI Views or Opinions or Thoughts or Experiences or Perceptions or Beliefs or AB Views or Opinions or Thoughts or Experiences or Perceptions or Beliefs | 60,275. |
| #8 | #6 or #7 | 61,258. |
| #9 | #1 and #5 and #8 | 3 |
|  | Total studies | 3 |
|  |  |  |

SCOPUS 11^th^ may 2024.

“mental health nursing*” or “psychiatric nursing*” or “community mental health nursing*” and “professional identity” or “social identification” or “social identity” or “nursing identity” or “identity crisis” or “self-concept” or “professionalism” or “role” or “nurse’s role” or “professional role” or “gender role” or “role ambiguity” or “role stress” or “role conflict” or “role confusion” and “perception” or “views” or “opinions” or “thoughts” or “experiences” or “attitudes” or “perceptions” or “beliefs”

2831 without limiters

**PubMed 11^th^ may 24. Title and Abstract Search.**

| Search. | Search terms. | No of Hits. |
| --- | --- | --- |
| #1 | “Psychiatric Nursing”[Mesh] | 18,347. |
| #2 | “Social Identification”[Mesh] | 10,280. |
| #3 | “Role”[Mesh] or “Nurse’s Role”[Mesh] or “Professional Role”[Mesh] or “Role Conflict”[Mesh] or “Gender Role”[Mesh] | 114,535. |
| #4 | “Professionalism”[Mesh} | 1901. |
| #5 | “Perception”[Mesh] | 487,916. |
| #6 | **"Mental Health Nursing"[Title/Abstract] OR "Psych Nursing"[Title/Abstract] OR "Community Mental Health Nursing"[Title/Abstract] OR "Mental Health Nurses"[Title/Abstract] OR "Psychiatric Nurses"[Title/Abstract] OR "Psych Nurses"[Title/Abstract] OR "Community Mental Health Nurses"[Title/Abstract]** | 6,363. |
| #7 | #1 or #6 | 20,636. |
| #8 | "Professional Identity"[Title/Abstract] OR "Social Identity"[Title/Abstract] OR "Nursing Identity"[Title/Abstract] OR "Identity Crisis"[Title/Abstract] OR "Self-concept"[Title/Abstract] OR "Role Ambiguity"[Title/Abstract] OR "Role Stress"[Title/Abstract] OR "Role Confusion"[Title/Abstract] | 13,919. |
| #9 | #2 or #3 or #4 or #8 | 137,262. |
| #10 | "Views"[Title/Abstract] OR "Opinions"[Title/Abstract] OR "Thoughts"[Title/Abstract] OR "Experiences"[Title/Abstract] OR "Attitudes"[Title/Abstract] OR "Perceptions"[Title/Abstract] OR "Beliefs"[Title/Abstract] | 722,306. |
| #11 | #5 or #10 | 1,171,725. |
| #12 | #7 or #9 or #11 | 452 |

**SCOPUS 12^th^ May 2024.**

| Search. | Search Terms. | No of Hits. |
| --- | --- | --- |
| #1 | “mental health nursing” OR “psychiatric nursing” OR “psych nursing” OR “community mental health nursing” OR “mental health nurses” OR “psychiatric nurses” OR “psych nurses” or “community mental health nurses” | 23,671. |
| #2 | “professional identity” or “social identification” or “social identity” or “nursing identity” or “identity crisis” or “self-concept” or “professionalism” or “role” or “nurse’s role” or “professional role” or “gender role” or “role ambiguity” or “role stress” or “role conflict” or “role confusion” | 7000,285. |
| #3 | “perception” or “views” or “opinions” or “thoughts” or “experiences” or “attitudes” or “perceptions” or “beliefs” | 7090,289. |
| #4 | #1 AND #2 AND #3. | 3238. |
| LIMITERS APPLIED. | English, articles, qualitative research | 358 |
|  | total | 358 |
|  |  |  |

( TITLE-ABS-KEY ( "mental health nursing" OR "psychiatric nursing" OR "psych nursing" OR "community mental health nursing" OR "mental health nurses" OR "psychiatric nurses" OR "psych nurses" OR "community mental health nurses" ) ) AND ( TITLE-ABS-KEY ( "professional identity" OR "social identification" OR "social identity" OR "nursing identity" OR "identity crisis" OR "self-concept" OR "professionalism" OR "role" OR "nurse’s role" OR "professional role" OR "gender role" OR "role ambiguity" OR "role stress" OR "role conflict" OR "role confusion" ) ) AND ( TITLE-ABS-KEY ( "perception" OR "views" OR "opinions" OR "thoughts" OR "experiences" OR "attitudes" OR "perceptions" OR "beliefs" ) ) AND ( LIMIT-TO ( DOCTYPE , "ar" ) ) AND ( LIMIT-TO ( LANGUAGE , "English" ) ) AND ( LIMIT-TO ( EXACTKEYWORD , "Qualitative Research" ) )

**SCOPUS 14^th^ May 2024.**

| Search. | Search Terms. | No of Hits. |
| --- | --- | --- |
| #1 | “mental health nursing” OR “psychiatric nursing” OR “psych nursing” OR “community mental health nursing” OR “mental health nurses” OR “psychiatric nurses” OR “psych nurses” or “community mental health nurses” | 23,674. |
| #2 | “professional identity” or “social identification” or “social identity” or “nursing identity” or “identity crisis” or “self-concept” or “professionalism” or “role” or “nurse’s role” or “professional role” or “gender role” or “role ambiguity” or “role stress” or “role conflict” or “role confusion” | 7003,471. |
| #3 | “perception” or “views” or “opinions” or “thoughts” or “experiences” or “attitudes” or “perceptions” or “beliefs” | 7093,287. |
| #4 | #1 AND #2 AND #3. | 3238. |
| LIMITERS APPLIED. | English, articles, qualitative research | 358 |
|  | total | 358 |
