# Supplementary figures and images for: A Systematic Review of Mental Health Nurses' Perceptions of Their Professional Identity
Source: Int J Ment Health Nurs. 2025 Sep 24;34(5):e70137. doi: 10.1111/inm.70137 (PMC12459084; doi:10.1111/inm.70137)

**Supplementary Information 2: Sample CASP Tool.**

**
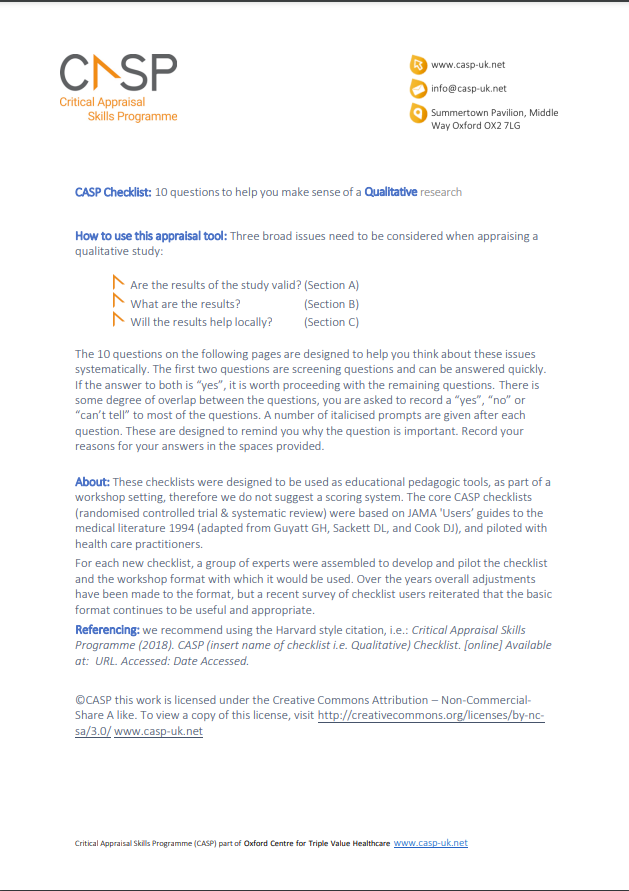
**

**
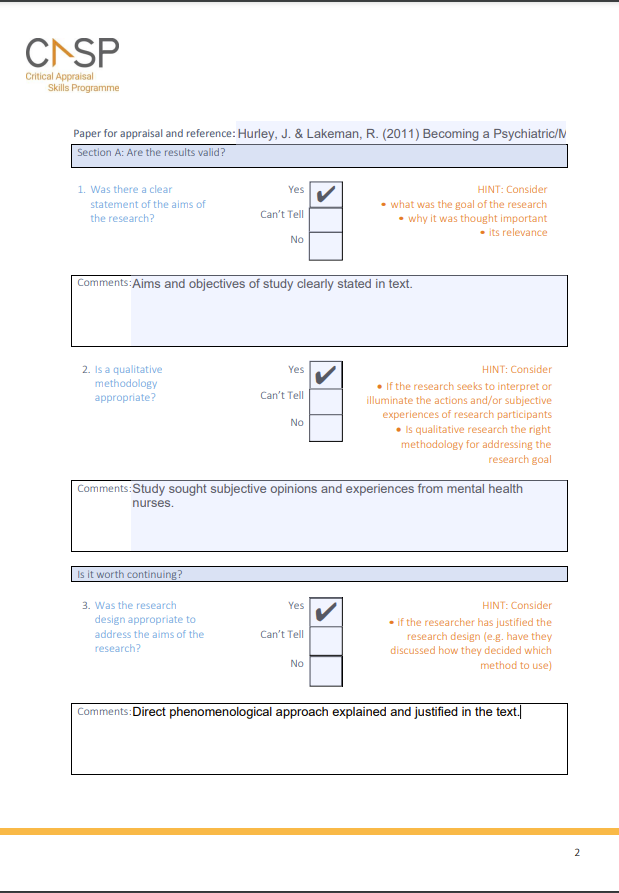
**

**
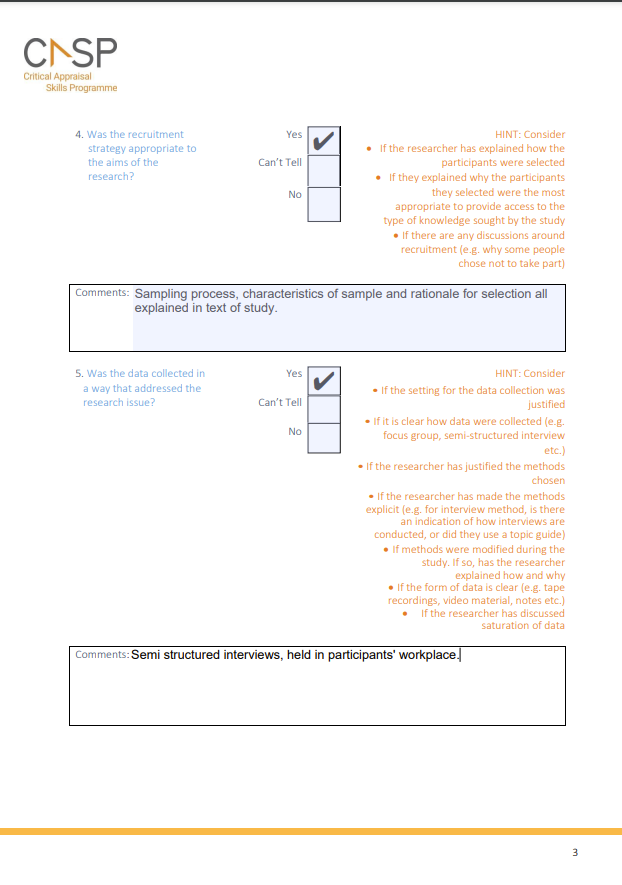
**

**
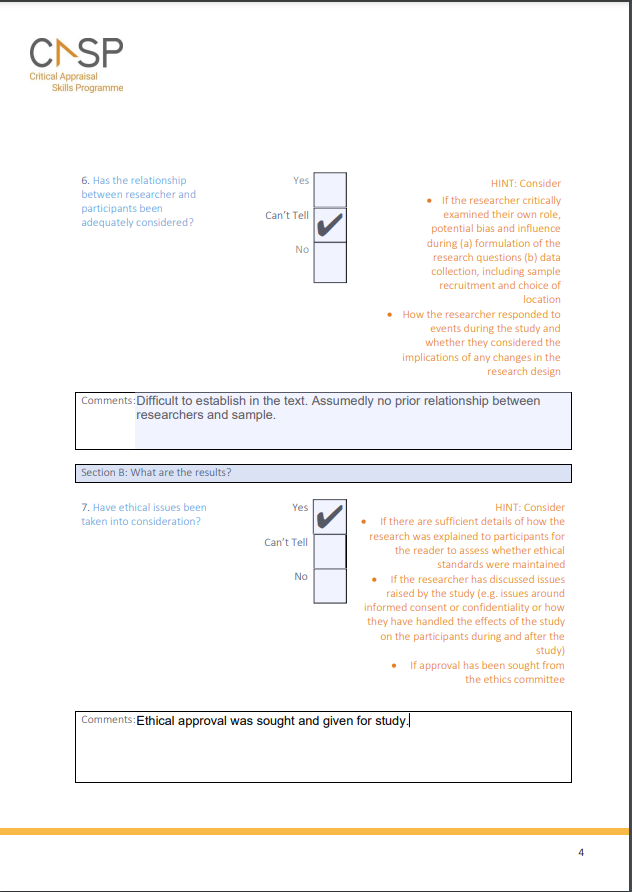
**

**
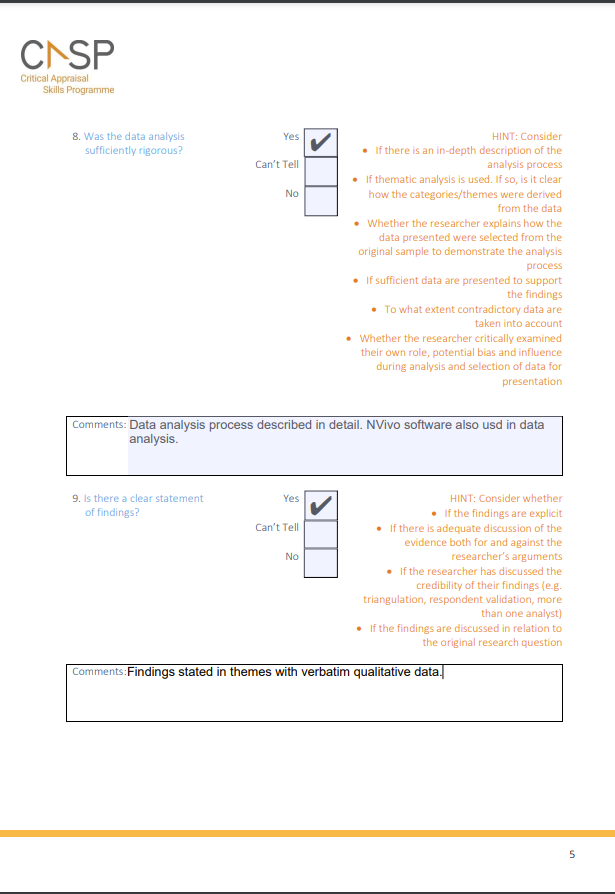
**

**
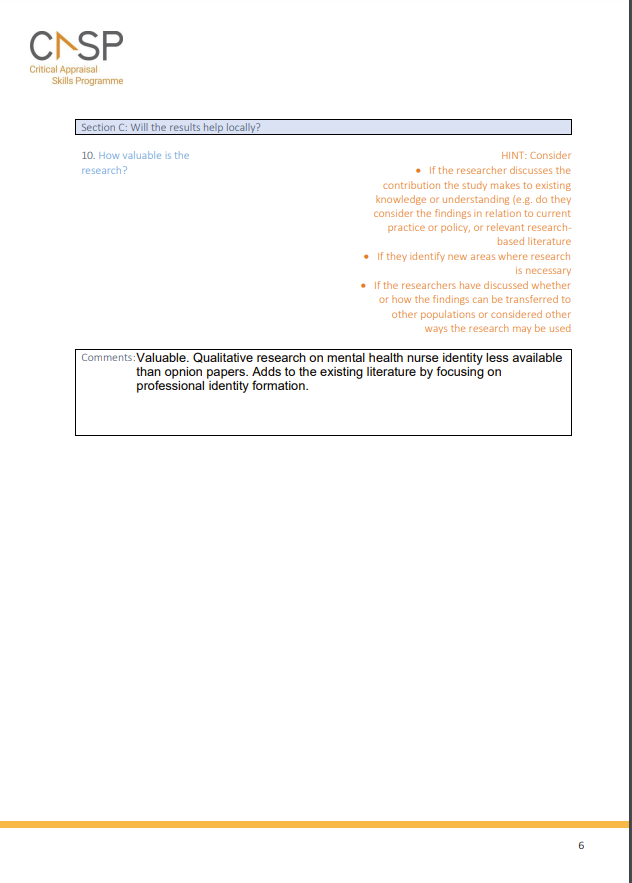
**

Supplement: Supplementary file 2 — Data S2: inm70137‐sup‐0002‐DataS2.docx. [file INM-34-0-s003.docx]
