# Supplementary material for: A Systematic Review of Mental Health Nurses' Perceptions of Their Professional Identity
Source: Int J Ment Health Nurs. 2025 Sep 24;34(5):e70137. doi: 10.1111/inm.70137 (PMC12459084; doi:10.1111/inm.70137)
Supplement: Supplementary file 3 — Data S3: inm70137‐sup‐0003‐DataS3.docx. [file INM-34-0-s004.docx]

**Data Extraction 1.**

| Study Reference: Rungapadiachy D.M., Madill A. & Gough B. (2004) Mental Health Student Nurses’ Perception of the Role of the Mental Health Nurse. *Journal of Psychiatric and Mental Health Nursing.* **11**, 714-724. | | |
| --- | --- | --- |
| Person extracting data: Donagh O’Brien. | Date data extracted: 20/05/2024. | |
| Aim of Study: To investigate the role of mental health nurses from the point of view of student mental health nurses. | | |
| Study design: Qualitative, Grounded Theory Approach. | | |
| Description of study setting/location(s): Study took place in the school of nursing in an unnamed University in UK, the name was withheld to maintain confidentiality, | Study period (dates study was done):  Study conducted during participants’ last 6 months of transition to qualified mental health nurse. | |
| Sampling method (e.g. purposive, convenient, etc.): Grounded Theory saturation approach used. | | |
| Inclusion and Exclusion criteria: Undergraduate mental health nursing students. | | |
| Final numbers included in the study: 14 student mental health nurses. | | |
| Description of the recruited sample (e.g. demographics, etc.) 14 MHSNs, all undertaking 3-year diploma in nursing studies. 8 women and 6 men. Age between 21 and 50 with a mean age of 35. | | |
| Description of data collection method (e.g. type of interviews, observations, location of data collection, etc.)  Data collection through semi-structured interviews. Interviews were structured around themes which were identified by first researcher.  Interviews took 45 mins to 1 hour and were conducted during last 6 months of MHSN transition to qualification. Interviews conducted in small classroom and tape recorded. Tape recordings transcribed verbatim. | | |
| Description of data analysis method (e.g. content analysis, thematic, etc.): Thematic analysis. QSR NUD*ST 4 software used. | | |
| **Findings:** All data that reflects views or experiences of the phenomenon under investigation | | Location/ page in text |
| 6 themes highlighted in results.  **Theme 1: Mental health nurse as administrator.**  Mental health nurses seen by student mental health nurses as having an administrative role.  Managing day to day activities. Doing lots of paperwork. Liaison between patient and other staff. Participating in ward rounds. Giving handovers. Advocating between patient and team.  “Nurses constantly manage the patients’ day-to-day neds and requirements.”  “The amount of paperwork made it so the nurses didn’t have a lot of one-to-one contact with patients.”  “There’s a tendency to hide behind paperwork.”  Student nurses did recognise nurses as advocates.  “Sometimes when a patient had an adverse reaction to medication, it was usually the nurse who informs the doctor and suggests that the patient’s medication be reviewed.”  **Theme 2: MHN as an agent of physical interventions.**  MHSN observe MHN engaging in hands on activities or “clinical duties” or non-hands on care/”non-clinical duties.”  Clinical duties consist of helping patient with activities of daily living such as nutrition or hygiene. Also consists of “Level obs”, or general observation of patient.  These are also jobs assigned to care attendants.  Non-clinical duties consist of activities such as bed making, laundry or escorting patients.  “MHN has to practically do everything for dementia patients, feeding them making sure they are ok, etc.”  **Theme 3: MHN as administrator of drugs.**  MHSN observed MHN as drug administrator. This is seen as an important role. Some MHNS feel that medication is overused. Some feel that there is not enough emphasis on other kinds of therapies.  “Too much emphasis on medication and little emphasis on communicating with people.”  **Theme 4: MHN as agent of psychological interventions.**  MHNS observed that engaging in therapeutic activities such as groups or one to one was very important. Sitting with a patient for a chat was seen as very important but also invisible because it is generally unstructured. Sometimes nurses too busy to engage in therapeutic groups.  *“Sitting and talking with patients are not always activities that are clearly visible to others, on the face of it, it looks like they are just watching TV, but they are actually sitting and talking to somebody.”*  **Theme 5: MHN as teacher. MHNS saw MHN as having a formal role as** teacher to care attendants, families etc.  *“MHN would explain things to families or reading rights to patients.”*  **Theme 6: MHN as agent of non-therapeutic interventions.**  MHNS observed/perceived MHN as engaging in non-therapeutic activities such as misconduct or malpractice. Others were observed to be deliberately not involved with patients, such as gossiping in the nurses’ station. Others seemed to lack key skills. Others had a negative attitude or approach to care.  *“Some nurses would hide when things got tense.”* | | **716-717.**  **717-718.**  **718.**  **718.**  *718.*  **718.**  **718-719.**  *719* |

**Data Extraction 2.**

| Study Reference: Barlow K. (2006) Perceptions of the Role of the Community Psychiatric Nurse. *Nursing Times.* 102 (**9**), 34-38. | | |
| --- | --- | --- |
| Person extracting data: Donagh O’Brien. | Date data extracted: 20/05/2024. | |
| Aim of Study: To explore the self-perceived differences between the contributions of CPN compared with other MDT members in a community mental health team for older adults. To examine the role of the CPN in dementia care on a CMHT. | | |
| Study design: Qualitative. | | |
| Description of study setting/location(s): Community mental health team (CMHT) for older persons in Dumfries & Galloway, Scotland. | Study period (dates study was done): Not stated in text. | |
| Sampling method (e.g. purposive, convenient, etc.): Convenient sample. Participants recruited anonymously at a multi-disciplinary meeting at the researcher’s workplace. | | |
| Inclusion and Exclusion criteria: Community Psychiatric Nurses (CPN) and staff who work alongside them. | | |
| Final numbers included in the study: 9 participants in total. | | |
| Description of the recruited sample (e.g. demographics, etc.) 4 CPNs and 5 other members of staff from researcher’s team. Disciplines not stated. All members had backgrounds of working in older adult or dementia care. | | |
| Description of data collection method (e.g. type of interviews, observations, location of data collection, etc.) Questionnaires containing a number of open ended questions, which would have constituted the basis of a semi structured interview, were distributed to staff at a team meeting. | | |
| Description of data analysis method (e.g. content analysis, thematic, etc.): Thematic analysis. | | |
| **Findings:** All data that reflects views or experiences of the phenomenon under investigation | | Location/ page in text |
| **8 themes in accordance with questions asked to participants.**  **1: Skills and Attributes.**  CPNs feel that “they utilise models of care such as holism and humanism.”  CPNs believe that “they translate a traditional medical model approach to working with people with dementia into a more humanistic and holistic approach.”  *“Community Psychiatric Nurses (CPNs) are patient, practical, flexible and with a good level of common sense. CPN is a translator on the patient’s level.”*  CPN uses therapeutic relationship.  Other staff see CPN as “having knowledge, giving advice, problem solving, education.”  **2: Knowledge base**  CPN found it hard to identify knowledge base which informs their practice. They could identify acquired practitioner knowledge.  CPNs identify sources of knowledge such as books. They see the value of life experience etc.  Others saw CPN as knowledgeable about medical conditions, medication, giving injections.  **3: Teamworking.**  CPNs felt they brought *“pragmatism, flexibility, versatility, and multiskilling to teamwork. Utilitarian role, filling in for absent colleagues.”*  Therapeutic relationships, relationship forming and relationship using.  Others saw CPN as someone who questions, has good communication skills, valued team advisor. Team recognised these skills before CPN did.  Skill sharer.  **4: Value of Contributors.**  CPNs had differing views on how they were viewed by other team members.  Some thought they were *“taken for granted, some thought they were highly valued, some thought they were valued depending on their contribution.”*  CPN thinks they are valued for their feedback at team meetings but not for the knowledge or skill which were used to gain the information.  **5: Differences in care provision.**  Most CPNs thought they brought something different to the team and that their unique attributes were valuable. They felt like they were a good resource for the team.  Others viewed CPN as doing more visits but without clear reason.  CPN knowledge base is limited to medication and mental illness.  But problem-solving skills and therapeutic activities recognised.  **6: Practice Example**  CPN finds it difficult to remain loyal to the team if team is making decisions which have negative connotations for patient.  **7: Training**  CPN thought that they had limited access to post grad training.  Most of their skills were learned on the job.  **8: Perceived Consensus.**  *“Lack of understanding and consensus about CPN role.”*  Some CPNs “*didn’t understand their own role.”*  *“Same kinds of tasks as other team members.”*  *“The only thing that CPNs do that we don’t is jab bums.”* | | **36.**  36.  **36.**  **36**  **37.**  **37.**  37-38.  38.  38.  38. |

**Data Extraction 3.**

| Study Reference: Deady R. (2005) Psychiatric Nursing in Ireland: A Phenomenological Study of the Attitudes, Values, and Beliefs of Irish Trained Psychiatric Nurses. *Archives of Psychiatric Nursing.* 19 (**5**), 210-216. | | |
| --- | --- | --- |
| Person extracting data: Donagh O’Brien. | Date data extracted: 21/05/2024. | |
| Aim of Study: To ascertain whether there is a collective set of attitudes, values, and beliefs which underpin the experience of Irish psychiatric nurses. | | |
| Study design: Qualitative. Phenomenological Approach. | | |
| Description of study setting/location(s): Public mental health service in Ireland, providing inpatient and outpatient care. | Study period (dates study was done):  Not stated in text. | |
| Sampling method (e.g. purposive, convenient, etc.): Purposive sample. Chosen due to their level of experience, training, and their representative grade within nurse management. All participants were taught under Irish apprenticeship method. | | |
| Inclusion and Exclusion criteria: Irish ethnicity, more than 10 years’ experience, working within representative grade of clinical or nurse management. | | |
| Final numbers included in the study: 8 participants. | | |
| Description of the recruited sample (e.g. demographics, etc.) 4 men and 4 women, Irish born and educated, have worked within clinical or nursing management. | | |
| Description of data collection method (e.g. type of interviews, observations, location of data collection, etc.) Structured interviews. | | |
| Description of data analysis method (e.g. content analysis, thematic, etc.): Thematic Analysis. | | |
| **Findings:** All data that reflects views or experiences of the phenomenon under investigation | | Location/ page in text |
| **7 themes emerged from the study.**  **1:** Psychiatric nurses saw themselves as different from other disciplines of nursing. Felt that other nursing disciplines viewed them as inferior due to them having more male nurses and being more militant. But having better interpersonal skills than general nurses.  **2:** Psychiatric nurses felt that they have worked within a system that does not necessarily support them  **3:** Interpersonal/caring role central to psychiatric nursing. Suggestion that psychiatric nurse role is paternalistic and designed to protect patients from society and give them a level of functioning within it. Re-establishing patients back into society.  **4**: Psychiatric nursing discipline is predominately governed by the medical profession. Power relations within MDT are uneven.  **5:** Psychiatric nurses expressed a positive attitude about mental health care in the community but at the time, most was still institutional.  **6:** Most psychiatric nurses admitted having had progressive attitudes about mental health care which were informed by education and experience.  **7:** No participant spoke about philosophies or models of nursing without being prompted. It was implied that their value was limited. | | **213.**  **213.**  **213.**  **213-214.**  **214.**  **214.**  **214.** |

**Data Extraction 4.**

| Study Reference: Bray J. (1999) An Ethnographic Study of Psychiatric Nursing. *Journal of Psychiatric and Mental Health Nursing.* **6**, 297-305. | | |
| --- | --- | --- |
| Person extracting data: Donagh O’Brien. | Date data extracted: 21/05/2024. | |
| Aim of Study: To investigate what constitutes a beneficial relationship between a nurse and patient. To find the meaning of Professional Closeness for a trained nurse. To establish under what conditions professional closeness occurs and which factors prevent it from happening. | | |
| Study design: Qualitative. Ethnographic. | | |
| Description of study setting/location(s): UK. 3 unspecified psychiatric units. | Study period (dates study was done): | |
| Sampling method (e.g. purposive, convenient, etc.): Opportunistic. | | |
| Inclusion and Exclusion criteria: None specified, opportunistic sample. | | |
| Final numbers included in the study: 15 trained nurses. | | |
| Description of the recruited sample (e.g. demographics, etc.) Ward staff, | | |
| Description of data collection method (e.g. type of interviews, observations, location of data collection, etc.) Overt observation of interactions between nurses and patients. Overt observation of activities on the ward and questioning staff about observations. Semi-structured interviews with trained staff. Informal discussions with patients and untrained staff. | | |
| Description of data analysis method (e.g. content analysis, thematic, etc.): Author stated that in Ethnography, data analysis is not considered a distinct stage of research.6 step process described in this case: listening to data to gain meaning, extraction of significant statements followed by formulation and integration of statements into themes and phenomena of interest and formulation of statement of essential structure. | | |
| **Findings:** All data that reflects views or experiences of the phenomenon under investigation | | Location/ page in text |
| **Findings show different experiences of working closely with patients in a psychiatric unit. Highlights the patient focus of the profession. 3 themes:**  **1: The difficulty of working closely with mentally disturbed individuals.**  Participants describe strong emotional reactions and talk about personal cost of various patient behaviours such as bad behaviour or self-harm or suicide.  *“Through the illness, the bad times you get to know the person. Like J can be a B…but underneath this is a kind nice person.”*  *“Working with them makes me feel closer, building a partnership, building a rapport. If you don’t like them, it’s another challenge, yes it is..I don’t like every patient I work with but there’s something I can identify to work with because if I understand what’s happening to them, that’s what helps me.”*  **2: Maintaining Distance.**  Close observations/specialing of patients can be intense and difficult.  “Close observations can be destructive because we use bank staff who don’t know the patient or their motives.”  **3: Congruent care.**  Dissonance among participants about aspects of their work, notably spending time with patients is unsupported by the institution.  Participants show enormous motivation and commitment to their patients.  *“We’re able to offer people a place where they feel safe. There’s a chance to talk to people who care about what they’re going through. Being there and being able to listen, they don’t feel valued and that’s what they need.”*  *“Connecting with them, helping them feel accepted, respect are what it’s all about*”  Article highlights patient focus, challenges and emotional aspect, as well as invisibility of mental health nursing. | | **300-301.**  *300.*  *301.*  **301-302.**  **302-303.**  *302.* |

**Data Extraction 5.**

| Study Reference: Savio M. (1991) Psychiatric Nursing in Italy: An Extinguished Profession or an Emerging Professionalism. *The International Journal of Social Psychiatry.* 37 (**4),** 293-299. | | |
| --- | --- | --- |
| Person extracting data: Donagh O’Brien. | Date data extracted: 21/05/2024. | |
| Aim of Study: To investigate if the professional identity of psychiatric nurses has changed since moving to another care setting, to investigate how, despite them having a custodial role in the past, psychiatric nurses were employed as key agents to make the change and lastly, to find out how psychiatric nurses fit into the multi-disciplinary teams which are key in new system. | | |
| Study design: Qualitative. | | |
| Description of study setting/location(s): A Community Mental Health Centre (CMHC) IN Piedmont, Italy. Catchment area of 37,000. A walk-in service open mon-fri, 8am -18pm, attached to local general hospital where there is a psychiatric ward. Counselling/Psychotherapy and home visits offered. | Study period (dates study was done): Not specified in text. | |
| Sampling method (e.g. purposive, convenient, etc.): Convenient sample. Location selected first. | | |
| Inclusion and Exclusion criteria: Not specified. | | |
| Final numbers included in the study: 9 participants. | | |
| Description of the recruited sample (e.g. demographics, etc.) 9 nurses, all of whom had previously transferred from the local hospital to the community setting. Some had more than 10 years’ experience and some more than 20 years’ experience. | | |
| Description of data collection method (e.g. type of interviews, observations, location of data collection, etc.) Data collected via 2 rounds of open ended semi-structured interviews. Interviews lasted approximately 1 ½ hours. Field work, consisting of observation of participants also took place. | | |
| Description of data analysis method (e.g. content analysis, thematic, etc.): Brief account of process in text, transcribing of tape-recorded interviews. | | |
| **Findings:** All data that reflects views or experiences of the phenomenon under investigation | | Location/ page in text |
| **Findings from this study were presented in Tables which showed differences, if any, in certain nursing activities between the hospital and community setting.**  Highlights factors in transition from institutional setting to community setting.  Medication administration was the same in both settings.  Medication monitoring was greater in the community.  Helping patients with personal hygiene was greater in the hospital.  Talking with clients was the same in both settings.  Meeting clients’ relatives and home visits were greater in the community.  Leisure activities with clients and counselling were also greater in the community.  More of a feeling of autonomy in hospital perhaps due to them being more familiar with the environment.  Some resistance by some to move due to having to give up autonomy to work in multidisciplinary team.  Others welcome move to more progressive model of care. | | **295-298.** |

**Data Extraction 6.**

| Study Reference: Sercu C., Ayala R.A. & Bracke P. (2015) How does Stigma Influence Mental Health Nurse Identities? An Ethnographic Study of the Meaning of Stigma for Nursing Role Identities in Two Belgian Psychiatric Hospitals. *International Journal of Nursing Studies.* **52**, 307-316 | | |
| --- | --- | --- |
| Person extracting data: Donagh O’Brien. | Date data extracted: 22/05/2024. | |
| Aim of Study: To explore how stigma may give meaning to mental health nurse identities. | | |
| Study design: Qualitative Case Study Design. | | |
| Description of study setting/location(s): 4 wards in 2 psychiatric hospitals in Ghent, Belgium. Location deals with patients with dual-diagnosis, mental illness and substance misuse. The 2 hospitals had different models of care. The first was focused on a diagnostic approach as a basis for care, the second used a social model. | Study period (dates study was done): Study conducted between 2011 and 2012. | |
| Sampling method (e.g. purposive, convenient, etc.): Purposive sampling, candidates taken from 2 specially chosen locations. | | |
| Inclusion and Exclusion criteria: Nurses with specialist mental health nurse qualification. | | |
| Final numbers included in the study: 33. | | |
| Description of the recruited sample (e.g. demographics, etc.) Specialist mental health nurses. Participants aged between 26 and 58. 61% female, 39% male. All educated at least to degree level. Some participants had master’s degrees in psychology or philosophy. | | |
| Description of data collection method (e.g. type of interviews, observations, location of data collection, etc.) Observation of nurses at work on ward at specific times of the day, and semi-structured interviews. | | |
| Description of data analysis method (e.g. content analysis, thematic, etc.): Data analysed using Nvivo software. Constructivist grounded theory approach. Coding and thematic analysis. | | |
| **Findings:** All data that reflects views or experiences of the phenomenon under investigation | | Location/ page in text |
| **5 themes.**  **1: Reasons for wanting to become a mental health nurse?**  *“In the beginning, I wanted to be a general nurse, but my internship was so disappointing. I thought for example to pass by some service users and say hello, but I noticed that the nurses didn’t appreciate it. After I choose psychiatry and that was it. Just the fact that you don’t wear a uniform and the way they dealt with service users, really working with them, that’s what really attracted me” Hanne.*  *“I chose to work as a mental health nurse because general nursing is very technical. The contact with service users is medicalised and hospitalisations are too short which means that people are approached in a less human way” Ringo*  **2: Meaning of stigma for nursing identities.**  Some MHNs are motivated to counteract the discrediting treatment of patients with mental illness.  Differences between some nurses internalised motivation and role expectation.  **3: Being their equal.**  Nursing identity rooted in standing up for mentally ill patients or taking a stance against negative societal depiction of mentally ill people. Positioning themselves as equal to patients.  Unclear diagnoses? Patients don’t need to be excessively labelled.  *“It is very important that people can be who they are without their undefined problems. When you talk about people with a personality disorder, you talk about all of us, I don’t know anyone in this world who doesn’t have a personality disorder”* Peter.  MHN see their role as in opposition to stigma.  **4: Living a nursing dilemma.**  Conversely, Some nurses define their role by the type of patients they look after.  Disparity between nurses use of diagnosis as a descriptor as opposed to the anti-stigma approach. Tension between the diagnostic point of reference and nurses beliefs that this is stigmatising.  **5: Nursing and stigma in a therapeutic framework.**  Diagnostic frame of reference can help shape therapeutic frameworks and helps inform role expectations.  *“I think it’s good to focus on a specific diagnostic profile, because people with a pure depression often say they didn’t find help in places with a mix of different diagnostic profiles” Lore.*  Overall, nursing identity is divided between humanistic, anti-stigma POV and medical diagnostic POV which is stigmatising but may also be helpful. | | 310-311  *310*  *311*  311.  .  311.  *311.*  312-313.  313.  *313.* |

**Data Extraction 7.**

| Study Reference: Terry J. (2020) “In the Middle”: A Qualitative Study of Talk about Mental Health Nursing Roles and Work. *International Journal of Mental Health Nursing.* **29**, 414-426. | | |
| --- | --- | --- |
| Person extracting data: Donagh O’Brien. | Date data extracted: 23/05/2024. | |
| Aim of Study: To examine how talk about mental health nursing work, roles and identities was handled by participants from multiple perspectives. | | |
| Study design: Qualitative, Thematic Analysis. | | |
| Description of study setting/location(s): Study set in Wales UK. Mental health nurses recruited from former cohorts of students from one unnamed University. Service users recruited from charitable organisations. Focus groups recruited from one unnamed University | Study period (dates study was done): Not stated in text. | |
| Sampling method (e.g. purposive, convenient, etc.): Purposive sampling method. Specific recruits sought. | | |
| Inclusion and Exclusion criteria: Service user participants must have had some contact with a mental health nurse within the past 10 years. | | |
| Final numbers included in the study: 17 mental health nurses and 13 mental health service users. | | |
| Description of the recruited sample (e.g. demographics, etc.) 17 MHN. 16 women 1 man. Between 4 months and 18 months experience. Variety of working locations. | | |
| Description of data collection method (e.g. type of interviews, observations, location of data collection, etc.) semi-structured interviews with mental health nurses and service users. Focus groups with 3 groups of mental health nursing students | | |
| Description of data analysis method (e.g. content analysis, thematic, etc.): Transcripts read, re-read and coded. | | |
| **Findings:** All data that reflects views or experiences of the phenomenon under investigation | | Location/ page in text |
| **5 themes.**  **1: Nurses are coordinators of everybody else.**  One nurse struggled with the role and with others perceptions of role.  *“I just get the sense that nurses have had everything taken away from them. They’ve been left with this position of supposed professional status, but actually we can’t do anything. OTs, physiotherapists, doctors, social workers, they’re the ones with the proper roles, and it seems to me the nurse in the middle coordinates it.” Laura.*  *“I’ve had conversations with service users where they’ve said, well you know my nurse, yes, she’s someone who pops out once a month or I come into the mental health team base to see her once a month, and she’ll give you an injection or something, that’s her job. But other people see you as their main port of call really.” Angela.*  Care-coordinator a stipulated MHN role in wales.  **2: Nursing work limited by coordination.**  Role ambiguity affects professional identity.  *“There’s no, ‘we refer to the social worker’ because as care coordinators we do that, And I find it limits our nursing role massively. You know, if we didn’t have to do all these housings and referrals and everything like that, we’d be able to do the actual nursing with the patient which, to be honest with you I never do.” Ben.*  **3: Bridging the gap.**  Service users think that MHNs bridge the gap between disciplines. Sian service user.  **4: Jack of all trades, master of none.**  MHN needs to have a multitude of different skills in order to carry out their multi-faceted duties. Role is very diverse.  *“I think you are that Jack of All Trades, Master of None in some respects, or you become very exceptional in the area in which you work. ‘Cos even though we’re registered mental health nurses, I think you specialise within the area of which you work, that’s where the expertise comes from.” Emma.*  “*I agree that you need to be in some level a specialist in what you do, rather than a jack of all trades. Erm, you know, I’m not saying that we should do absolutely everything. I’m not an occupational therapist, you know they’ve got skills and knowledge that I certainly don’t have. Erm, I’m not a psychologist or whatever. Erm, but a lot of it I think well, why can’t we do that, if its part of our role? If it’s going to improve services, or improve me as a nurse or improve outcomes for the people that I’m working with, then you know why can’t we do that? Just because we’ve always done it that way, doesn’t mean we’ve got to carry on doing it” (Angela).*  Specialising also might help reduce the idea of MHN being lacking in specific skills and knowledge.  MHN could be accused of stealing roles from other disciplines.  Specialisation maybe comes with experience.  **5: Being in the middle.**  *“I just get a feeling that perhaps in my experience, I’m treated like a bit of a skivvy to be honest. Lack of respect, I think for the role, especially by families.”* Laura.  MHN has central role.  Public doesn’t fully understand it and has a bad attitude towards it | | 418.  *418*  *418*  419.  *419*  420-421.  *420*  *421*  421.  *421.* |

**Data Extraction 8.**

| Study Reference: McCrae N., Askey-Jones S. & Laker C. (2014) Merely a Stepping Stone? Professional Identity and Career Prospects following Postgraduate Mental Health Nurse Training. *Journal of Psychiatric and Mental Health Nursing.* **21**, 767-773. | | |
| --- | --- | --- |
| Person extracting data: Donagh O’Brien. | Date data extracted: 23/05/2024. | |
| Aim of Study: To explore facilitators and barriers to professional identity in newly qualified nurses of accelerated postgraduate mental health nursing training. Also to examine the influence of individual perspectives and training experience on socialisation and career plans. | | |
| Study design: Qualitative, Grounded Theory. | | |
| Description of study setting/location(s): Kings College London, Postgraduate diploma in mental health nursing. | Study period (dates study was done): 2012 | |
| Sampling method (e.g. purposive, convenient, etc.): Grounded theory theoretical sampling until saturation of responses/ no new information. | | |
| Inclusion and Exclusion criteria: | | |
| Final numbers included in the study: 10 participants. | | |
| Description of the recruited sample (e.g. demographics, etc.) 10 participants from diverse academic backgrounds, anthropology, psychology, English, sociology and management. 9 female, 1 male. 8 still working in nursing, 2 have left profession. | | |
| Description of data collection method (e.g. type of interviews, observations, location of data collection, etc.) Semi-structured interviews and user-focused monitoring. | | |
| Description of data analysis method (e.g. content analysis, thematic, etc.): Interviews were recorded and transcribed verbatim. Thematic analysis using Nvivo software. | | |
| **Findings:** All data that reflects views or experiences of the phenomenon under investigation | | Location/ page in text |
| **6 themes emerged from data.**  **1: Motives for choosing nurse training.**  *“I don’t want to say that it was a means to an end but it kind of was. I was trying to get into clinical psychology but I had a pretty average degree. I tried for a couple of years and then decided I wanted to just start working in mental health in some front-line role, so I chose nursing.”*  Others said they had caring traits, liked listening to people and understanding their problems.  **2: Conceptualisation of nursing.**  Some participants found MHN role hard to define.  Some felt community was more distinct than hospital because of more clearly defined duties. Psychological intervention just everyday interaction with patients.  Nursing seen as less structured than psychology.  “*We’re not working on these patients for six weeks or however long they’re going to be part of the intervention, its ongoing, it can be for years, and the level of support can change.”*  Tolerance, flexibility, calmness and a sense of humour seen as important characteristics.  Identity constructed around nursing values and presence.  *“Literally being there for the patient is the biggest part of nursing and that’s something which is not given full credit.”*  **3: Experience in nursing.**  Clinical placements were formative for students.  Some mentors seen as role models.  Qualified nurses who juggled many tasks and still me patients’ needs were highly regarded.  Some qualified nurses were seen negatively, e.g. “Burnt Out”.  Academic status of MHN helped strengthen participants’ professional identity despite theory practice gap.  *“Knowing that there’s a whole academic discipline of mental health nursing, because when you go onto a ward, you don’t think this is informed by academe or the latest evidence-based-practice. So it was good to know that an academic tradition exists, even though it may not be visible on a day-to-day level on shift.”*  **4: Social Identification.**  Participants proud to qualify as MHN.  *“I feel very passionate about nursing as a career. I had a few status issues with it before, which sounds really naff, but I came from a really posh school. In went back to my ten-year reunion and everyone’s a lawyer. Nursing was never considered a respectable qualification but having done it, it’s literally the best thing.”*  Some students felt a status differential between other professions and nursing.  *“A doctor was like, ‘you’ve done a degree in psychology-why are you being a nurse?”*  Nurses transcending traditional status demarcation more evident in community.  Nurses tend to come from lower educational attainment and lower socio-economic status than other professions.  Participants aware of disciplinary culture, where nurses are worried about being struck off.  **5: Institutional Identification.**  NHS preferred employer because of opportunities and resources.  Staff shortages persist in NHS.  *“I get the sense that in the public sector, you can just come along, and you can be very lazy if you want to, and you don’t have to work to a high standard. Standards are very low, whereas in the private sector, you have to be good, or you don’t get work. The private sector feels much harder and more ruthless, but I quite like that. I like people demanding a high standard (51).*  **6: Future career plans.**  Participants took different career paths on qualification.  Some worked in inpatient setting.  Some continued training in other skills such as CBT.  Some considered doing PHD, | | 769  *769.*  769-770.  *770*  *770.*  *770.*  770  *770*  771  *771.*  771. |

**Data Extraction 9.**

| Study Reference: Hurley J. & Lakeman R. (2011) Becoming a Psychiatric Mental Health Nurse in the UK: A Qualitative Study Exploring Processes of Identity Formation. *Issues in Mental Health Nursing.* **32**, 745-751. | | |
| --- | --- | --- |
| Person extracting data: Donagh O’Brien. | Date data extracted: 04/06/24. | |
| Aim of Study: To understand the processes by which Mental Health Nurses (MHNs) reached their current identities both professionally and personally. | | |
| Study design: Qualitative/ Direct Phenomenology. | | |
| Description of study setting/location(s): England and Scotland. 3 NHS mental health trusts and 2 Universities. | Study period (dates study was done): Not stated. | |
| Sampling method (e.g. purposive, convenient, etc.): Purposive sampling. Participants invited to take part in study by email. | | |
| Inclusion and Exclusion criteria: Participants had to be qualified MHNs who practiced or were engaged in delivering talking therapy. | | |
| Final numbers included in the study: 24. | | |
| Description of the recruited sample (e.g. demographics, etc.) 5 participants had 0-10 years MHN experience, 10 had 10-20 years’ experience, 9 had 20+ years’ experience. 13 participants were female and 11 were male. 7 participants were based in the research base in England, 9 at one of two Scottish bases (A) and 8 at Scottish base (B). 4 participants were educated to diploma level, 10 to degree level and 10 to masters’ level. 4 had no therapy qualification, 11 had done a short course and 9 had a formal qualification. 4 participants were in a core academic role, 3 in a core managerial role, and 7 in core clinical roles. | | |
| Description of data collection method (e.g. type of interviews, observations, location of data collection, etc.) Semi-structured interviews, which lasted approx. 1 hour and were conducted in the participants’ work environment. | | |
| Description of data analysis method (e.g. content analysis, thematic, etc.): Transcribed data was repeatedly read, in accordance with phenomenological approach, in order to find meaning beyond superficial level, such as issues relating to the self or individual reality construction. Nvivo qualitative software was used to assist data analysis process. | | |
| **Findings:** All data that reflects views or experiences of the phenomenon under investigation | | Location/ page in text |
| **4 themes emerged from the study.**  **1: Identity journeys through direct and vicarious work-based experience with service users.**  Nearly all participants identified their work with service users as well as their learned roles and knowledge from experienced staff during the socialisation process as key to forming their nursing identity.  *“I think things like role modelling and coaching, exposure to good clinicians, getting a chance to hear these good clinicians talk out loud about what they do. Working with people and getting feedback has also helped” (research participant 12)*  *“I think it’s (MHN) fascinating and it makes you grow and it’s not just the bizarre realities, it’s the everyday realities. It’s what people need out of life. A big chunk of me is the patient’s I saw.” (RP 2).*  **2: Identity journeys through non-work-based education and training.**  Nearly all participants spoke about the influence of education and training on their professional identity. Formal education and training led to a higher level of professional worth. Formal education and training advanced participants’ clinical practice.  *“I think a training course in CBT allowed my practice to become more structured, what I’ve found is I’ll work and do a much fuller assessment than I ever did before.” (RP 23)*  *“I can make a difference by using psychological approaches. You then want to focus more on the counselling aspect of the mental health nursing role, because that’s where you see yourself most effective.” (RP 3).*  **3: Identity journeys through assuming new job titles and roles.**  Much of the sample spoke about how new job titles and advanced training affected their professional identity.  *“I’m not really too sure about my professional identity at the moment, because my title here is CBT therapist. I’m not a nurse therapist, I’m a CBT therapist and in some way, I know I’m still an RPN.” (RP 17).*  Internal divisions and hierarchies within mental health nursing.  *“I used to refer to myself as a nurse but now I refer to myself as a CPN, as if to distance myself from inpatient nursing” (RP 16)*  Newly assumed titles and roles through further education create ambivalence to some about their professional identity but also created a valued distinction between them and others. Giddens (1997) stated that an individual’s name or title is very important to group identity.  **4: Exit journeys.**  Just over half of the participants effectively left mental health nursing when they assumed new roles or titles which led to a new identity. This is most prevalent in later career participants.  *“MHNs are leaving the profession and aligning themselves elsewhere. Almost as if they, at some subconscious level, have hit at an idea that they need to latch onto a different profession because this one is going, and other professions have more clout.” (RP8).* | | 747  *747*  *747.*  747-748.  747  748  748.  *748.*  748. |

**Data Extraction 10.**

| Study Reference: Hurley J. (2009) A Qualitative Study of Mental Health Nurse Identities: Many Roles, One Profession. *International Journal of Mental Health Nursing.* **18,** 383-390. | | |
| --- | --- | --- |
| Person extracting data: Donagh O’Brien. | Date data extracted: 04/06/2024. | |
| Aim of Study: To strengthen understanding of mental health nurse identities in order to ensure future role evolution in keeping with MHN opinion on future direction of profession. | | |
| Study design: Qualitative/ Phenomenological. | | |
| Description of study setting/location(s): Local health trusts in England and Scotland. | Study period (dates study was done): 2008. | |
| Sampling method (e.g. purposive, convenient, etc.): Purposive sampling. Participants recruited via written invitation to their management. | | |
| Inclusion and Exclusion criteria: Mental health nurses. | | |
| Final numbers included in the study: 24. Initially 25 but one participants’ data was unusable. | | |
| Description of the recruited sample (e.g. demographics, etc.) 5 participants had 0-10 years’ experience, 10 had 10-20 years and 9 had 20=years. 13 participants were female and 11 were male. 7 participants were English based and 17 were Scottish based. 4 participants were educated to diploma level, 10 to degree level and 10 to masters’ level. | | |
| Description of data collection method (e.g. type of interviews, observations, location of data collection, etc.) Semi-structured interviews. | | |
| Description of data analysis method (e.g. content analysis, thematic, etc.): Interpretivist stance. Transcribed interviews read and coded using NVivo software. | | |
| **Findings:** All data that reflects views or experiences of the phenomenon under investigation | | Location/ page in text |
| **7 themes emerged.**  **1: MHN as a generic specialist.**  All participants stated that mental health nursing involves the need to respond to a wide range of patient needs, physiological, psychological and social.  *“The nurse, who is perhaps doing some sort of loose term psychological supportive therapy one moment, might the next moment be taking their pulse, or dressing a wound, and the next moment, might actually be helping them to sort their housing and the next moment possibly having a game of scrabble with that same person. Now I’ve never seen a psychotherapist, or, you know a clinical psychologist playing scrabble or fill in a housing form.”* (RP 24).  MHN ability to respond to a wide variety of needs is a source of pride.  “Jack of all trades” or “all-rounder” used in literature to describe variety of duties.  Participants see varied role as important part of their identity and something that no other discipline can do.  **2: MHN as having service user focus.**  Most participants saw this as key aspect of MHN identity and saw patient focus in MHN as more prominent than other disciplines.  *“I would say I work with people to support and promote their recovery in their self and well-being whatever that may be to them and it’s individual, everybody is on an individual journey.” (RP 20).*  Participants viewed their roles as being in response to service user need. However, in seeking to meet individual service user need and be a generic specialist, the MHN role has become very wide ranging.  **3: MHN as positioning and utilizing the personal self.**  Participants saw the therapeutic use of self as key to professional identity and more than other disciplines.  *“MHN allows you to develop that sense of personhood, and I think It’s more of a journey, a personal journey than being, say, a general nurse.” (RP 15).*  However, individual and personal aspect of interpersonal relationships, might be at odds with attempts to regulate professional roles and find group identity.  **4: MHN as spending time with service user.**  79% of participants said that MHN spend more time with patients than other disciplines. Time is essential to building therapeutic relationships.  However, some literature says that time not always utilized with patients, such as in an acute ward.  **5: MHN delivering talk-based therapies in versatile ways.**  75% of participants said they deliver talk therapy in unique ways outside of formal environment.  Literature says that MHN improve access to therapy for some patients.  *“We have garnered many things from other professions to make up what we are and I was a bit uncomfortable with that because I thought, what are we then? But as I went further on and looked, it’s that bit about bringing many things together and it’s how we put them together. There’s no other profession that combines all these different things in the way MHN does and then delivers it back.” (RP8).*  However, for MHN to deliver interventions like CBT, it will take much commitment from MHN as well as organisational and financial support.  **6: The MHN as having an everyday attitude.**  58% of participants thought that common sense a key part of MHN identity compared with other disciplines.  *“I do believe that MHN do this; they play up their common sense, you know, all those darn psychologists and their high-faluting ideas. What we’ve got, yes we have got ideas, but we can translate it into everyday, normal relationships, where people like our patients are.”* *(RP 1).*  *“You can do things on a practical level for patients like sort out benefits or organise housing.” (RP 23).*  However, this may reinforce idea that MHN is nothing special as opposed to focused specialists.  **7: MHN as having transferable skills.**  50% of participants felt that MHN skills could be transferable into offering talk-based therapies. | | **385.**  *385.*  **386.**  *386.*  **386.**  *386.*  **386-387.**  **387.**  *387*  **387.**  *387.*  *387.* |

**Data Extraction 11.**

| Study Reference: Humble F. & Cross W. (2010) Being Different: A Phenomenological Exploration of a Group of Veteran Psychiatric Nurses. *International Journal of Mental Health Nursing.* **19**, 128-136. | | |
| --- | --- | --- |
| Person extracting data: Donagh O’Brien. | Date data extracted: 04/06/2024. | |
| Aim of Study: To explore the lived experiences of a group of veteran psychiatric nurses in order to identify the factors which have influenced their continuation in the area of psychiatry. This against a backdrop of negativity about psychiatric nursing. | | |
| Study design: Qualitative. Heideggerian Phenomenological Hermeneutic. | | |
| Description of study setting/location(s): Australia. An unspecified acute unit. | Study period (dates study was done): Not stated. | |
| Sampling method (e.g. purposive, convenient, etc.): Phenomenological sampling. A poster was put up in acute unit where hospital and community staff would see it, seeking volunteers. | | |
| Inclusion and Exclusion criteria: Psychiatric nurses with over 10 years’ experience. | | |
| Final numbers included in the study: 7, at which number data saturation was reached. | | |
| Description of the recruited sample (e.g. demographics, etc.) 4 women and 3 men. | | |
| Description of data collection method (e.g. type of interviews, observations, location of data collection, etc.) In depth interviews lasting 1-1.5 hours. Interview began with an invitation to describe how participant first entered psychiatric nursing and was led from then on by participant. | | |
| Description of data analysis method (e.g. content analysis, thematic, etc.): Thematic analysis. Transcribed interviews read and re-read to identify themes. Coding and search for meaning followed. | | |
| **Findings:** All data that reflects views or experiences of the phenomenon under investigation | | |
| **Main theme was Being Different.**  **Participants were positive about being Psychiatric Nurses (PN). They felt comfortable in their role as PN.**  Participants identified individual attributes which helped them in their role as PN but also set them apart from other nurses and society in general.  *“We are separate to other disciplines. It’s the attitude we have towards our patients. We’re not particularly intimidated or don’t feel threatened constantly by people who are potentially aggressive. With medical nursing, where there’s a physical problem, that’s what they’re there to do, they don’t look at other issues. We’ve a greater range of skills (Robert).”*  Robert also said that PN look different, mainly because they don’t wear uniforms.  *“Years ago at parties, people would say, ’what do you do?, I’d say, ‘I’m a nurse,’ and they’d say ‘where do you work?’ and then they’d often say ‘oh, you’re a psych nurse. They’d say, ‘oh you must be patient’ or ‘oh you must be hard or tough.’ It was the community’s perception, the extreme perception of what madness and psychiatry is all about.”* *(Frank).*  Image of PN is influenced by history of psychiatry and control.  “The difference between PN and patient is sometimes hard to tell, other than who has control of the keys.” (Veronica).  Self-confidence identified as a useful attribute.  *“I think you have to be able to accept a bit of a shock or you wouldn’t stay in the job, so that makes us a bit different because I’ve found a lot of nurses are very conservative. I think we are a different breed because we aren’t shocked or horrified by what’s at work every day” (Elizabeth).*  “*Common sense. I suppose you do have to have a lot of self-confidence. A lot of the clients look up to you. You can’t afford to be too judgemental” (Margaret).*  Nurses own perceived vulnerability is the same as patients.  *“Others might see my patients as different, but I don’t. they are sick people just like us, not an aberration. Some of us haven’t got it or we don’t know about it yet.” (Veronica).*  Curiosity towards mental illness and understanding why people with mental illness are marginalised.  *“I do think that the curiosity of the troubled mind rather than the sick brain, and I make that distinction, is something that I suppose, even before I became a psych nurse, has always fascinated me” Frank.*  Assertiveness is part of the PN role and there may be political factors which contribute to different culture in PN.  *“The history of a strong and militant PN union makes it easier for us to stand up for patients and fight injustice.” (Robert)*  *“Advocating for patients is my moral duty.” (Frank).*  Acceptance and use of self is also seen as an important attribute for PN.  *“And just my own feelings about what people need. I think really the bottom line with people is that they like to be listened to and your goals for people…you don’t have to sort of have them on Mars, the fact that they might be able to go down the street within a few months of a major mental illness, and you’ve helped them to sort out ways of getting back into the workforce, or getting back to socialising and communicating again with people. That’s the goal for me, that’s what its all about.” (Anne).* | | **131-133.**  *131.*  *132*  *132*  *132*  *132*  *132.*  *132.*  *133.* |

**Data Extraction 12.**

| Study Reference: Holyoake D. (2002) Male Identity in Mental Health Nursing. *Nursing Standard.* 16 (**48**), 33-37. | | |
| --- | --- | --- |
| Person extracting data: Donagh O’Brien. | Date data extracted: 04/06/24. | |
| Aim of Study: To explore how gender representations affect mental health nurses’ sense of identity. To explore the cultural meaning associated with male mental health nurses. | | |
| Study design: Qualitative. Ethnography. | | |
| Description of study setting/location(s): 3 acute mental health units. One unit in London and 2 in Birmingham. | Study period (dates study was done): Over 18 months. Dates not stated. | |
| Sampling method (e.g. purposive, convenient, etc.): Convenience sampling. One of units used was close to the researcher. Male nurses/Informants from the units were also chosen opportunistically. | | |
| Inclusion and Exclusion criteria: Male psychiatric nurses. (MPN) | | |
| Final numbers included in the study: Not clear in text. | | |
| Description of the recruited sample (e.g. demographics, etc.) Male mental health nurses. | | |
| Description of data collection method (e.g. type of interviews, observations, location of data collection, etc.) Observation/Shadowing and interviews with participants. | | |
| Description of data analysis method (e.g. content analysis, thematic, etc.): each participant was interviewed multiple times as well as being shadowed so a lot of data was collected over time. Themes were subsequently identified. | | |
| **Findings:** All data that reflects views or experiences of the phenomenon under investigation | | Location/ page in text |
| **4 themes emerged from the data.**  **1: Constructing Identity.**  Male nurses have a sense of self and present the image they want to portray to others. This image is fashioned within nursing culture and clinical experience.  *“When I was doing my stint in general nursing, which in those days was 3 months, I noticed that there was a marked difference between male and female nurses. For example, I was called Mr Smith while females in my group were called student nurse.” (Ally)*  Technically no difference between male and female nurse but males said that they have to portray maleness in a nursing way. “Maleness”. A hybrid nursing identity constructed by others for male nurses. Psychiatric nursing suggests that males are necessary for strength and restraint. Cultural stereotypes influence how male nurses are seen in psychiatry and how they see themselves.  **2: The sex difference perspective in practice.**  General nursing has always been seen as a female profession. There is a smaller percentage of men in nursing than women but a higher percentage of men in nurse management roles.  *“I think that society actually views women as being more sort of nurturing and caring and that it’s a special type of male that actually comes into nursing in the first place. There are more males in psychiatry and I think this is historical, you, taken from the old days of Victorian asylums when the mad really did need someone to sit on them” (Anthony).*  Societal views on gender in nursing persist but nurses in general seen as less dominant or aggressive regardless of gender. Male nurses seen as more gentlemanly than most males. All nurses seen as feminine or emotionally unstable. Male nurses seen as more in touch with their feminine side but more logical and resilient than female nurses.  Most male nurses feel they should be viewed as sexless.  *“I think male nurses should be thought of as sort of sexless, they should be able to go into female rooms and nurse like the women do. I don’t think the sex of a nurse should matter, it should be equal.” (Johnny).*  **3: Boys will be boys.**  Men are biologically different to women and must conduct themselves accordingly.  “I wouldn’t go into a female dormitory to wake females.”  “Females come up to me and talk. Perhaps they relate better to males.” (Liam).  “I always leave the door open when I’m alone with a female patient.” (Brian).  **4: Soft masculinity.**  Male nurses perceived to be more in touch with their feminine side or ‘soft’. Male nurses identity with ‘soft masculinity’ which is different to masculinity in other professions.  Male nurses working in the shadow of masculine male nurses from the past and trying to live up to these expectations.  *“The old charge nurse looked down at me when I was trying to restrain a patient and making a bad job of it. I wasn’t flavour of the month, he needed to call reinforcements.”*  Being to effeminate is not deemed to be a good trait. People gossip about which male nurses might be gay. It feels safer to be heterosexual and thus to belong to the most dominant group.  *“It doesn’t matter what orientation a person is, as long as they’re a good nurse. Being gay doesn’t make you any les of a man.” (Bob).*  This response underlines the caring nature of psychiatric nursing culture.  However, some nurses use homophobic slurs to protect their own male identity and some act straight.  *“I think that nursing has always offered gay men an undisturbed career. Nursing is still anti-gay, it’s just less anti-gay than other professions.”*  Male identities are personal rather than culturally designated. Male participants in this study prefer for their identity to about their work. | | 34-35.  *34.*  35.  *35.*  *35*  **35-36.**  **36.**  *36.*  *36.*  *36.* |

**Data Extraction 13.**

| Study Reference: Crawford P., Brown B. & Majomi P. (2008) Professional Identity in Community Mental Health Nursing: A Thematic Analysis. *International Journal of Mental Health Nursing.* **45**, 1055-1063. | | |
| --- | --- | --- |
| Person extracting data: Donagh O’Brien. | Date data extracted: 05/06/24. | |
| Aim of Study: To examine how Community Mental Health Nurses (CMHN) perceive their working lives. How do CMHN perceive their professional status in relation to public image compared with their understanding of their own working lives. How does the gap between their professional goals and working lives affect their feelings about their job and self-image. | | |
| Study design: Qualitative. Thematic Analysis. | | |
| Description of study setting/location(s): Various locations in UK Midlands. | Study period (dates study was done): From 2003 to 2006. | |
| Sampling method (e.g. purposive, convenient, etc.): Purposive. | | |
| Inclusion and Exclusion criteria: CMHN who work with people from age 18-65. | | |
| Final numbers included in the study: 34. | | |
| Description of the recruited sample (e.g. demographics, etc.) 26 female participants and 8 males. 30 participants were exclusively in practice and 4 combining clinical practice with teaching. Of those solely in clinical practice, 19 were at G grade and 11 at E grade. | | |
| Description of data collection method (e.g. type of interviews, observations, location of data collection, etc.) Semi-structured interviews which were allowed to evolve as dialogue between interviewer and participant continued. The third author had previously conducted fieldwork with nurses on professional identity and stress which were used as a basis for interviews. | | |
| Description of data analysis method (e.g. content analysis, thematic, etc.): Interviews were transcribed and themes extracted. | | |
| **Findings:** All data that reflects views or experiences of the phenomenon under investigation | | Location/ page in text |
| 4 themes emerged from literature.  **1: The Client Focus: The public service identity of the profession.**  Participants perceive their identity as rooted in patient well-being but are self-effacing also.  *“Professional Identity? I find that quite difficult to answer. What my exact role is. I tend to say that I’m kind for a living. I help people to help themselves. I try to enable people, empower people” (RP 019).*  This shows a tendency to put the client at the fore, leading to role ambiguity on the part of the nurse and invisibility.  *“I suppose whoever you see, you’re always thinking how can I help this person? And some of them are really wretched, and you know they haven’t been looking after themselves but there’s always something you might be able to do, even if it’s not very much” (RP 002).*  CMHNs position themselves as putting clients first, being jacks of all trades in the service of the patient or allowing blurring of roles. Thus creating difficulty creating a clear professional identity.  “*From the patient’s perspective, I think the overlap of roles might be better. It might cut out them having to tell their story to too many people. I’m not one of these resistors of blurring of roles. I’m ok as long as its client focused.” (RP 3).*  **2: Not being a profession. Scepticism, doubt and uncertainty.**  Some CMHN cast doubt over whether their career is a profession.  *“We don’t have any clout. We answer to everybody, a doctor or social worker can compulsorily admit someone to hospital but we can’t. But when everything goes wrong, it’s us who have to fix it. If were supposed to be mental health specialists, why does nobody ask our opinion? ” (RP 001).*  Some literature on the subject argues that CMHN role of sorting it all out often goes unnoticed and is invisible.  Invisibility can also be compounded by hard to define subject matter of CMH Nursing.  *“People don’t recognise it, they don’t recognise mental health problems. They think people should pull themselves together. Perhaps that’s why they don’t recognise mental health nurses as being professionals because it’s not a real illness.” (RP 19).*  CMHN role and commonly accepted role of a nurse differ.  *“It’s like with my students or anybody else, when I say I’m a nurse, they think about what they see on casualty or something, and some of them think it’s all crises but most of what you do is just looking after people really.”(RP 033).*  Occupational self-effacement at the fore again. CMHN doesn’t give themselves credit and are rendered invisible.  Gender inequalities also perceived to contribute to idea that CMHN isn’t a profession.  *“Nurses are not willing to support each other. The reason for that, not being PC is that it’s female dominated. Traditionally, it’s been that women tend to be less assertive, tolerate more, for want of a better word, crap than men will. It’s seen as a female role, its’s nurturing, caring, it’s a badly paid job. So it’s low pay and men won’t do it. And if they do it, it’s at management level away from nursing”*  Dichotomy between nurses seeing themselves as being bossed around and idea of pride in their flexibility and resourcefulness.  **3: Growing out of the role: Professional development as an exit strategy.**  Some participants undertook further training in different forms of psychotherapy, CBT, family therapy etc. Thus gaining skills from outside their profession in order to make themselves feel more professional.  *“The role has lots and lots of subtle growths within it. I use a lots of counselling principles because I felt I needed some skills development so I went off and did two counselling courses. Because I started to feel the need that.. we had to start being accountable and saying what we did was evidence based. I just felt, as a CPN I wanted to preserve some sort of integrity as our profession. And possibly my own personal feelings. I don’t want to feel put down either.” (RP, 21).*  **4: Waiting to be discovered: The search for Recognition.**  Simultaneous acknowledgement that hard work is invisible and a desire for recognition.  *“It’s like everyone wants to be famous these days and I get the impression us nurses are like that, waiting to be discovered, as if someone will come along and recognise our talents, and I’m like Don’t hold your breath darling.” (RP 34).*  *“So much of feeling good about your work and yourself when it comes down to it depends on whether other people appreciate it, like when the patients say thank you or if your manager does, if that was ever going to happen which seems pretty unlikely, but at the end of the day you’re just so dependent on the approval of other people” (RP 31).* | | **1058-1059.**  *1058*  *1058*  *1059*  **1059**  *1059.*  *1059.*  *1059.*  *1059.*  **1060.**  *1060.*  **1060.**  *1060.*  *1060.* |

**Data Extraction 14.**

| Study Reference: Reis H.F.T., Terra M.G., Dos-Santos E.M., Nasi C., Reis Junor W.M., Brito L.G.A., Kurimoto T.C.S. & Sena E.L.S. (2022) Professional Identity in Mental Health Nurses: A Phenomenological Study in Merleau-Ponty. *Revista Gaucha de Enfermagem.* **43**, e20220140. | | |
| --- | --- | --- |
| Person extracting data: Donagh O’Brien. | Date data extracted: 05/06/24. | |
| Aim of Study: To understand the professional identity of mental health nurses. | | |
| Study design: Qualitative. Theoretical-Methodological framework of Maurice Merleau-Ponty’s Phenomenology of Experience. | | |
| Description of study setting/location(s): Brazil. Within the psychosocial care network of the unified health system in Brazil. Notably 4 Psychosocial care centres ( 2 X CAPS II, CAPS AD and CAPS AD III) in the cities of Vitoria de Conquista and Jequie, Brazil. And 4 Family Health Strategies (ESF) in Vitoria de Conquista. (all community/drug rehab settings). | Study period (dates study was done): From October 2019 – April 2020. | |
| Sampling method (e.g. purposive, convenient, etc.): Purposive. Participants identified by preordained criteria. | | |
| Inclusion and Exclusion criteria: Inclusion: Nurses working consistently in the research setting for 6 months. Exclusion: Nurses who were away from work for periods due to holidays, leave, strike etc. | | |
| Final numbers included in the study: 16. | | |
| Description of the recruited sample (e.g. demographics, etc.) 14 women and 2 men. | | |
| Description of data collection method (e.g. type of interviews, observations, location of data collection, etc.) Phenomenological interviewing. Trying to establish meaning from individual experience. | | |
| Description of data analysis method (e.g. content analysis, thematic, etc.): Analytics of Ambiguity is a data analysis method designed my Merlau-Ponty where meaning is sought from ambiguity during interview. | | |
| **Findings:** All data that reflects views or experiences of the phenomenon under investigation | | Location/ page in text |
| **2 main themes.**  **1: From classical psychiatry to psychosocial care.**  MHN understand that they should establish their professional identity from the point of view of psychosocial care, but some still show knowledge and practice based on classical psychiatry. (community-based, recovery orientated care vs hospital).  *“We need to enhance the leading role of the user with his or her participation, which is sometimes difficult because even in this environment we have interference from our culture, from colleagues, but also from me, who expect him/her to do it in a standardised way, pre-established, but he will have other pathways, including his experience, which is totally out of the box.” (Isabel).*  Some participants conflicted between psychosocial care and technical skills associated with hospital.  *“I would not be willing to work in that hospital centred model. I feel good collaborating with psychosocial care, in the sense of empowering the person, making the user responsible for his/her own care. I feel better than if I were in the hospital with the person tied up, sedated or being shocked. But I have a greater professional identification with the hospital, I also work in a hospital. So here at (CAPS), there is no conflict because it is very difficult to the hospital.” (Jose).*  Other participants felt that nurses do everything, and identity was not clear.  *“I joke that I forget my role as a nurse because I don’t have a specific identity in mental health, except for the technical parts of nursing, the consultation, which includes the history, the evaluation, the physical examination. I’m more of a mental health technician. We do everything here.” (Laura).*  *“We do a little bit of everything, sometimes I consider myself more of a technician in mental health, but what I see as more specific is the issue of the clinical evaluation, the nursing consultation, anamnesis, vital signs, medication administration, continued care in intensive care.” (Ana).*  **2: Identity leap, mediated by the experience of the body itself.**  Identity of MHN outlined with the intention of pointing out strategies for transcendence of care.  *“It took me 2 years for the user to understand that he/she was self-sabotaging, for him to allow to be taken care of, apart from the time he had here with other professionals. I understood that I need to do my job every day, considering the right of the user to all the services he needs but without creating expectations which are mine.*  *Identity leap means a new insight into mental health born of experience.*  *“When I first came to CAPS, I realised that patients and other disciplines saw MHN as not having a role, but now I feel I have a unique awareness that only a nurse could have, because of my interactions with the patient.” (Eva).*  *“Everything we do here is to socialise the user, for that goal. Whether it is medication, a chat, a visit, manual activities, therapeutic follow up, any intervention, it is with the intention of improving their quality of life.”(Joao).*  *“we do a more expanded job than just technical nursing care and it makes a big difference, team work, promoting autonomy but saying ‘I’m here if you need me’, ‘I go with the patient to solve a problem, until over time, he walks alone’.”* | | **4-5.**  *4.*  *4*  *5*  *5*  **5-6.**  6.  6. |

**Data Extraction 15.**

| Study Reference: Karinakola M., Kaikoushi K., Doulougeri K., Koutrouba A. & Papathanassoglou E. (2018) Perceptions of Professional Role in Community Mental Health Nurses: The Interplay of Power Relations between Nurses and Mentally Ill Individuals. *Archives of Psychiatric Nursing.* **32**, 677-687. | | |
| --- | --- | --- |
| Person extracting data: Donagh O’Brien. | Date data extracted: 05/06/24. | |
| Aim of Study: To investigate the lived experience of Greek-Cypriot CMHNs of their professional role with a focus on their emotions and perceptions. | | |
| Study design: Qualitative. Phenomenological approach. | | |
| Description of study setting/location(s): Community mental health services in 5 locations in Cyprus. Approx 56 CMHNs are employed in these services. | Study period (dates study was done): Last semester of 2013. | |
| Sampling method (e.g. purposive, convenient, etc.): Purposive sample selected from the approx. 56 CMHNs employed in Cypriot CMHS. | | |
| Inclusion and Exclusion criteria: At least 2-years’ experience working in CMHS and willingness to communicate the lived experience of CMHN role. Ability to reflect on lived experience and ability to communicate the lived experience. Interviews conducted with candidates and final number derived based on these criteria. | | |
| Final numbers included in the study: 5 participants following data saturation. | | |
| Description of the recruited sample (e.g. demographics, etc.) CMHNs working in Cyprus who fulfilled inclusion criteria. | | |
| Description of data collection method (e.g. type of interviews, observations, location of data collection, etc.) Interviews using open-ended phenomenological questions. 1: Please describe what it is to be a CMHN? 2: How do you feel about your professional role/Please describe your thoughts about your professional role? 3: How does your professional role affect your thoughts and feelings? 4: What are your feelings, thoughts experiences about your contact with service users and their families? | | |
| Description of data analysis method (e.g. content analysis, thematic, etc.): Thematic analysis. | | |
| **Findings:** All data that reflects views or experiences of the phenomenon under investigation | | Location/ page in text |
| Core theme was power relations between CMHN and patient during home visits.  **4 main themes.**  **1: Professional role perceptions.**  Working setting is patient’s home, and it is challenging.  Being a visitor in a patient’s home.  *“We need the patient’s consent to get into their house. If they tell us to leave, we have to leave, we need to be very careful, we are just guests.” (Nina).*  *“In a clinical setting, you are the therapist and he’s the patient: Clear roles. But at his home you’re the guest and you have to be careful because the rules of the game are his.”* (Emilia).  It can be difficult to balance being a guest and getting your work done when you are in the patient’s house.  *“How can you be a visitor and function effectively at the same time? That’s art. You need skills to combine the two, skills and mastery”* (Kostas).  Compared with rules and bureaucracy in hospital setting, flexibility of CMHN role is appealing. “I find it interesting and challenging.” (Emilia).  Challenge to navigate stigma of mental illness in community setting.  *“At first, the family would refuse my visit. They are afraid the neighbours would see me enter and assume there’s a psychiatric patient in the family.” (Michael).*  Staff can feel vulnerable in patient’s home with no support.  *“Sometimes you feel insecure in there. If you feel that you’re up against an aggressive or dangerous behaviour.” (Emilia).*  Autonomy very important for CMHN but can be a double-edged sword.  *“You have your own office, your interviews with the patients, you do all the screening without being supervised by a psychiatrist or a psychologist.” (Nina).*  *“I’m alone and it’s me who needs to decide which course of action is right, this is difficult….you need the umbrella of the multidisciplinary team (Maria).”*  *“There is generally autonomy when you work with the patient, but sometimes you come to a dead end, especially when it comes to medications, your hands are tied. Or when somebody needs hospitalisation, the doctor must do the referral, I can’t make that call.” (Emilia).*  Wide range of skills and knowledge necessary to do CMHN role effectively.  *“It’s important to explain to the patient how the disease works. We also try to educate families on therapeutic issues, for example to educate them on how to recognise early stages of a relapse” (Emilia).*  Partner in a therapeutic relationship.  *“Most of the work aims at building trust and rapport in this relationship. If you don’t achieve that, then there is no common route to follow. But if you cooperate with the patient, then the whole thing is a joint effort” (Kostas).*  *“The rapport you build with the patient is your main therapeutic tool.” (Nina).*  **2: Self-awareness and self-appraisal.**  Professional practice a means towards increased self-awareness and self-improvement.  *“I learned how to be more humane and more patient in my personal life.” (Emilia)*  **3: Feelings about professional role.**  *“What makes you happy is watching the patient get better, and how happy the patient and his family are. That’s our satisfaction.” (Nina)*  *“All our colleagues care for their patients and their work, they do their best and we are good to each other.” (Maria).*  Negative feelings about role. Lack of meritocracy highlighted.  *“A lot of my colleagues have been passed over for promotion, it hurts.” (Nina).*  **4: Expectations of professional role.**  “We don’t get the credit we deserve for what we do. Maybe we should step forward. I don’t know if it’s for a lack of promotion but we need to be given a chance to be heard.” (Emilia).  *“If procedures were less time-consuming. We would have more time for patients and less relapses.” (Michael* | | **682.**  *682*  *682.*  *682.*  *682*  *682*  *682.*  *682*  *682.*  *682*  *683*  *683*  *683*  **683.**  *683*  **683-685.**  *683.*  *683.*  *685.*  **685.**  *685.* |

**Data Extraction 16.**

| Study Reference: Moir J. & Abraham C. (1996) Why I want to be a Psychiatric Nurse: Constructing an Identity through Contrasts with General Nursing. *Journal of Advanced Nursing.* **23**, 295-298. | | |
| --- | --- | --- |
| Person extracting data: Donagh O’Brien. | Date data extracted: 06/06/24. | |
| Aim of Study: To examine how final-year psychiatric nursing students construct occupational identities. | | |
| Study design: Qualitative. Discourse analysis. | | |
| Description of study setting/location(s) An unspecified Scottish university | Study period (dates study was done): Not stated. | |
| Sampling method (e.g. purposive, convenient, etc.): Purposive. | | |
| Inclusion and Exclusion criteria: First year and final year psychiatric nursing students. | | |
| Final numbers included in the study: 20. | | |
| Description of the recruited sample (e.g. demographics, etc.) 10 first year/entrant psychiatric nursing students and 10 final year psychiatric nursing students, 6 of which were pursuing registration. | | |
| Description of data collection method (e.g. type of interviews, observations, location of data collection, etc.) In-depth interviews with the sample, which focused on course and career choice. | | |
| Description of data analysis method (e.g. content analysis, thematic, etc.): Transcribed interviews analysed in relation to identification and justification of career. | | |
| **Findings:** All data that reflects views or experiences of the phenomenon under investigation | | Location/ page in text |
| 4 Themes emerged from data.  **1: Choosing nursing.**  “I just wanted to be in contact with the public. I wanted to meet people and see aspects of life that people don’t normally see, like babies being born.” (RP 1).  “I think I always wanted to do it, I took a lot of science subjects at school.” (RP 2).  “I knew about nursing from having it in my family and I wanted to do a degree.” (RP 3)  “Its something I wanted to do since I was young, and I wanted to work with people.” (RP 4).  These participants are natural born nurses.  **2: Choosing Psychiatric nursing.**  Participants justified choosing psychiatric nursing by comparing it to general nursing.  *“I enjoyed it (my mental health nursing placement) so much more than my general when I’ve been going through my training, and I really feel like I’m the kind of person who likes to sit and talk to patients. You have time in psyche. It’s not so technical orientated. It’s always get them up by 10:00 or else! in general.”*  *“When I came here first, I went on the general wards and the first of my placements was on a surgical ward. At first, I just hated it and I really wanted to leave the course and get out of it. But I stayed, and when I got to my psychiatric placements, I just really enjoyed it. You could stand up and your opinions were valued, and people would listen to you. You were given some responsibility and allowed to develop as a person. In general nursing, you’re suppressed so much, and you’re measured on how quickly you can do thing.”*  **3: Identity.**  Participants highlight the value of autonomy compared with the structured nature of general nursing.  *“I don’t really like general nursing, the structured setting it’s in. In general nursing, you’re more sort of confined and you’re constantly keeping up with new skills you need. Em, I know that I would lose, not myself, but I would be a nurse the way that sort of the public see a nurse. I chose psychiatric nursing because I enjoy talking to patients and I also felt its sort of a registered part of nursing that isn’t really seen as nursing and it’s sort of pushed under the carpet a lot*” (RP 6).  **4: Psychological nature of psychiatric nursing.**  Psychological nature of psychiatric nursing can be used both to construct professional identity based on the sociability of the work and the high level of specialist knowledge and skill involved.  *“Forget all these technical procedures. Anybody can put up a drip or put a CVP line up. I think the psychological factors are left so much on the general wards.” (RP 3).* | | **296.**  **296-297.**  *297.*  *297.*  **297-298.**  *297.*  **298.**  *298.* |

**Data Extraction 17.**

| Study Reference: White J.H. & Kudless M. (2008) Valuing Autonomy, Struggling for an Identity and a Collective Voice, and Seeking Role Recognition: Community Mental Health Nurses Perecptions of their Roles. *Issues in Mental Health Nursing.* **29**, 1066-1087. | | |
| --- | --- | --- |
| Person extracting data: Donagh O’Brien. | Date data extracted: 06/06/24. | |
| Aim of Study: To engage Community Mental Health Nurses (CMHN) in a dialogue in order to learn about their role, any concerns they have and any issues regarding job satisfaction, with a view to making recommendations for any necessary change in response to their concerns. | | |
| Study design: Qualitative. Participatory Action Research (PAR). | | |
| Description of study setting/location(s): An unnamed, large, community based behavioural health service in southeast USA, providing care for mental illness, addiction and intellectual disability. 40 CMHN employed in this service. | Study period (dates study was done): Not clear in text. | |
| Sampling method (e.g. purposive, convenient, etc.): Convenient. All invited sample were CMHN from the same service. | | |
| Inclusion and Exclusion criteria: CMHN working in the featured service. | | |
| Final numbers included in the study: 36. 40 CMHN were invited to participate, 38 completed a demographic data form, and from those, 36 eventually took part. | | |
| Description of the recruited sample (e.g. demographics, etc.) Range of experience from 7 years to 45 years with a mean of 28. 35 female, 1 male. 10 participants had associate degrees, 12 had bachelor’s degrees, 10 had master’s in nursing, 4 had master’s in social work, and 2 had PHD in nursing. | | |
| Description of data collection method (e.g. type of interviews, observations, location of data collection, etc.) Data collected by focus groups. Focus groups were between 5 and 10 members in side and lasted from 1.5-2 hours. Six groups in total. Data was written down by hand | | |
| Description of data analysis method (e.g. content analysis, thematic, etc.): Reports were written consisting of recommendations which came from focus groups. Reports were then validated with the help of the participants and management. | | |
| **Findings:** All data that reflects views or experiences of the phenomenon under investigation | | Location/ page in text |
| **3 main themes.**  **1: Struggling for an identity and a collective voice.**  All participant CMHNs felt concerned about not having a collective voice or a nursing identity. They felt that an appointed CMHN leader would be helpful to allow them to voice their frustrations  Participant CMHNs felt they have no real identity, there are no real mechanisms with a nursing focus. Issues around nursing policy were rarely addressed.  *“We spent hours on policy and procedure documents for the medication clinics and they never got implemented.”*  Nurses valued working as part of multi-disciplinary teams but really wanted a collective nursing focused voice or identity.  **2: Valuing autonomy.**  Autonomy highly valued by CMHNs, despite some systemic frustrations, and losing autonomy a big concern for CMHNs. Participants also concerned that other staff did not see them as autonomous decision makers, rather just performing nurse duties. Autonomy might dissuade new graduates from becoming CMHN causing issues regarding recruitment. Most CMHN saw autonomy as more important than salary.  *“We need to maintain the importance of autonomy despite any leadership positions we might develop.”*  *“I don’t care about my salary….that is the least important part of job satisfaction for me. I stay because of the autonomy I have caring for my clients.”*  **3: Seeking role recognition.**  CMHNs were concerned about understanding of other disciplines about their role and in turn, getting recognition for their work. They thought other disciplines only saw them as “pill pushers” and did not realise the breadth of their roles. They wanted to also be recognised for their high level of expertise in treating complex cases.  However, giving medication was perceived as how other disciplines viewed them. Role strain or conflict occurred with the necessity for CMHNs to do clerical work, which took them away from patient care, when a clerical officer could be hired. CMHNs were also frustrated about being blocked by regulations and other factors which prevented them from effectively doing their job.  *“A nurse is a nurse. It doesn’t matter how educated we are or what we can do. We are not recognised for the complex care we provide in medication clinics. We provide valuable information to team members and case managers about the client’s process.”* | | **1076-1078.**  *1077.*  **1078-1079.**  *1078.*  *1079.*  **1079-1080.**  *1079.* |

**Data Extraction 18.**

| Study Reference: Santangelo P., Proctor N. & Fassett D. (2018) Seeking and Defining the ‘Special’ in Specialist Mental Health Nursing: A Theoretical Construct. *International Journal of Mental Health Nursing.* **27**, 267-275. | | |
| --- | --- | --- |
| Person extracting data: Donagh O’Brien. | Date data extracted: 07/06/24. | |
| Aim of Study: To explore the nature, scope of practice and implications of mental health nursing in the context of discourse about its vague professional identity. | | |
| Study design: Qualitative. Constructivist grounded theory. | | |
| Description of study setting/location(s): Australia. | Study period (dates study was done): 2012-2014. | |
| Sampling method (e.g. purposive, convenient, etc.): Purposive, mental health nurses chosen for their ability to speak in depth about their jobs. A secondary sample of 5 clients and 1 healthcare provider was also included. | | |
| Inclusion and Exclusion criteria: Participants must hold a recognised specialist qualification in mental health nursing and have sufficient experience. | | |
| Final numbers included in the study: 42: 36 mental health nurses (MHN), 5 clients and 1 healthcare provider. | | |
| Description of the recruited sample (e.g. demographics, etc.) 36 MHNs, all work autonomously. 60% have a minimum of 10 years’ experience, over half group have more than 25 years’ experience. All participants from across a range of in-patient and community settings. | | |
| Description of data collection method (e.g. type of interviews, observations, location of data collection, etc.) Interviews with all 42 participants. Interviews asked about what was special or distinctive about MHN and why? How did participants come to this conclusion etc. | | |
| Description of data analysis method (e.g. content analysis, thematic, etc.): Data analysed in keeping with grounded theory approach. Data compared with other participants’ accounts as analysis progressed. | | |
| **Findings:** All data that reflects views or experiences of the phenomenon under investigation | | Location/ page in text |
| **4 main themes.**  **1: The nature and scope of mental health nursing practice.**  Mental health nursing has justifiably assumed its professional nursing status, it fits the frame of nursing work, particularly in it patient focus.  Mental health nursing occurs over a 24 hour period, in common with other types of nursing.  Special relationship with clients.  Mental illness harder to define or assess than physical illnesses, requiring specially developed skills, which are developed by many during experience in acute admission settings.  Meaning given to ordinary activities of daily living, together with client focus creates individual negotiated interventions, which sets MHN apart from biomedical model of nursing. MHN involves a wide selection of skills and resources.  Mental health nurses engage in activities beyond normal prescribed boundaries such as: taking a walk, having a coffee or solving a social or financial dilemma in order to enhance the therapeutic relationship.  “*Flexibility, humility, inclusiveness and preparedness to engage in a wide variety of interventions*.”  Clients felt respected and included.  Healthcare provider felt that nurses brought a special and different perspective.  This study hypothesises that MHN brings a unique construct to the caring role, which is characterised by collaborative relationships that bring patient focus to a new level.  **2: The consequences of nursing practice.**  Distinctive role, skills, expertise and knowledge of MHN and is specialist in nature, broad in its scope and highly influential.  **3: A core category and process of MHN practice.**  A reframing of what we already know about mental health nursing and proposed a model of care, which would provide mental health nursing with a distinct identity.  *“The psychosocial process of co-constructing care towards recovery.”*  **4: A substantive theory of mental health nursing practice.**  Recovery-oriented care characterised by collaborative nurse-patient relationship.  Participants in this study did not see themselves as in competition with other disciplines, or claim ownership of certain skills, rather they hold a special place in health systems due to their patient focus, collaborative practice and broad skills and knowledge.  *“Being in the here and now, side-by-side, co-constructing care.”* | | **270-271.**  *271.*  **271-272.**  **272.**  ***272.***  ***273.***  *273.* |

**Data Extraction 19.**

| Study Reference: Harrison C. A., Hauck Y. & Ashby R. (2017) Breaking Down the Stigma of Mental Health Nursing: A Qualitative Study Reflecting Opinions from Western Australian Nurses. *Journal of Psychiatric and Mental Health Nursing.* **24**, 513-522. | | |
| --- | --- | --- |
| Person extracting data: Donagh O’Brien. | Date data extracted: 07/06/24. | |
| Aim of Study: To examine how mental health nursing could be promoted as a sustainable career option. | | |
| Study design: Qualitative. Exploratory, cross-sectional study. | | |
| Description of study setting/location(s): A public mental health service in Western Australia. | Study period (dates study was done): | |
| Sampling method (e.g. purposive, convenient, etc.): Convenient. Invitations sent by email, or distributed as flyers or on notice boards. | | |
| Inclusion and Exclusion criteria: Mental health nurses employed in a number of settings. | | |
| Final numbers included in the study: 150, at which time data saturation was reached. | | |
| Description of the recruited sample (e.g. demographics, etc.) Sample recruited from one mental health service in Western Australia consisting of a total of 853 nurses, who work in a variety of settings. | | |
| Description of data collection method (e.g. type of interviews, observations, location of data collection, etc.) Interviews, either face to face or by telephone. Each interview lasted between 10 and 20 minutes. Interviews consisted of 2 questions; 1: Was there something specific in your nurse education which encouraged you to decide to work in mental health nursing? And 2: In your opinion, what could be done to entice nurses to work in mental health? | | |
| Description of data analysis method (e.g. content analysis, thematic, etc.): Data saturation achieved after 150 interviews, but interviews were continued until all interested participants had given their views. Thematic analysis was then conducted using recognised thematic analysis framework. | | |
| **Findings:** All data that reflects views or experiences of the phenomenon under investigation | | Location/ page in text |
| **A: Overarching theme was the effect of stigma in having a negative influence on how people view mental health nursing. There were a number of subthemes.**  **1: Visibility of mental health nursing.**  “If profile was heightened, people would be attracted to it.”  Mental health nursing should be better promoted as a career option.  **2: Self-promotion.**  Mental health nurses should take ownership of promoting themselves, set themselves up as good role models etc.  *“Nurses need to be made aware that their negativity is impacting on the service recruitment, and they don’t actively encourage students to go into mental health nursing. We as a group of mental health nurses need to take ownership” (161)*  **3: Industry promotion.**  *“The whole mental health industry is portrayed badly. There should be staff representatives to give talks at educational institutions. This would change the attitudes towards the students. It would also promote mental health nursing as a great field to work in.”*  **4: University promotion.**  Passionate mental health nurses, acting as role models would help promote mental health nursing in the university.  *“The lecturer at university encouraged me to go into mental health nursing. She told me stories about her time in mental health and they were really interesting and caught my attention. This encouraged me to start working in mental health. She was so passionate about it”* (11).  **B: Growing mental health nursing.**  **Another major theme, discussed in 4 subthemes.**  **1: Improving the student experience.**  Students should have more specialised mental health nursing content during their training.  “*There needs to be longer clinical practice in the training*.”  “*Everybody comes to mental health with preconceived ideas and they just get ‘a lick and a promise’ instead of proper experience. There should be a designated preceptor to have time to spend with the students or graduates.”*  **2: Professional development opportunities.**  *“There should be better staff development for the mental health nurses working in the services. How can we help students if nobody helps us?”*  **3: Recognising the mental health specialty.**  *“Mental health nursing should be made a proper specialty. It certainly isn’t taught well on the wards. When the mental health nurses all retire there will be no specialist nurses left.”*  **4: Valuing staff.**  Mental health nursing would be seen as more desirable if conditions for staff and patients were better.  *“There should be a zero-tolerance for all kinds of aggression and management should support this.”* | | **516.**  **516.**  **516.**  *516.*  **516-517.**  *517.*  **517-518.**  *518.*  **518-519.**  **518.**  *518.*  *518.*  **518-519.**  *518.*  **519.**  *519.*  **519.**  *519.* |

**Data Extraction 20.**

| Study Reference: Wand T., Glover S. & Paul D. (2022) What Should be the Future Focus of Mental Health Nursing? Exploring the Perspectives of Mental Health Nurses, Consumers and Allied Health Staff. *International Journal of Mental Health Nursing.* **31**, 179-188. | | |
| --- | --- | --- |
| Person extracting data: Donagh O’Brien. | Date data extracted: 08/06/24. | |
| Aim of Study: To explore the perspectives of mental health nurses (MHNs), consumers and allied health staff on the present and future status of mental health nursing. | | |
| Study design: Qualitative. Thematic Analysis. | | |
| Description of study setting/location(s): A local health district (LHD) in Sydney, Australia. | Study period (dates study was done): November 2020-December 2020. | |
| Sampling method (e.g. purposive, convenient, etc.): Convenience sampling. Email invitation sent to all staff at research location. | | |
| Inclusion and Exclusion criteria: None specific. | | |
| Final numbers included in the study: 11. | | |
| Description of the recruited sample (e.g. demographics, etc.) 5 senior MHNs with varying levels of experience and qualifications. 3 consumer representatives. 3 allied health professionals. | | |
| Description of data collection method (e.g. type of interviews, observations, location of data collection, etc.) Focus groups. 3 in total, one focus group with each group from sample. Focus groups lasted approx. 60 minutes. Focus groups consisted of questions about the current activities and roles of MHN, the main barriers and enablers for MHN in fulfilling their duties, local cultural issues in practice settings which impact MHN role, unique features of the MHN role and what should change about MHN role now and in the future? | | |
| Description of data analysis method (e.g. content analysis, thematic, etc.):  Thematic analysis. | | |
| **Findings:** All data that reflects views or experiences of the phenomenon under investigation | | Location/ page in text |
| **5 themes emerged from MHN focus group.**  **1: The health promoting MHN.**  Providing health information seen as central function of MHN. Information should be delivered in a therapeutic fashion incorporating interpersonal skills. Main objective is to give people enough information to make informed decisions.  *“I think it comes back to Knowledge is empowering. The more knowledge a person has, the more they can see how things feed into each other and with the more knowledge they can build, the more they can own their experience and how they respond to that experience and we do a lot of that as nurses, build on that knowledge and make it relatable to people.”*  **2: A person-centred approach.**  Participants acknowledge that nurses work in collaboration with patients.  *“What is it they want from this encounter? Not what do we want but what do they want from the encounter? I think nursing is probably better at doing that than certain other disciplines.”*  **3: Unique characteristics of the MHN role.**  Participants felt that MHN are uniquely flexible and adaptable with versatile skills and knowledge of medical, social, and human factors as well as support and advocacy.  *“We’re looking at the big picture all the time, perhaps that’s where our mastery is."*  *“Helping the person be in charge of their own health, making sure their voice is heard.”*  **4: An undervalued member of the team and service.**  Participants quite upbeat about their work and contribution. However, the saw the work of the MHN as undervalued and they were not consulted about clinical decisions or service decisions. They were often moved around to fill gaps.  *“There seems to be a lot done to you rather than for you or with you. Mental health nurse expertise is not valued at nursing management level.”*  **5: Refocusing the role of the MHN.**  Participants articulated what should be done to improve circumstances for MHN.  Greater planning needs to go into professional development for MHNs. Less emphasis on excessive bureaucracy and note writing and more on spending time with patients.  “The focus seems to be on audits, policy directives and mandatory training.” | | **181-182.**  *182.*  **182.**  **182.**  *182.*  *182.*  **182.**  *182.*  **182.**  *182.* |

**Data Extraction 21.**

| Study Reference: Rasmussen P., Conroy T. & Gronkjaer M. (2017) Child and Adolescent Mental Health Nurses’ Perceptions of their Professional Identity: An Exploratory Study. *The Journal of Mental Health Training, Education and Practice.* 12(**5**), 281-291. | | |
| --- | --- | --- |
| Person extracting data: Donagh O’Brien. | Date data extracted: 10/06/24. | |
| Aim of Study: To examine if it is applicable to use a conceptual framework for inpatient CAMH nursing practice to other areas of CAMH nursing practice. | | |
| Study design: Qualitative. Exploratory, within a social constructivist context. Thematic Analysis. | | |
| Description of study setting/location(s): Tasmania and Denmark. Local child and adolescent mental health services in both locations. | Study period (dates study was done): November 2014-December 2015. | |
| Sampling method (e.g. purposive, convenient, etc.): Purposive sampling used to recruit participants by invitation in 2 locations, Australia and Denmark. | | |
| Inclusion and Exclusion criteria: CAMHS nurses. | | |
| Final numbers included in the study: 9. | | |
| Description of the recruited sample (e.g. demographics, etc.) All women, with varied levels of experience from 8 months to 20 years of working with child and adolescent. Participants cover a broad range of roles. | | |
| Description of data collection method (e.g. type of interviews, observations, location of data collection, etc.) Focus groups, prior to which Participants were given an article by the author which described a conceptual framework for CAMH nurses. The framework describes the 5 stages of becoming a CAMH nurse. 1: Unknowing, 2: Cursory understanding of the known, 3: becoming known, 4: the known facilitates and 5: Understanding. Framework provided basis for focus groups. | | |
| Description of data analysis method (e.g. content analysis, thematic, etc.): Thematic analysis. | | |
| **Findings:** All data that reflects views or experiences of the phenomenon under investigation | | Location/ page in text |
| **3 Themes.**  **1: Individual preparation and experience of CAMH nurses.**  No educational pathway for CAMH nurses in Denmark whereas in Australia, all CAMH nurses have mental health nursing qualification. Mental health training available in Denmark but not mandatory.  *“When I started working in CAMHS I didn’t know much, but then I went up to stage 3 (advanced) of the framework, we are all on different stages in clinical practice.”*  Australian participants assumed everybody was at stage 3 due to the complexity of the cases.  Participants gain confidence as they move through the stages.  Both groups feel that it is important to have a mental health qualification to work in CAMHS.  **2: Knowledge transfer.**  Senior, more experienced nurses important for teaching less experienced to become CAMH nurses. Assessment and intervention skills of CAMH nurse dependant on their level of experience and expertise.  *“We use a shared body of knowledge, we recognise each other’s skill base, if I’m stuck, I can ask a colleague for a different perspective. Presentations are so complex. It’s a steep learning curve” (A4).*  Nurse’s role to educate other staff about CAMHS.  **3: Nurses’ perceptions of their individual and team roles.**  Danish nurses felt undervalued such as, not being invited to participate in decision making process, whereas membership of the multi-disciplinary team is a vital part of Australian nurse’s practice. Both sets of participants felt that nurses are fundamental to the running of both services.  *“The team is important to recognise each other’s skill base, to give a different perspective, but our contribution to the multidisciplinary team is not valued, we are not invited to the multidisciplinary perspective. Our voices are not included in the assessment and decision-making process.”* | | **284-285.**  *284*  **285.**  *285.*  **285-287.**  *286.* |

**Data Extraction 22.**

| Study Reference: Buescher T. & McGugan S. (2022) Standing Out on the Margins: Using Dialogical Narrative Analysis to Explore Mental Health Student Nurse Identity Construction and Core Modules. *Issues in Mental Health Nursing.* 43(**8**), 737-747. | | |
| --- | --- | --- |
| Person extracting data: Donagh O’Brien. | Date data extracted: 10/06/24. | |
| Aim of Study: To find out what are the experiences of mental health nursing students undertaking core modules (including students of all nursing disciplines) and to examine how core modules affect the professional identity formation of student mental health nurses. Based on evidence that mental health nursing students feel isolated in core modules. | | |
| Study design: Qualitative. Exploratory. Dialogical Narrative Analysis. | | |
| Description of study setting/location(s): Teaching Excellence Academy, Nursing course, University of Hull. | Study period (dates study was done): January 2019. | |
| Sampling method (e.g. purposive, convenient, etc.): Convenient sample. Recruited from university where researcher works. | | |
| Inclusion and Exclusion criteria: Mental health student nurses. | | |
| Final numbers included in the study: 6. | | |
| Description of the recruited sample (e.g. demographics, etc.) Six female student mental health nurses from 20s to 50s. 2 second year and 4 first year students. From a variety of academic backgrounds, 3 had prior degrees. 5 were parents. | | |
| Description of data collection method (e.g. type of interviews, observations, location of data collection, etc.) 1 focus group lasting 1 hour, facilitated by the Author. Brief schedule of questions in order to generate in-depth narrative responses from participants. | | |
| Description of data analysis method (e.g. content analysis, thematic, etc.): Data transcribed by hand. Dialogic narrative approach method used whereby transcripts were examined for emerging stories | | |
| **Findings:** All data that reflects views or experiences of the phenomenon under investigation | | Location/ page in text |
| **Findings took the form of stories, such as a perception by the sample of being chastised or silenced for showing ability to think critically or apply a mental health context. Other stories consisted of factors common with all nursing disciplines or organisational issues.**  **1: Outsiders.**  Participants reported feeling marginalised and excluded when doing core modules, even faced with hostility when speaking up about mental health in lectures.  *“Were seen as a bit quirky by general nurses. No-one from mental health ever speaks up in lectures. The lecturer thought that a woman with schizophrenia should have their children taken of her, just because she has schizophrenia. That shows that there’s an inadequate understanding of safeguarding.”*  **2: Free Thinkers.**  *“Self-reflection and self-care not taught adequately in core models.”*  **3: Professionals.**  In some shared lectures, participants felt more equal with other nursing disciplines. Some general nurses seemed interested in mental health lectures. Generic parts of the course lack mental health content.  “*General nurse training should include mental health component and vice versa. Its seen as if you need to be a specialist to provide care. Having core modules makes you think about the person as a whole, rather than to be compartmentalised.”*  **4: Practical Application.**  Students were not shown how to apply skills learned in core modules to day-to-day practice or were dismissed because they were mental health nurses.  *“We had a lecturer say straight up to us ‘you will never use this because you’re a mental health nurse’ and I thought it was pretty insulting and stupid to say because you do use it. You use all nursing skills in all fields so it’s a little bit silly and it just makes you feel a bit rubbish.”* | | **739-741.**  *741.*  **741.**  **741-742.**  *742.*  **742-743.**  *743* |

**Data Extraction 23.**

| Study Reference: Hercelinskyj G., Cruikshank M., Brown P. & Phillips B. (2014) Perceptions from the Front Line: Professional Identity in Mental Health Nursing. *International Journal of Mental Health Nursing.* **23**, 24-32. | | |
| --- | --- | --- |
| Person extracting data: Donagh O’Brien. | Date data extracted: 11/06/24. | |
| Aim of Study: 2 aims. 1: to identify ways in which mental health nursing could be promoted in order to help recruitment and retention and 2: to explore how nurse education could help increase the number of students choosing mental health nursing as a career. | | |
| Study design: Qualitative. Explorative descriptive design. | | |
| Description of study setting/location(s): Mental health services in Victoria, Australia. | Study period (dates study was done): Between 2005 and 2007. | |
| Sampling method (e.g. purposive, convenient, etc.): Purposive. | | |
| Inclusion and Exclusion criteria: To be a registered nurse, working in mental health services in Victoria, involved in clinical placements for students, mental health education and/or mental health research. Above stated as commonalities between participants rather than explicitly inclusion criteria. | | |
| Final numbers included in the study: 11. | | |
| Description of the recruited sample (e.g. demographics, etc.) 10 female and 1 male nurse. All participants primarily worked in mental health settings in Victoria, all with more than 5 years’ experience in mental health. | | |
| Description of data collection method (e.g. type of interviews, observations, location of data collection, etc.) Individual semi-structured, in-depth interviews. Participants asked to discuss their perceptions of their role as MHN, what are positive features of the role? how would you describe the role to others? And how do they believe others see their role? | | |
| Description of data analysis method (e.g. content analysis, thematic, etc.): Thematic analysis. | | |
| **Findings:** All data that reflects views or experiences of the phenomenon under investigation | | Location/ page in text |
| **4 themes emerged from data analysis.**  **1: Challenges to our role as Mental Health Nurse.**  Participants experienced role conflict or role stress in relation their expectations of their role vs a need to meet organisational outcomes. For example, perception that psychotic patient not given long enough admission due to need for high bed turnover.  *“The mental health act is supposed to be the least restrictive environment and I get frustrated. You have people in here, we hardly treat them, just jab them full of medication because there’s someone sicker that has to come in. It doesn’t take into account the 20-year old person with a first presentation psychosis who we now send home, whose parents may not be ready because we don’t want to cost the hospital money” (MHN 5).*  **2: Challenge in attracting the next generation of MHN.**  Participants worried about the future of MHN. Mental health nurse education not sufficient leaving graduates under prepared. Students should be actively engaged with whist on clinical placement to show them a much broader picture of MHN.  *“We need to show students the potential areas psych nurses can work in, the opportunities that can be available, and define what we do with clients. It’s not all about documentation, its not all about medicating, it’s the relational aspect of mental health nursing” (MHN 2).*  Participants felt that universities have a duty to promote MHN as a viable career option for students.  “There is not enough person-centred approach in nurse training. You need 12-18 months of person centred, psychosocial approach before you can look after somebody properly.” (MHN 8).  Participants felt that MHN need to “tell their story” to students.  *“I don’t think were very good at getting out there and promoting ourselves. I think we have to do that, otherwise we’re just going to fizz away like the intellectual disability nurses. We’re just going to fizzle out. I have almost a sense of urgency about it” (MHN 6).*  Participants felt they need to promote the broad nature of MHN and the positive outcomes on consumers.  *“I’d like to see a stronger picture of our professionalism and our clinical specialisation and an understanding of the knowledge and experience we have.” (MHN 3).*  Emphases idea that MHN don’t talk about their work.  **3: Being a team player.**  Participants said that working as part of a multi-disciplinary team can lead to a blurring of role boundaries and cause role ambiguity and role conflict.  *“I think that the nurse’s role has been eroded dramatically in the community. Making sure were all part of a team. The negative is that nurses are losing what it is that makes them separate in their professional identity.” (MHN 4).*  MHN role seems to lack clarity within the MDT.  Role expectations for MHN are at odds with reality. Socialisation and professional identity formation process are often impeded by organisational and government policies. MHN Role is constantly changing to keep up with this. Role uncertainty leads to professional identity uncertainty.  **4: Describing our work to others.**  Participants described the MHN role in different ways. Many highlighted the importance of interpersonal contact.  *“We provide comfort and support, we talk to the consumer who is so uptight, we offer them a cup of tea, provide explanation and education to them and make them feel a bit more comfortable.” (MHN 9).*  “Being with the patient is the basis of many activities.”  However, many participants found it difficult to describe the role of the MHN to others.  *“It’s always been the challenge to describe what you do to people that don’t know because unless people have had some kind of contact with mental illness or with the service provision for mental illness, its very hard for them to relate because they’re coming from their preconceptions” ( MHN 3).*  Professional identity is developed through professional and education socialisation. People’s understanding of any discipline of nursing can only develop through direct or indirect contact with nurses. | | **26-27.**  *27*  27.  27.  27.  **28.**  28.  28.  **28.**  *28*  *28*  *29.* |
