# Supplementary material for: A Systematic Review of Mental Health Nurses' Perceptions of Their Professional Identity
Source: Int J Ment Health Nurs. 2025 Sep 24;34(5):e70137. doi: 10.1111/inm.70137 (PMC12459084; doi:10.1111/inm.70137)
Supplement: Supplementary file 4 — Data S4: inm70137‐sup‐0004‐DataS4.docx. [file INM-34-0-s001.docx]

Supplementary Info 4: Table showing study selection based on inclusion and exclusion criteria.

| **Inclusion/exclusion criteria.**  **Paper** | **Sample.** | **Phenomenon of Interest.** | **Design.** | **Evaluation.** | **Research Type.** |
| --- | --- | --- | --- | --- | --- |
| 1: Sercu et al. (2015). | * | * | * | * | * |
| 2: Barlow (2006). | * | * | * | * | * |
| 3: Rungapadiachy et al. (2004). | * | * | * | * | * |
| 4: Deady (2005). | * | * | * | * | * |
| 5: Karanikola et al. (2018). | * | * | * | * | * |
| 6: Rasmussen et al. (2017). | * | * | * | * | * |
| 7: Terry (2020). | * | * | * | * | * |
| 8: McCrae et al. (2014). | * | * | * | * | * |
| 9: Hurley & Lakeman (2011) | * | * | * | * | * |
| 10: Hurley (2009). | * | * | * | * | * |
| 11: Crawford et al. (2008). | * | * | * | * | * |
| 12: Hercelynsky et al. (2014). | * | * | * | * | * |
| 13: Moir & Abraham (1996) | * | * | * | * | * |
| 14: Santangelo et al. (2017). | * | * | * | * | * |
| 15: Harrison et al. (2017). | * | * | * | * | * |
| 16: Wand et al. (2021) | * | * | * | * | * |
| 17: Buescher & McGugan (2022). | * | * | * | * | * |
| 18: Savio (1991) | * | * | * | * | * |
| 19: Humble & Cross (2010) | * | * | * | * | * |
| 20: White & Kudless (2008). | * | * | * | * | * |
| 21: Crawford et al. (2008). | * | * | * | * | * |
| 22: Bray (1999). | * | * | * | * | * |
| 23: Holyoake (2002). | * | * | * | * | * |
