# Supplementary material for: A Systematic Review of Mental Health Nurses' Perceptions of Their Professional Identity
Source: Int J Ment Health Nurs. 2025 Sep 24;34(5):e70137. doi: 10.1111/inm.70137 (PMC12459084; doi:10.1111/inm.70137)
Supplement: Supplementary file 5 — Data S5: inm70137‐sup‐0005‐DataS5.docx. [file INM-34-0-s002.docx]

**Findings.**

Following data analysis, 5 main themes emerged from the data: 1: Professional identity formation, 2: The attributes of mental health nurses, 3: The mental health nurse role, 4: The unique skills and knowledge of mental health nurses, and 5: Professional identity and the future of mental health nursing.

# **Theme 1: Professional Identity Formation.**

The first theme focused on aspects of PI formation for mental health nurses, their reasons for choosing mental health nursing as a career and what factors may have positively or negatively influenced PI formation. Many participants made the choice to pursue a career in mental health or psychiatric nursing by contrasting it with general nursing and identifying key differences between both disciplines. Mental health nursing was viewed as attractive by some because it was seen as less technical and less restrictive than general nursing.

*“I enjoyed it (my mental health nursing placement) so much more than my general when I’ve been going through my training, and I really feel like I’m the kind of person who likes to sit and talk to patients. You have time in psyche. It’s not so technical orientated.” (Moir and Abraham, 1996)*.

Similarly, qualified nurses cited their choice of mental health nursing as a career due to general nursing not meeting their expectations. One participant felt that interactions with patients were not appreciated by general nurses. In contrast, they were attracted to a more patient focused, interpersonal approach.

*“After I choose psychiatry and that was it. Just the fact that you don’t wear a uniform and the way they dealt with service users, really working with them, that’s what really attracted me” Hanne. (Sercu et al., 2015)*.

A formative step towards PI is the act of choosing mental health nursing as a career. The data gave a broad indication of the reasoning behind that choice for mental health nurses. Once a mental health nurse has chosen their career path by whatever means, the process of PI formation begins in earnest. Participants identified factors, such as their work with patients, and the learned roles and knowledge they gained from experienced staff during the socialisation process as key to their nursing identity formation. The role of education and training was also emphasised.

*“I think things like role modelling and coaching, exposure to good clinicians, getting a chance to hear these good clinicians talk out loud about what they do. Working with people and getting feedback has also helped” (Research Participant 12*). (Hurley and Lakeman, 2011).

The educational experience was not the same for everyone. Some mental health student nurses felt marginalised and made to feel different by university general nursing academic staff when they took a core nursing module with general nurses. General nursing academic staff reportedly lacked knowledge about mental health and mental health nursing students were chastised for speaking up or having critical thinking ability. Thus, potentially leading to feelings of isolation among the participants and potentially having a negative impact on their PI.

*“Were seen as a bit quirky by general nurses. No-one from mental health ever speaks up in lectures. The lecturer thought that a woman with schizophrenia should have their children taken of her, just because she has schizophrenia. That shows that there’s an inadequate understanding of safeguarding.”* (Buescher and McGugan, 2022).

The data on PI formation for mental health nurses suggested that people chose to be mental health nurses for many reasons, and they were socialised into their profession by exposure to clinical practice, role models and experience. Mental health nurses made a clear distinction between themselves and general nurses, perhaps the first step towards a PI.

# **Theme 2: The Personal Attributes of Mental Health Nurses.**

Many of the included studies suggested that mental health nurses perceived themselves as having attributes or characteristics that both predisposed them to a career in mental health nursing and were necessary for them to do their jobs effectively. These personal attributes were perceived by some as almost unique to mental health nursing and a key part of the PI. Mental health nurses in some studies viewed themselves as having attributes and attitudes, which they perceived to be different to other nursing disciplines. Pragmatism, flexibility, common sense, being down-to-earth and being able to approach patients on an equal footing were commonly reported characteristics.

*“Community Psychiatric Nurses (CPNs) are patient, practical, flexible and with a good level of common sense. CPN is a translator on the patient’s level.”* (Barlow, 2006).

Participants in the studies conducted by Deady (2005) and Humble and Cross (2010) viewed themselves as different to other nursing disciplines, with mental health nurses in the latter study relishing the difference, seeing themselves as more liberal, less judgemental and with less susceptibility to being shocked than other branches of nursing.

*“I think you have to be able to accept a bit of a shock or you wouldn’t stay in the job, so that makes us a bit different because I’ve found a lot of nurses are very conservative. I think we are a different breed because we aren’t shocked or horrified by what’s at work every day” (Elizabeth). (Humble and Cross, 2010)*.

Gender identity and societal views about the characteristics or attributes associated with gender were seen in some studies as influential on mental health nurse identity, arguably bordering on stereotype.

*“I think that society actually views women as being more sort of nurturing and caring and that it’s a special type of male that actually comes into nursing in the first place. There are more males in psychiatry, and I think this is historical, taken from the old days of Victorian asylums” (Anthony).* (Holyoake, 2002).

In reporting on the attitudes of Irish psychiatric nurses, Deady (2005) stated that participants believed that general nurses as well as society viewed psychiatric nursing as inferior because there are more men doing the job. Holyoake (2002) stated that at the time of writing of his study, 10% of the nursing workforce was male but 35% of management positions in nursing were taken by male nurses. Similarly, participants from the study by Crawford et al. (2008) seemed to dispute the idea that there are more male psychiatric nurses but still adhered to stereotypical views on gender in nursing.

*“Traditionally, it’s been that women tend to be less assertive, tolerate more, for want of a better word, crap than men will. It’s seen as a female role, its’s nurturing, caring, it’s a badly paid job. So, it’s low pay and men won’t do it. And if they do it, it’s at management level away from nursing” (Crawford et al., 2008)*.

Based on this evidence, mental health nurses believed they possessed attributes which made them more suited to the role than other nurses. Such attributes and attitudes are not necessarily unique but contribute to their social identification. Conversely, gender-based attributes are more likely to be imposed on nurses by society but the nurses in the studies seemed to comply with them (Holyoake, 2002).

# **Theme 3: The Mental Health Nurse Role.**

The role of the mental health nurse was mentioned in all 23 included studies in a variety of contexts. Role, in this case, refers to the practicalities of mental health nursing, notably, what it is that a mental health nurse actually does in the workplace. In many of the studies, the concept of the role of the mental health nurse and the concept of their PI were closely aligned.

Student mental health nurses spoke about what they perceived as the role of qualified mental health nurses, based on their observations of qualified nurses during their clinical placements. Among their perceptions of the mental health nurse role were that qualified mental health nurses engage in activities such as ward management, doing paperwork, advocating for patients and liaising on their behalf with other patents, giving handovers, attending ward rounds and administering medication (Rungapadiachy et al., 2004). Practical nursing duties such as medication administration, conducting domiciliary visits and giving injections were also identified as constituents of the mental health nurse role (Savio, 1991, Barlow, 2006, Karanikola et al., 2018, Hurley, 2009). The mental health nurse role was described in much broader terms, as captured by a participant in the study by Hurley (2009).

*“The nurse, who is perhaps doing some sort of loose term psychological supportive therapy one moment, might the next moment be taking their pulse, or dressing a wound, and the next moment, might actually be helping them to sort their housing and the next moment possibly having a game of scrabble with that same person. Now I’ve never seen a psychotherapist, or, you know a clinical psychologist playing scrabble or fill in a housing form.” (RP 24).* (Hurley, 2009).

Elsewhere, the caring aspects of the mental health nurse role and patient education were viewed as essential to the mental health nurse role.

*“We provide comfort and support, we talk to the consumer who is so uptight, we offer them a cup of tea, provide explanation and education to them and make them feel a bit more comfortable.” (MHN 9). (Hercelinskyj et al., 2014)*.

The idea that mental health nurses act as translators of intimidating medical language to something more understandable to their patients was mentioned by Barlow (2006) and in the context of the mental health nurse as health promotor by (Wand et al., 2022).

*“I think it comes back to Knowledge is empowering. The more knowledge a person has, the more they can see how things feed into each other and with the more knowledge they can build, the more they can own their experience and how they respond to that experience, and we do a lot of that as nurses, build on that knowledge and make it relatable to people.” (Wand et al., 2022)*.

The everyday work of a mental health nurse is therefore quite varied. The variation inherent in the mental health nurse role however, made it appear ambiguous and not easy to define or describe. This ambiguity created a lack of clarity about the mental health nurse’s sense of PI. Rasmussen et al. (2017) stated that PI is important as it gives mental health nurses a better understanding of their role, helps them identify a theoretical framework for their work and helps them determine which aspects of their role are specific to nursing. However, PI was difficult for mental health nurses to articulate.

*“Professional Identity? I find that quite difficult to answer. What my exact role is. I tend to say that I’m kind for a living. I help people to help themselves. I try to enable people, empower people” (RP 019).* (Crawford et al., 2008).

A key consequence of the multi-faceted nature of the mental health nurse role was its invisibility, the fact that nobody sees the mental health nurse carrying out their most important duties. Or that the most important work that a mental health nurse does might simply consist of talking to a patient, which may or may not be perceived as legitimate work.

*“Sitting and talking with patients are not always activities that are clearly visible to others, on the face of it, it looks like they are just watching TV, but they are actually sitting and talking to somebody” (Participant 5). (Rungapadiachy et al., 2004)*.

Working within a team was a common thread throughout the studies, participants accepted the value of a team environment but felt underestimated by the team.

*“Our contribution to the multidisciplinary team is not valued, we are not invited to the multidisciplinary perspective. Our voices are not included in the assessment and decision-making process.” (Rasmussen et al., 2017)*.

Some participants were more overtly resentful about their lack of power within their organisation, especially given their perceived importance to its running. Similarly, community mental health nurses felt undervalued and unrecognised despite their perception that they provide an invaluable service.

*“We don’t have any clout. We answer to everybody, a doctor or social worker can compulsorily admit someone to hospital but we can’t. But when everything goes wrong, it’s us who have to fix it. If were supposed to be mental health specialists, why does nobody ask our opinion? ”* (RP 001). (Crawford et al., 2008).

*“We are not recognised for the complex care we can provide, even in the medication clinics. We don’t just hand out medications, we always assess and evaluate clients when we see them. We provide valuable information to team members and case managers about the client’s progress.”* (White and Kudless, 2008).

The lack of status of mental health nurses in what duties they can perform compared to other disciplines was a cause of frustration for a participant, again given that they perceived themselves as more essential and worthy of higher status than the other disciplines. Similarly, mental health nurses encountered negative societal attitudes on mental health nursing but were nevertheless undeterred in their goal to progress in their careers.

*“I just get the sense that nurses have had everything taken away from them. They’ve been left with this position of supposed professional status, but actually we can’t do anything. OTs, physiotherapists, doctors, social workers, they’re the ones with the proper roles, and it seems to me the nurse in the middle coordinates it.” (Laura)* (Terry, 2020).

*“I feel very passionate about nursing as a career. I had a few status issues with it before, which sounds really naff, but I came from a really posh school. I went back to my ten-year reunion and everyone’s a lawyer. Nursing was never considered a respectable qualification but having done it, it’s literally the best thing.”* (McCrae et al., 2014).

It is evident from the data that some mental health nurses perceived themselves as having low status or being undervalued. Barlow (2006) also sought testimony from other non-nursing members of a mental health team about the role of the CPN, most of whom agreed that the CPN is highly valued, with excellent problem-solving skills, a valuable knowledge base and an aptitude for critical thinking, despite the CPNs appearing to think the exact opposite. Barlow (2006) stated that the non-nursing team members recognised the CPNs’ input to the team more than the CPNs did. Crawford et al. (2008) described how participants in their study were self-effacing in their attitude towards their achievements with patients leading to their work being invisible and them not receiving due credit. The concurrent desire for approval or recognition was described by participants in the same study.

*“So much of feeling good about your work and yourself when it comes down to it depends on whether other people appreciate it, like when the patients say thank you or if your manager does, if that was ever going to happen which seems pretty unlikely, but at the end of the day you’re just so dependent on the approval of other people” (RP 31).* (Crawford et al., 2008).

# **Theme 4: The Unique Skills and Knowledge of Mental Health Nurses.**

Contrary to the idea of the day-to-day activity of mental health nursing being of low status or invisible, Santangelo et al. (2018) argued that mental health nursing consists of distinctive skills, knowledge and expertise, and consequently, is specialist, with a broad scope and a high level of influence. Some of the data suggested some key factors which make a case for mental health nursing as unique compared to other nursing disciplines or professions.

Hurley (2009) stated that 79% of participants in his study said that mental health nurses spend more time with patients than other disciplines. Santangelo et al. (2018) stated that mental health nurses bring a new construct to the caring role, characterised by collaborative relationships which bring patient focus to a new level. The patient focus of mental health nurses was reflected very clearly in the data.

*“Everything we do here is to socialise the user, for that goal. Whether it is medication, a chat, a visit, manual activities, therapeutic follow up, any intervention, it is with the intention of improving their quality of life.” (Joao)* (Reis et al., 2023).

Mental health nurses in the study by Bray (1999) where the research participants were engaged in close observation of difficult patients, remained highly motivated and patient focused, despite their work taking an emotional toll. Other participants in the same study highlighted the role they have in helping patients feel validated or accepted.

*“We’re able to offer people a place where they feel safe. There’s a chance to talk to people who care about what they’re going through. Being there and being able to listen, they don’t feel valued and that’s what they need.” (Bray, 1999)*.

The patient-centred aspect of mental health nursing was also discussed in another study.

*“What is it they want from this encounter? Not what do we want but what do they want from the encounter? I think nursing is probably better at doing that than certain other disciplines.” (Wand et al., 2022)*.

The therapeutic relationship is described as both a unique and definitive aspect of mental health nursing practice. Community mental health nurses viewed the formation of a therapeutic relationship with a patient and their family as one of the most important parts of their role.

*“The rapport you build with a patient is your main therapeutic tool” (Nina). (Karanikola et al., 2018)*.

In contrast to the idea of mental health nurses having clear and specific specialist skills, the idea of them operating with a varied and unspecific set of skills was a common theme in the data. The term “Jack of All Trades” appeared in some of the studies and reflected the diversity of the mental health nurse role but also its low professional status (Hurley, 2009). However, it was seen by some mental health nurses as positive in some cases and a source of professional pride and as a negative my others

*“We are separate to other disciplines. We’re not particularly intimidated or don’t feel threatened constantly by people who are potentially aggressive. With medical nursing, where there’s a physical problem, that’s what they’re there to do, they don’t look at other issues. We’ve a greater range of skills (Robert).”* (Humble and Cross, 2010).

“*I think that you are a “Jack of All Trades”, master of none in some respects, or you become very exceptional in the area in which you work.” (Emma) (Terry, 2020)*.

“*I agree that you need to be in some level a specialist in what you do, rather than a “jack of all trades.” (Angela) (Terry, 2020)*.

Some of the data suggested that mental health nurses have constructed their PI by taking skills and knowledge from other healthcare disciplines. Terry (2020) suggested that mental health nursing might steal aspects of other healthcare roles and subsume them into their own identity. A participant felt that borrowing skills from other disciplines and repackaging them as mental health nursing is a special skill and worthy of recognition.

*“We have garnered many things from other professions to make up what we are. It’s that bit about bringing many things together and it is how we put them together. There’s no other profession that combines all these different things in the way mental health nursing does and then delivers it back.” (RP8) (Hurley, 2009)*.

Similarly, mental health nurse participants did not think that they were in competition with other disciplines or could claim ownership of certain skills, rather they hold their own unique place within healthcare systems. Therefore, the idea that mental health nursing is made up of practices which other disciplines also practice, is not viewed as negative (Santangelo et al., 2018).

# **Theme 5: Perceptions of Professional Identity and the Future of Mental Health Nursing.**

Taking into account the complexity of mental health nursing, as described by the nurses themselves, some of the data indicates how mental health nurses view their PI, and what they see as the status of their profession and how to improve their status. A portion of the data showed how mental health nurses, particularly in Australia, felt about their identity after many years of generic nurse training.

*“I actually think that the nursing role has been eroded quite dramatically in the community to this generic title. Nurses are starting to lose what it is that makes them separate in their professional identity” (MHN no 4) (Hercelinskyj et al., 2014)*.

Buescher and McGugan (2022) perhaps showed how mental health nursing education within a generic nursing curriculum might look, with minimal mental health content within core modules and mental health nursing students effectively excluded.

Mental health nursing is unpopular as a career choice for student nurses due to its weak PI, its poorly understood role, its invisibility, and the stigma associated with it (Harrison et al., 2017). The culmination of the unpopularity is recruitment and retention problems and the potential extinction of the profession. A participant in one study acknowledged that mental health nurses could do more to improve their standing.

*“I don’t think were very good at getting out there and promoting ourselves. I think we have to do that, otherwise we’re just going to fizz away like the intellectual disability nurses. We’re just going to fizzle out. I have almost a sense of urgency about it” (MHN 6) (Hercelinskyj et al., 2014)*.

Another study found that unmotivated and negative mental health nursing profession was contributing to the recruitment of mental health nurses and that self-promotion was imperative. The lack of specialist status was also seen as problematic for the future of mental health nursing in this study.

*“Mental health nursing should be made a proper specialty. It certainly isn’t taught well on the wards. When the mental health nurses all retire there will be no specialist nurses left.” (Harrison et al., 2017)*.

Wand et al. (2022) stated that the culture in which mental health nurses work must change with more emphasis on patient care, rewards for nurses who actively engage in professional development and less emphasis on excessive paperwork. Hercelinskyj et al. (2014) stated that mental health nurses experience role conflict when they are caught between keeping up with organisational demands, such as documentation, and their patient care role. This role conflict leads to an unclear PI. The whole picture of mental health nursing must be promoted if the profession is to continue.

*“We need to show students the potential areas psych nurses can work in, the opportunities that can be available, and define what we do with clients. It’s not all about documentation, it’s not all about medicating, it’s the relational aspect of mental health nursing” (MHN 2) (Hercelinskyj et al., 2014)*.

The future of nursing is therefore dependant on active promotion of the discipline by the current cohort of mental health nurses. Santangelo et al. (2018) suggested a reframing of what we already know about mental health nursing and proposed a substantive model of care. The aim of this new proposed model was to promote a broader appreciation of the distinctiveness and importance of mental health nursing, simply by creating an identity which was more understandable and easier to articulate to others. (Santangelo et al., 2018).

*“Being in the here and now, side-by-side, co-constructing care.” (Santangelo et al., 2018)*.
